# Automatic detection of squamous cell carcinoma metastasis in esophageal lymph nodes using semantic segmentation

**DOI:** 10.1002/ctm2.129

**Published:** 2020-07-28

**Authors:** Yi Pan, Zhuo Sun, Wenmiao Wang, Zhaoyang Yang, Jia Jia, Xiaolong Feng, Yaxi Wang, Qing Fang, Jiangtao Li, Hongtian Dai, Calvin Ku, Shuhao Wang, Cancheng Liu, Liyan Xue, Ning Lyu, Shuangmei Zou

**Affiliations:** ^1^ Department of Pathology, National Cancer Center/Cancer Hospital Chinese Academy of Medical Sciences and Peking Union Medical College Beijing China; ^2^ Thorough Images Beijing China

**Keywords:** esophagus, lymph node metastasis, semantic segmentation, squamous cell carcinoma

## Abstract

Esophageal squamous cell carcinoma (ESCC) is more prevalent than esophageal adenocarcinoma in Asia, especially in China, where more than half of ESCC cases occur worldwide. Many studies have reported that the automatic detection of lymph node metastasis using semantic segmentation shows good performance in breast cancer and other adenocarcinomas. However, the detection of squamous cell carcinoma metastasis in hematoxylin‐eosin (H&E)‐stained slides has never been reported. We collected a training set of 110 esophageal lymph node slides with metastasis and 132 lymph node slides without metastasis. An iPad‐based annotation system was used to draw the contours of the cancer metastasis region. A DeepLab v3 model was trained to achieve the best fit with the training data. The learned model could estimate the probability of metastasis. To evaluate the effectiveness of the detection model of learned metastasis, we used another large cohort of clinical H&E‐stained esophageal lymph node slides containing 795 esophageal lymph nodes from 154 esophageal cancer patients. The basic authenticity label for each slide was confirmed by experienced pathologists. After filtering isolated noise in the prediction, we obtained an accuracy of 94%. Furthermore, we applied the learned model to throat and lung lymph node squamous cell carcinoma metastases and achieved the following promising results: an accuracy of 96.7% in throat cancer and an accuracy of 90% in lung cancer. In this work, we organized an annotated dataset of H&E‐stained esophageal lymph node and trained a deep neural network to detect lymph node metastasis in H&E‐stained slides of squamous cell carcinoma automatically. Moreover, it is possible to use this model to detect lymph nodes metastasis in squamous cell carcinoma from other organs. This study directly demonstrates the potential for determining the localization of squamous cell carcinoma metastases in lymph node and assisting in pathological diagnosis.

## Graphical abstract

XXX

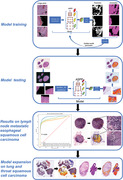

## 1 INTRODUCTION

There is a significant difference between Asian and Western nations with respect to the following two esophageal cancer histology types: esophageal squamous cell carcinoma (ESCC) and esophageal adenocarcinoma (EAC). Although EAC predominates in the United States, in Asia, especially in China, most esophageal cancer cases (95%) are ESCCs.^1^, ^2^ More than half of patients present with metastases or unresectable disease,^3^ which leads to a dismal 5‐year survival rate that, although has increased over time, remains a mere 18%.^4^ The incidence of lymph node metastasis (LNM) in ESCC is reported to be approximately 38.2‐43%.^5^ LNM is one of the most important prognostic factor for ESCC patients. Therefore, accurate assessment of lymph node status is very important for both early and advanced lesions. Although there are various clinical diagnosis methods, the clinical evaluation of lymph node status is still not ideal.^6^ Therefore, a more reliable tool is urgently needed for both early and advanced ESCC.

Recently, many artificial intelligence approaches have been proposed to automatically detect metastasis in esophageal lymph nodes. Several methods based on clinical or radiomic features have been proposed for esophageal LNM detection^7^, ^8^, ^9^, ^10^, ^11^, ^12^; however, these methods fail to reflect morphological changes or metabolic changes due to metastasis. In pathology practice, several studies have reported that the automatic detection of LNM showed good performance in breast cancer and other adenocarcinomas.^13^, ^14^, ^15^, ^16^, ^17^ However, obtaining more detailed pathological information from slides for LNM detection applications in squamous cell carcinoma has never been explored.

Since the introduction of the open access The Cancer Genome Atlas (TCGA)^18^, ^19^ and CAMELYON^20^ datasets, computational pathology has dramatically expanded its capabilities with the help of deep learning,^21^ and various types of state‐of‐the‐art convolutional neural networks (CNNs) have been applied to high‐resolution hematoxylin‐eosin (H&E)‐stained pathology whole slide images (WSIs). Due to the high incidence of breast cancer, most studies focused on breast tumors^13^, ^14^, ^15^ or LNM.^16^, ^17^ In both cases, deep learning‐based methods reported promising results and showed potential clinical usage. However, these methods focus on adenocarcinoma, which is the dominant subtype of breast cancer and gastric‐intestinal cancer. As far as we know, the detection of squamous cell carcinoma via H&E‐stained WSIs has not been a research focus, even though it is the predominating subtype of esophageal cancer in Asia.

In this study, we focused on squamous cell carcinoma metastasis, especially in esophageal cancer patients. Due to the lack of a WSI dataset of squamous cell carcinoma metastasis, we performed a study to collect and annotate a large number of lymph nodes with squamous cell carcinoma metastasis in esophageal cancer patients undergoing surgery. Using these annotated WSIs, we trained a state‐of‐the‐art CNN that can automatically segment the metastatic region in esophageal lymph node WSIs. We tested the obtained model using a much larger test set of esophageal lymph node WSIs. Furthermore, we attempted to apply our model to squamous cell carcinoma in other organs to identify its potential in determining the localization of lymph node metastases in squamous cell carcinoma and assisting in pathological diagnosis.

## 2 MATERIALS AND METHODS

### 2.1 Pathologists

In total, 10 pathologists participated in this study as readers. These pathologists did not involve in the reference standard classification. Their experience in pathology range from 1 to 20 years. None of the pathologists specialize in esophageal pathology, and all pathologists have extensive clinical practice of anatomic pathology, including a review of lymph node specimens of esophageal cancer cases.

### 2.2 Training data

In this study, we collected 242 esophageal lymph node WSIs (110 WSIs with squamous cell carcinoma metastasis and 132 WSIs without metastasis) from the Cancer Hospital, Chinese Academy of Medical Sciences and Peking Union Medical College. Originally, based on the largest diameter of the largest focus of metastases in the lymph nodes, metastasis >2.0 mm in diameter was defined as macro‐metastasis, and metastasis >0.2 mm but 2.0 mm or less was defined as micro‐metastasis.^22^ The current study was approved by the ethics committees of Clinical Research Ethics of the Cancer Hospital, Chinese Academy of Medical Sciences and Peking Union Medical College.

### 2.3 Test data

To better evaluate the performance of the proposed method, we included 795 esophageal lymph node WSIs (222 with metastasis and 573 without metastasis) from 154 esophageal cancer patients who underwent surgery at the Cancer Hospital, Chinese Academy of Medical Sciences and Peking Union Medical College between April 2017 and May 2018 as our main test set.

In addition to the esophageal lymph node WSIs, we collected 30 lung lymph node WSIs (nine positive and 21 negative) and 30 throat lymph node WSIs (20 positive and 10 negative). All positive WSIs contained squamous cell carcinoma metastasis.

### 2.4 Annotation

**FIGURE 1:**
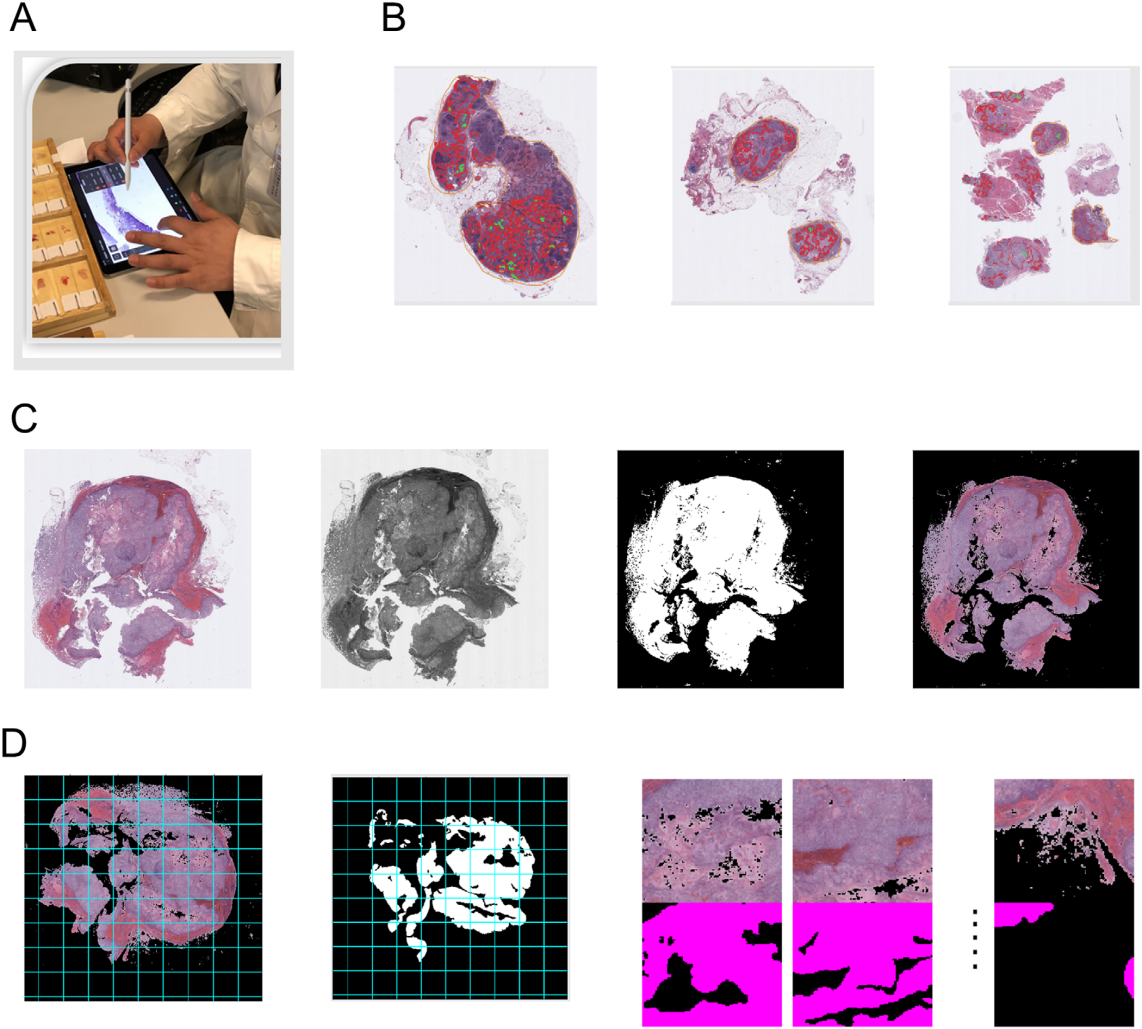
Illustration of the annotation and image preprocessing. A, The pathologist made manual annotations using an in‐house iPad pathology annotation application. B, Examples of the annotated WSIs. Red contours cover the region of metastasis, and green contours cover the normal region embedded inside the metastasis. C, Original WSI, gray level image, tissue mask, and masked tissue region. D, WSI and annotation mask cut into patches and training patch pairs

The WSIs with metastasis were manually annotated by the pathologists involved in this study using an in‐house‐developed, iPad‐based application as shown in Figures 1A and 1B. During the annotation step, each positive WSI was first annotated by one pathologist, followed by a second pathologist to correct any annotation mistakes and identify potentially missing metastatic regions. These two rounds of annotation are similar to those applied in clinical practice for traditional pathological diagnosis. For the negative WSIs, because there is no metastasis, we did not use a manual annotation and simply treated all regions in the WSIs as nonmetastatic.

### 2.5 Image preprocessing

For each WSI, we converted the color image into a gray value image and used the Otsu threshold^23^ to generate a tissue mask. Using this tissue mask, we can largely reduce the application of the impractical training patch and the computation cost during the inference phase in real applications. For the training, the WSI and corresponding annotation mask under the tissue mask were cut into many equal‐sized patch pairs as shown in Figures 1C and 1D. Overall, we generated 324 264 patch pairs from the training set; the detailed statistics on both patch and pixel level is shown in Table S1.

### 2.6 Training the model

In this study, we used a state‐of‐the‐art CNN model to segment the metastatic region. More specifically, we used the DeepLab model^24^, ^25^ with ResNet‐50^26^ as the backbone network due to its high performance in the natural image segmentation task. Due to the large imbalanced distribution of the training set in which the nonmetastatic region was much larger than the metastatic region, we used the focal loss^27^ instead of the commonly used cross entropy as the correction to overcome the imbalance problem. During the training phase, we set the size of the train patch to 320 × 320 to increase the number of patches in each batch.

### 2.7 Inference and testing

After training the model, the learned model parameters were fixed during the inference phase, and we directly applied the model to any new WSI during the testing. Similar to the training phase, each WSI was first preprocessed to reduce the computation cost of the nontissue region. Then, the tissue portion of the test image was divided into an image patch and passed through the model to obtain the segmentation result. In practice, the size of the image patch during the inference phase can differ from and be much larger than that during the training phase to more efficiently use the computation and I/O.

### 2.8 Model extension

Because the lung lymph node set and throat lymph node set also contain squamous cell carcinoma metastasis, we detected metastasis in these two anatomies using the model trained by the esophageal lymph nodes without modifying the model.

### 2.9 Statistical analysis

In this study, we used the arear under the curve (AUC) to compare the performances of different models that distinguish esophageal lymph node WSIs with and without metastasis. The receiver operating characteristic (ROC) curve represents the relationship between the sensitivity and the false positive rate, namely 1‐specificity.^28^ The AUC ranges from 0 to 1 to indicate the performance of the classifier. Using a perfect classifier, the AUC is 1, and by chance, the AUC is 0.5. Furthermore, we used the percentile bootstrap method^29^ to compute the 95% confidence interval (CI) of the ROC curves. To statistically compare the performances of the different models at the slide level using the main esophageal lymph node test set, we used the two‐tailed bootstrap‐based method described by Hanley et al,^30^ which considers the correlation of the paired nature of the data. A *P*‐value < .05 was considered significant.

## 3 RESULTS

### 3.1 Network structure of the training model

**FIGURE 2:**
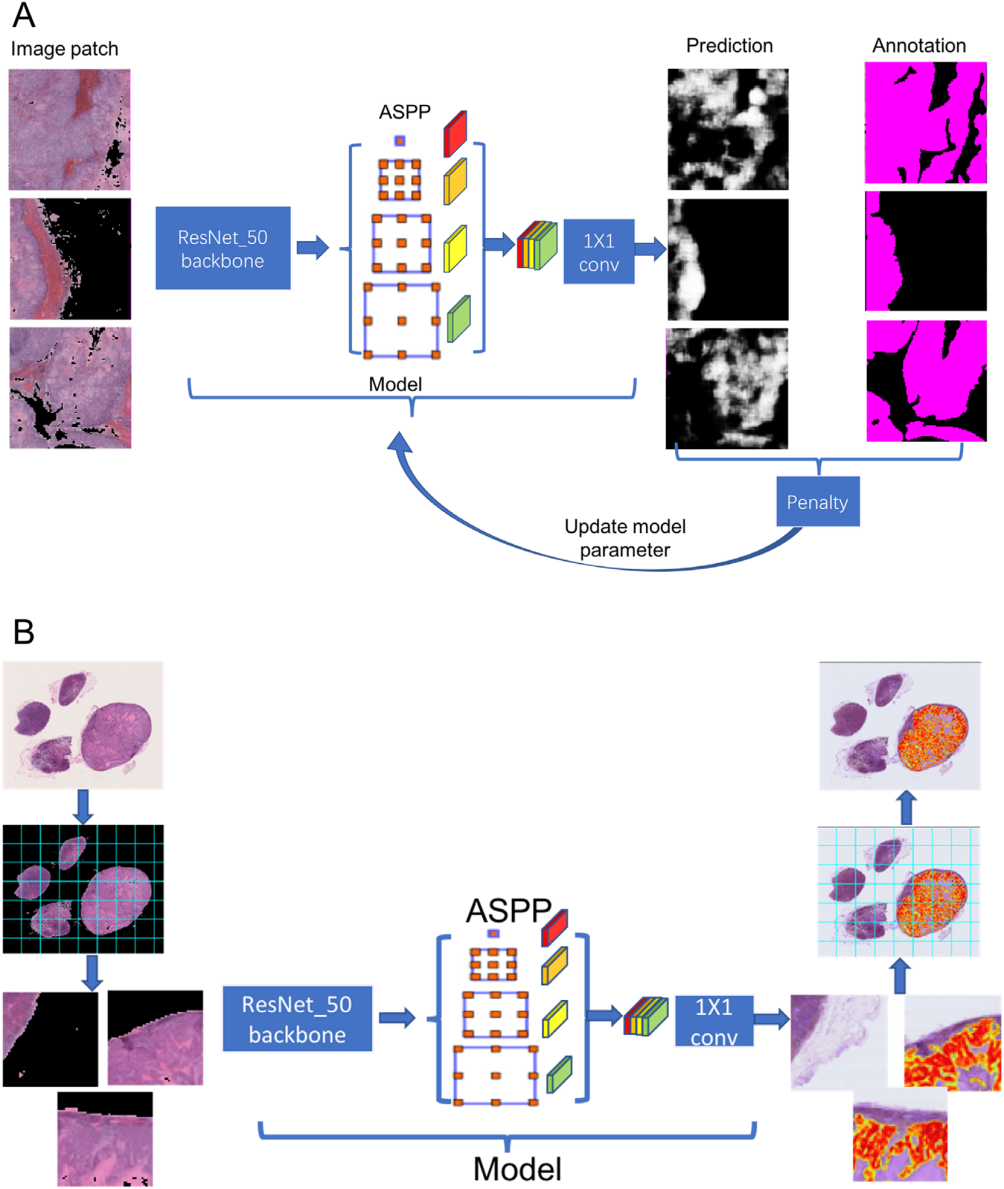
Model training and testing phases. Illustration of (A) training the DeepLab v3 network and (B) the inference procedure used to the test the WSIs

Compared to DeepLab v2,^24^ DeepLab v3^24^ has an atrous spatial pyramid pooling structure capable of gathering useful information from different image scales as shown in Figure 2A. During the training, in each iteration, a batch of randomly sampled training patch pairs were used. The image patches were fed to the network, and the difference between the network outcome and annotation patches was corrected to optimize the parameters in the network.

### 3.2 Slide‐level scoring of the testing set

Using the training data, we trained the following three models: (a) DeepLab v2 with cross entropy loss, (b) DeepLab v3 with cross entropy loss, and (c) DeepLab v3 with focal loss. In each model, only the network (DeepLab v2 or v3) and loss differed. After the training, each test WSI was subjected to each model to generate a pointwise probability map as shown in Figure 2B. We computed the mean of the largest 1000 probabilities in the resulting probability map as the prediction score of the WSI.

### 3.3 Patients’ clinical features of the testing set

**FIGURE 3:**
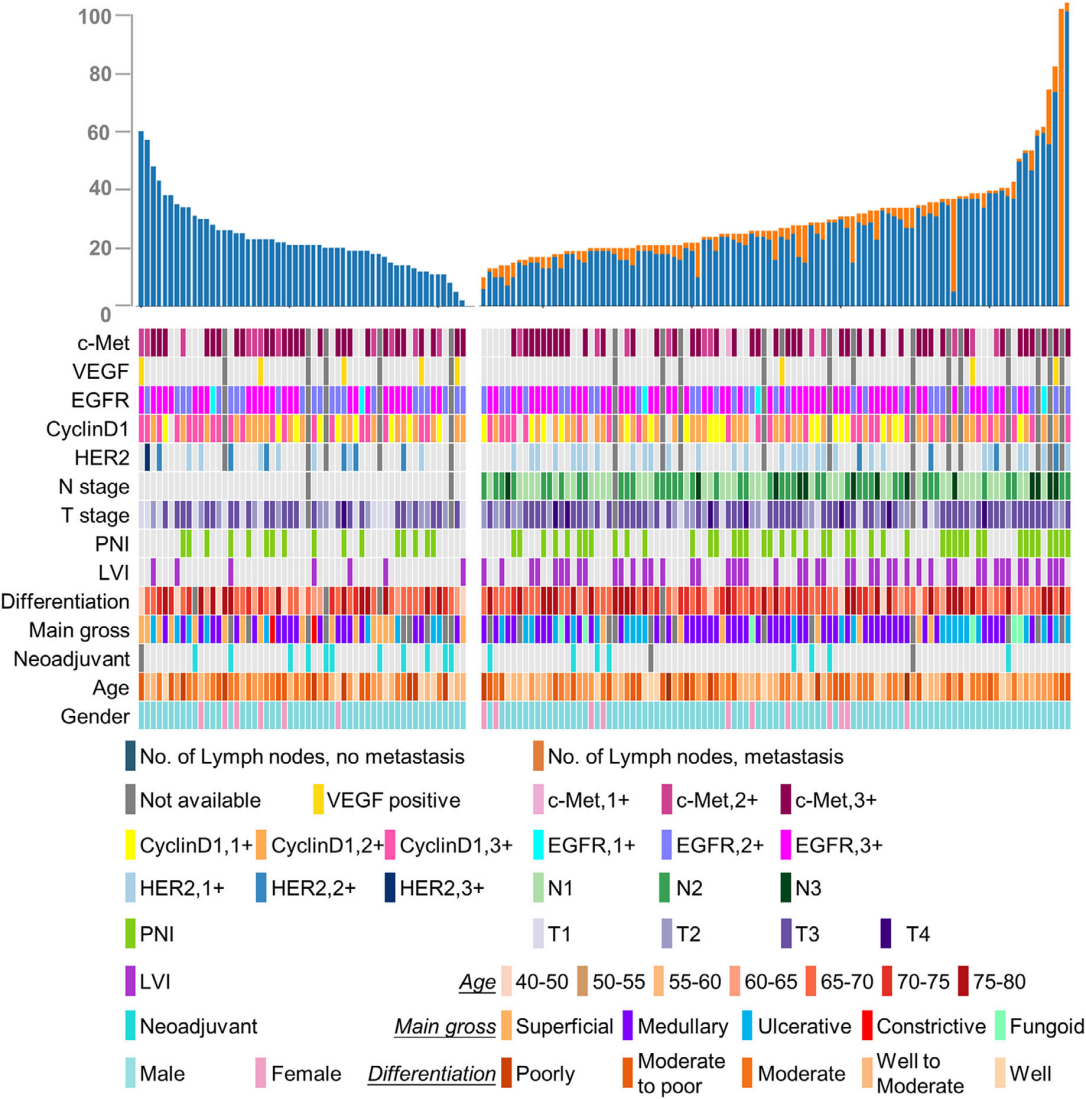
Patients level clinical feature statistics of the test set. The top bar plot shows the number of normal lymph node (in blue) and metastasis lymph node (in orange) of each patient and it is divided into nonmetastasis patients’ group (left) and metastasis

At the patient level, 55 patients had no metastasis (negative group) and 99 patients had metastasis in their surrounding lymph node samples (positive group). The clinical features of each patient in these two groups are shown in Figure 3. The surgical and neoadjuvant details of the patients were shown in Tables S2 and S3. In both groups, there were more male patients than female patients (88 vs 11 in the positive group and 49 vs 6 in the negative group). These two groups were age matched (60.1 ± 7.3; [95% CI, 47.0‐79.0] in the positive group and 60.7 ± 7.9 [95% CI, 46.0‐75.0] in the negative group), and neoadjuvant treatment seemed to helped prevent metastasis (11/55 in the negative group received neoadjuvant therapy, and only 1/99 in the positive group received neoadjuvant therapy). Among these 795 slides, there was a total of 2445 esophageal lymph nodes as follows: 390 with metastasis and 2055 without metastasis based on a manual assessment.

### 3.4 Node‐level assessment of the testing set

**FIGURE 4:**
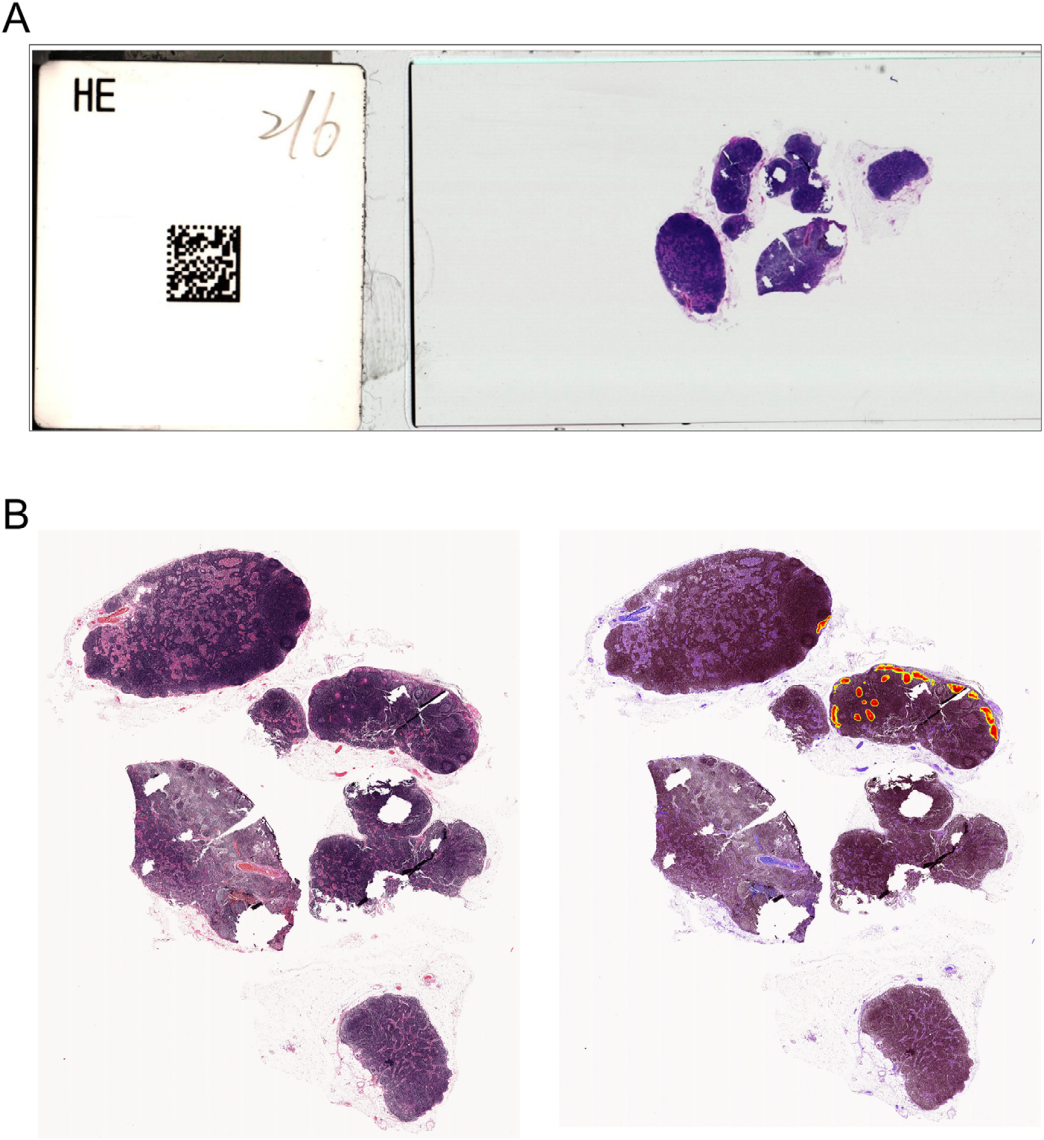
Example of a manual assessment at the node level. A, An image of a glass slide and the tag on the slide clearly show the diagnosis and how many lymph nodes in this slide contain metastasis. B, The WSI (left) and its model prediction result (right). The hot points show that two nodes contain metastasis, which is consistent with the clinical diagnosis on the glass slide

In addition to the slide‐level scoring, the author used lymph node‐level scoring between the pathological diagnosis and predicted metastatic region using the third model (DeepLab v3 with focal loss). As shown in Figure 4, the clinical diagnosis and number of metastatic lymph nodes are provided on the tag of the glass slide. The pathologist could manually assess the correctness of the predicted region and number of predicted metastatic lymph nodes.

### 3.5 Slide‐level metastasis classification of esophageal lymph node WSIs

**FIGURE 5:**
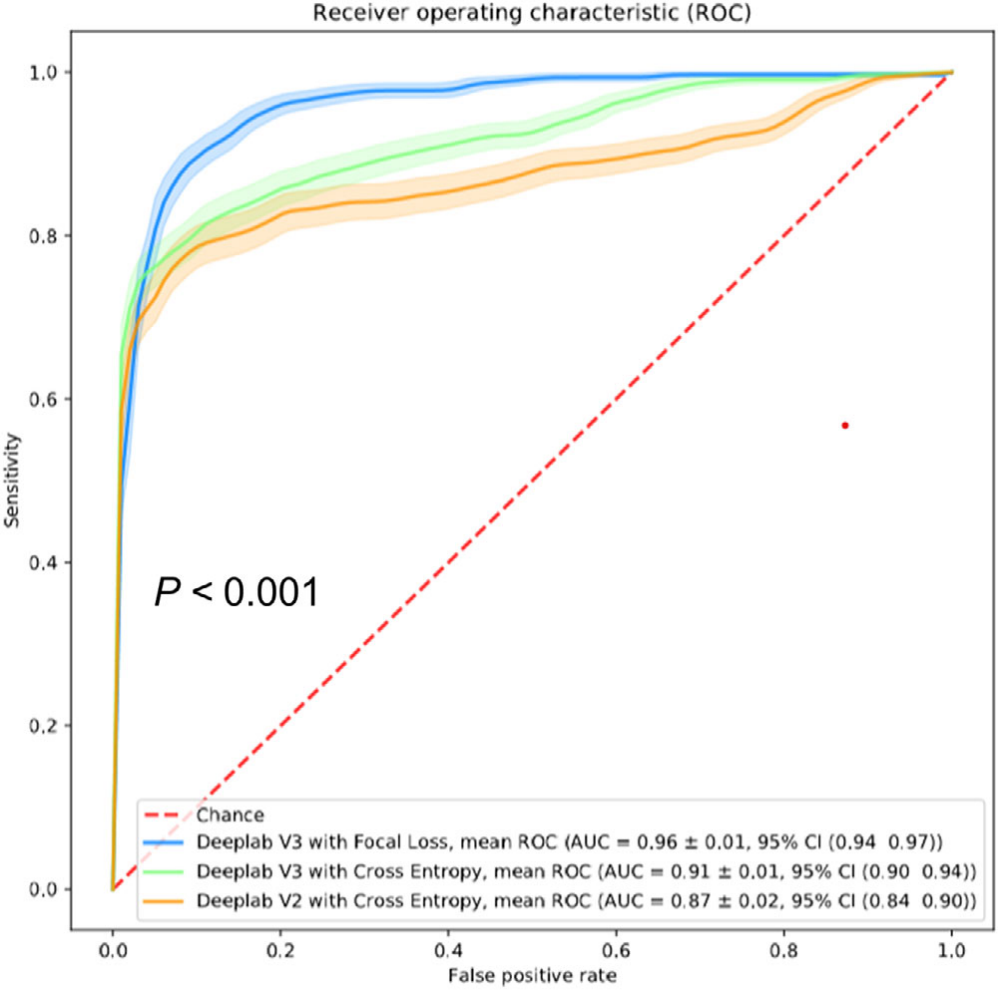
ROC curves with 95% CIs of the three models: DeepLab v2 with cross entropy, DeepLab v3 with cross entropy, and DeepLab v3 with focal loss (*P* *< *.001)

Figure 5 shows the ROC curves of the slide‐level model predictions of the esophageal lymph node WSI test set using the three different models (Table S4). Each model was used to generate a ROC curve with CI using bootstrapping. As shown in Figure 5, the DeepLab v3 model with cross entropy (AUC = 0.91 ± 0.01 [95% CI, 0.90‐0.94]) had a better performance than the DeepLab v2 model with cross entropy (AUC = 0.87 ± 0.02 [95% CI, 0.84‐0.90]), and the DeepLab v3 model with focal loss had a higher AUC (AUC = 0.96 ± 0.01 [95% CI, 0.94‐0.97]) than the model with cross entropy.

Compared to the first two models (DeepLab v2 and DeepLab v3 with cross entropy), the third model (DeepLab v3 with focal loss) exhibited a significant improvement (*P*‐value < .001) using the two‐tailed bootstrap‐based method described by Hanley and McNeil in 1983. Therefore, we use the third model (DeepLab v3 with focal loss) as our proposed model.

**FIGURE 6:**
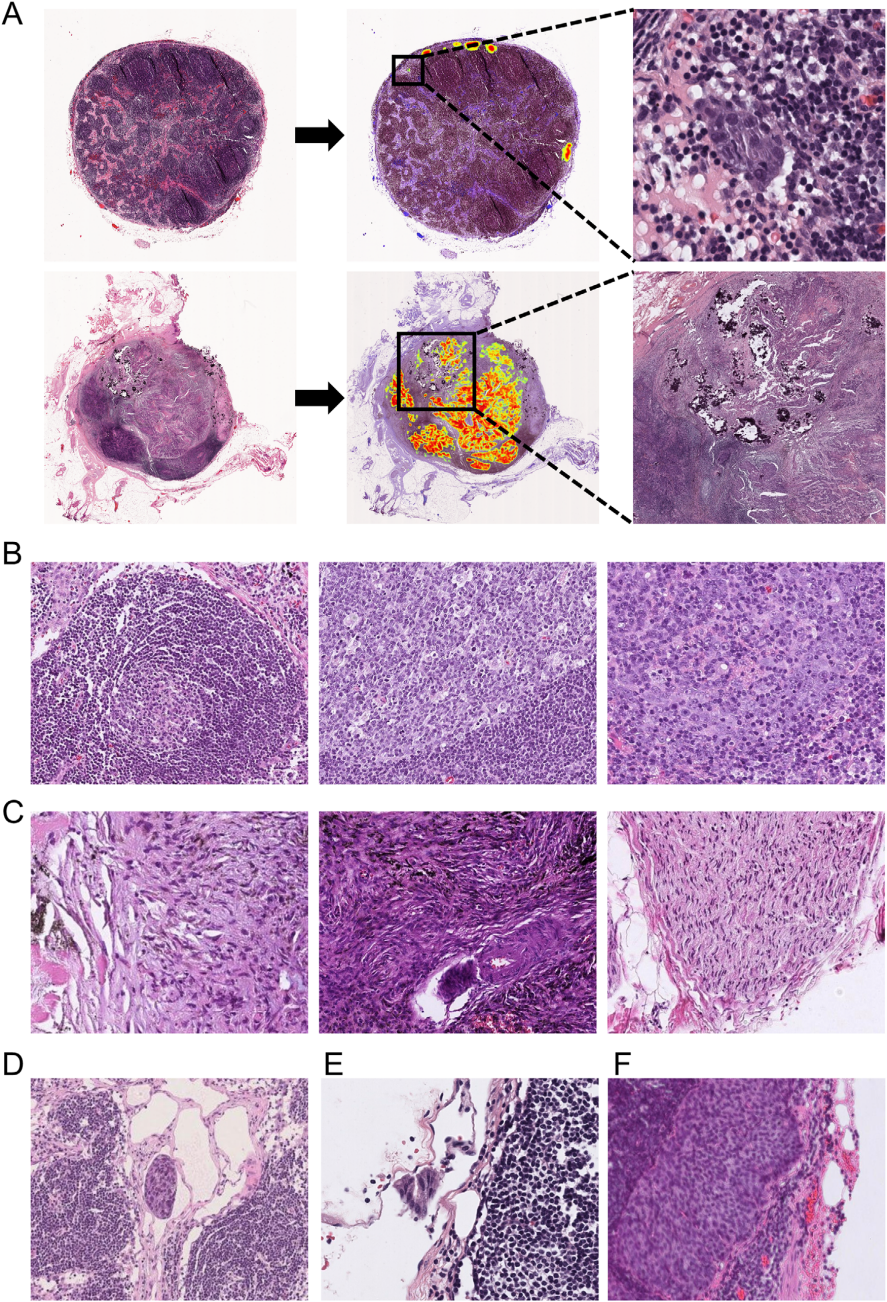
Prediction results for esophageal lymph node WSI test set. A, Model predictions of the third model of both macro‐ and micro‐metastatic regions in esophageal lymph nodes. B and C, False positive cases. D and F, False negative cases

Figure 6A shows the prediction results of the third model of both macro‐ and micro‐metastatic regions in the esophageal lymph nodes.

### 3.6 Node‐level agreement between the model and manual assessment

The performance of the model using the esophageal test set was further analyzed at the lymph node level, and the pathologists examined the prediction accuracy of the model in each lymph node. Among the 2445 esophageal lymph nodes (390 positive nodes and 2055 negative nodes), we successfully detected 387 positive nodes with a sensitivity of 99.2%. In total, 1912/2055 negative nodes were correctly classified, with a specificity of 93.0%. Overall, the node level accuracy was 94.0%.

### 3.7 Model extension

**FIGURE 7:**
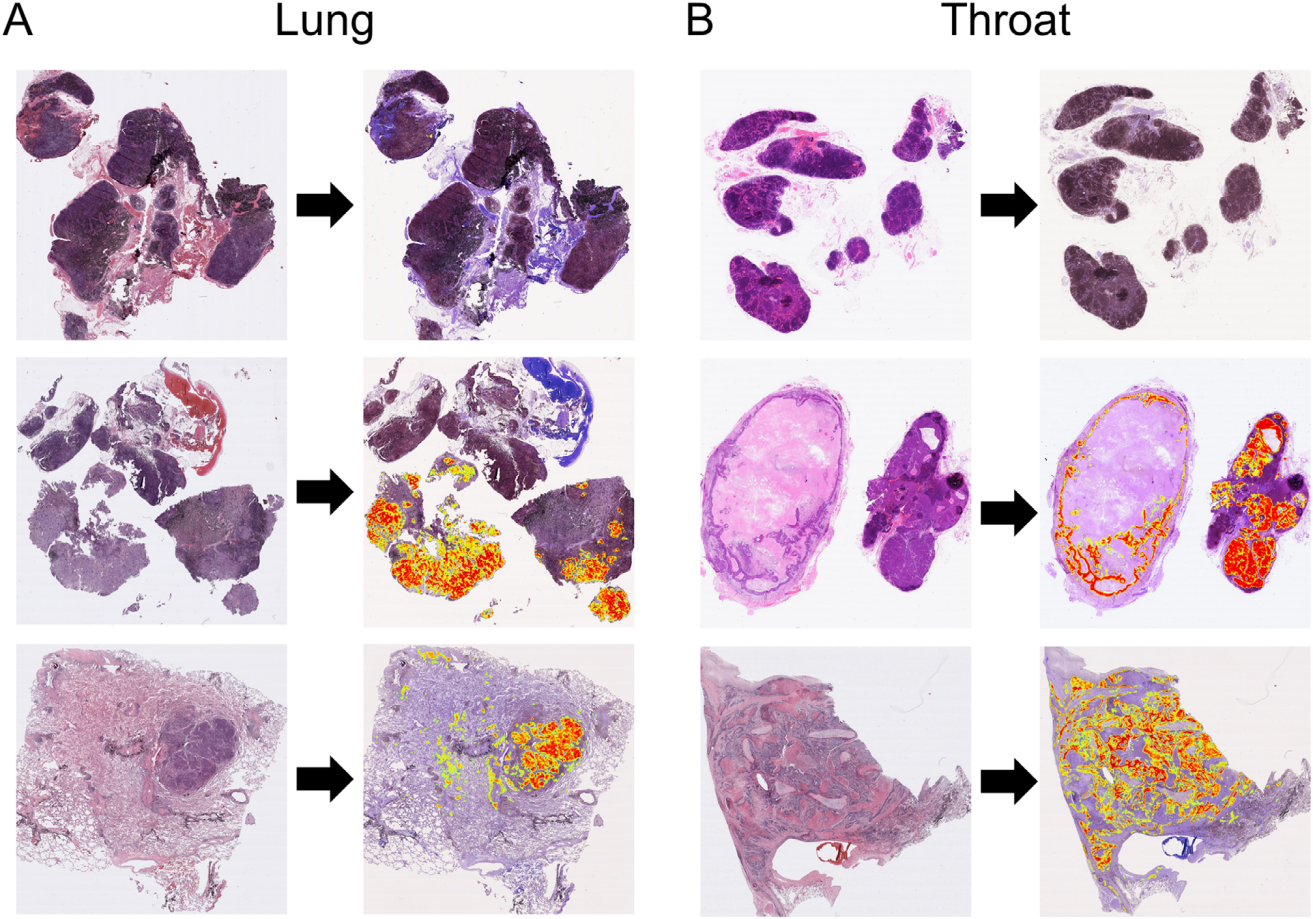
Model expansion. Tumor detection in situ and LNM detection in lung (A) and throat (B) squamous cell carcinoma. Top row: a negative WSI; middle row: a positive lymph node with metastasis; bottom row: a positive tumor in situ

When we applied the proposed model to the lung and throat sets, we found that all squamous cell tumors in these two organs (ie, either squamous cell carcinomas metastasis in the lymph node or the squamous cell carcinoma in situ) had been successfully detected. In the lung set, we obtained a sensitivity of 100% and a specificity of 85.7%. In the throat set, the proposed model achieved a sensitivity of 100% and a specificity of 90.0%. Figure 7 shows the results of the model prediction of negative WSIs and positive lymph node and tumor in situ WSIs in both the lung and throat.

## 4 DISCUSSION

In this study, we developed a deep learning‐based algorithm to localize and differentiate lymph nodes metastasis from ESCCs in China. Squamous cell carcinoma metastasis in esophageal lymph nodes appears more frequently in esophageal cancer patients in China than those in other countries. To the best of our knowledge, this study is the first to develop and validate a deep learning model specific for lymph node metastases of ESCC. Using a large set of manually annotated esophageal lymph node WSIs, we developed a state‐of‐the‐art deep neural network to detect squamous cell carcinoma metastasis. In an independent large‐scale esophageal lymph node test set, our model achieved high performance at both the slide level (AUC = 0.96 ± 0.01 [95% CI, 0.94‐0.97]) and node level (99.2% sensitivity and 93.0% specificity), supporting its potential usage in clinical practice. As shown in the heatmap of tissue images (Figures 4 and 6A), pathologists could use the proposed model to focus on dangerous regions.

Although deep learning is an active research field, its application on histopathology to automatically detect metastasis in esophageal lymph nodes is relatively novel. Most of the published studies focused on clinical features for the detection of esophageal LNM. Dubecz et al^7^ used population clinical data to predict metastasis in esophageal lymph nodes. Several methods for esophageal LNM detection based on radiomic features have been proposed. Ou et al^9^ used radiomic features from contrast‐enhanced CT to predict the esophageal lymph node status. Si et al^10^ proposed combining radiomics features from both CT and fluorodeoxyglucose‐positron emission tomography (FDG‐PET) to train a gradient boosted regression tree model for esophageal LNM detection. However, such clinical or radiomic features fail to reflect direct morphological changes due to metastasis, which can be observed in imaging data from pathology departments (WSIs). Several studies have reported that the automatic detection of LNM showed good performance in breast cancer and other adenocarcinomas. One paper most closely related to our work focused on breast cancer. Ehteshami et al^31^ identified sentinel lymph nodes metastases from breast cancer patients. However, to the best of our knowledge, the use of more detailed pathological information obtained from slides for LNM detection of squamous cell carcinomas has never been explored.

Our model achieves a statistically high performance; however, it is still important to conduct a detailed analysis of failure cases. There were more false positive cases than false negative cases in our large‐scale test set. In general, these false positive cases can be divided into two groups. As shown in Figure 6B, the first group of false positive cases are lymphoblast cells that appear in the germinal center of lymph nodes. These lymphoblast cells are much larger than normal lymph cells and morphologically similar to metastases. Pathologists can distinguish germinal centers from metastases at the macro level, whereas the deep learning model could be used to obtain information only regarding microregions due to the small patch size during the training phase. The second group of false positives contain mainly fibrillar connective tissue (Figure 6C), which is highly curved and irregular. These two characteristics of fibrillar connective tissue also appear in metastatic regions, which may explain why the model often makes mistakes in this type of tissue. There are also nerve fibers (Figure 6C), which can occasionally be seen next to the lymph nodes, and because they were not included in the training set, the model cannot be accurately evaluated.

Compared to false positive cases, it is more important to analyze false negative cases and identify possible solutions to avoid these mistakes. Therefore, we analyzed each of the three false negative cases. In case 1, as shown in Figure 6D, our model missed the micro‐metastasis surrounded by small fibrous lines and empty regions. This surrounding was very rare in the training set, and it is difficult for deep learning models to identify rare cases. Similarly, the second failure was a smaller metastasis located in a lymphatic vessel outside the lymph node (Figure 6E), making it even more difficult to detect correctly. In the third false negative case (Figure 6F), the image was very blurred because the scan was out of focus. This type of failure can be avoided by using a better scanned image.

Although not trained using either the lung or throat, our model still showed good performance in lung and throat lymph nodes metastasis and carcinoma in situ. This result can be explained by the morphological similarity among squamous cell carcinomas in different organs and either lymph nodes or carcinoma in situ. This finding indicates that we can use transfer learning to train new models for organs based on our esophageal metastasis model. This finding also indicates the possibility of obtaining a general metastasis detection model that can predict lymph nodes in any organ with various types of metastasis (such as adenocarcinoma, squamous cell carcinoma, and other tumor types).

The study has some limitations. The model can be further optimized. First, we annotate only metastasis. Because our model makes mistakes on lymph node germinal centers and some connective tissues, we will annotate these types of tissue separately as training set. In addition to this improvement, we will carefully examine the imaging quality, and additional organ testing will be performed in the future to render our model clinically applicable to serve pathologists.

Nevertheless, larger scale studies are needed to further verify the observed impact of digital assistance on efficiency and accuracy, especially in negative cases and actual clinical workflows. Additionally, this study was based on a single center; an external verification research is needed to verify its diagnostic performance and generalizability. Future research also requires forward‐looking and multi‐institutional datasets. The next major challenge is to successfully translate technology into meaningful clinical impact. In the future, this technology needs to be comprehensively evaluated and improved in clinical practice.

In summary, we developed an annotated H&E‐stained esophageal lymph node dataset and trained a deep neural network to automatically detect LNM in squamous cell carcinoma H&E‐stained slides efficiently. Moreover, it is possible to use this model to test lymph nodes metastasis in squamous cell carcinoma from other organs. This study directly demonstrated the potential for determining the localization of squamous cell carcinoma metastases in lymph node and assisting in pathological diagnosis.

## AUTHOR CONTRIBUTIONS

Yi Pan and Zhuo Sun had full access to all data in the study and assume responsibility for the integrity of the data and accuracy of the data analysis. Shuangmei Zou conceptualized and designed the study. Yi Pan, Zhuo Sun, Wenmiao Wang, Zhaoyang Yang, Jia Jia, Xiaolong Feng, Yaxi Wang, Qing Fang, Jiangtao Li, Hongtian Dai, Calvin Ku, Shuhao Wang, and Cancheng Liu helped with acquisition, analysis, and interpretation of data. Yi Pan, Wenmiao Wang, Zhaoyang Yang, Jia Jia, Xiaolong Feng, Yaxi Wang, Qing Fang, Jiangtao Li, Hongtian Dai, and Shuangmei Zou manually annotated the WSIs. Yi Pan, Zhuo Sun, Liyan Xue, Ning Lyu, and Shuangmei Zou drafted of the manuscript. All authors critically revised the manuscript for important intellectual content. Yi Pan, Zhuo Sun, Wenmiao Wang, and Shuangmei Zou helped with statistical analysis. Yi Pan and Shuangmei Zou obtained funding. Calvin Ku, Shuhao Wang, Cancheng Liu, Liyan Xue, Ning Lyu, and Shuangmei Zou provided administrative, technical, and material support. Shuangmei Zou supervised the study.

## ROLE OF THE FUNDER/SPONSOR

The funding sources had no role in the design and conduct of the study; collection, management, analysis, and interpretation of the data; preparation, review, or approval of the manuscript; and decision to submit the manuscript for publication.

## ETHICS APPROVAL AND CONSENT TO PARTICIPATE

The use of human samples was approved by the Cancer Hospital, Chinese Academy of Medical Sciences and Peking Union Medical College, Beijing, China.

## CONFLICT OF INTEREST

The authors declare no conflict of interest.

## Supporting information

SUPPORTING INFORMATION

SUPPORTING INFORMATION

SUPPORTING INFORMATION

SUPPORTING INFORMATION

## Data Availability

The data that supports the findings of this study are available in the supplementary material of this article.
