# Supplementary material for: Automatic detection of squamous cell carcinoma metastasis in esophageal lymph nodes using semantic segmentation
Source: Clin Transl Med. 2020 Jul 28;10(3):e129. doi: 10.1002/ctm2.129 (PMC7418811; doi:10.1002/ctm2.129)
Supplement: Supplementary file 1 — SUPPORTING INFORMATION [file CTM2-10-e129-s001.docx]

**Supplementary Table S1** Pixel and patch level of training data statistics

| **In pixel level** | |
| --- | --- |
| Metastasis | 3,994,633,286 |
| Normal | 28,958,059,178 |
| Ignore | 251,941,136 |
| Overall | 33,204,633,600 |
|  |  |
|  |  |
| **In patch level** | |
| Only Normal | 264,932 |
| Only Metastasis | 17,497 |
| Normal + Metastasis | 26,006 |
| Normal + Ignore | 4,694 |
| Metastasis + Ignore | 9,254 |
| Normal + Metastasis + Ignore | 1,881 |
| Overall | 324,264 |
