# Supplementary material for: Automatic detection of squamous cell carcinoma metastasis in esophageal lymph nodes using semantic segmentation
Source: Clin Transl Med. 2020 Jul 28;10(3):e129. doi: 10.1002/ctm2.129 (PMC7418811; doi:10.1002/ctm2.129)
Supplement: Supplementary file 2 — SUPPORTING INFORMATION [file CTM2-10-e129-s002.docx]

**Supplementary Table S2** Patients’ lymphadenectomy surgical details

| **Variable** | **Conventional thoracotomy** | **Thoracoscopic surgery** |
| --- | --- | --- |
| Number | 141 | 13 |
| Mean age (years) | 60.5±7.4 | 59.6±8.6 |
| Sex |  |  |
| Female | 16 | 1 |
| Male | 125 | 12 |
| Neoadjuvant before operation |  |  |
| Yes | 17 | 2 |
| No | 124 | 11 |
| Thoracic approaches |  |  |
| Left | 94 | 5 |
| Right | 47 | 8 |
| Lymph node dissection modes |  |  |
| One-field | 1 | 1 |
| Two-field | 129 | 10 |
| Three-field | 11 | 2 |
