# Supplementary material for: Automatic detection of squamous cell carcinoma metastasis in esophageal lymph nodes using semantic segmentation
Source: Clin Transl Med. 2020 Jul 28;10(3):e129. doi: 10.1002/ctm2.129 (PMC7418811; doi:10.1002/ctm2.129)
Supplement: Supplementary file 3 — SUPPORTING INFORMATION [file CTM2-10-e129-s003.docx]

**Supplementary Table S3** Patients’ neoadjuvant details before operation

| **Case No.** | **Gender** | **Age (years)** | **Neoadjuvant or not** | **ENI or not** | **Neoadjuvant dose** |
| --- | --- | --- | --- | --- | --- |
| 40 | Male | 47 | Yes | Yes | 95%PGTV 44.94 Gy/2.14Gy/21f; 95%PTV 37.8Gy/1.8Gy/21f |
| 45 | Male | 67 | Yes | Yes | 95%PTV 36.0Gy/1.8Gy/20f; 95%PGTV 42.8Gy/2.14Gy/20f |
| 53 | Male | 66 | Yes | Yes | 95%PGTV 23.54Gy/2.14Gy/11f; 95%PTV 19.8Gy/1.8Gy /11f |
| 55 | Male | 54 | Yes | *N/A* | *N/A* |
| 69 | Male | 55 | Yes | Yes | 95%PTV 36Gy/1.8Gy/20f; 95%PGTV 42.8Gy/2.l 4Gy/20f |
| 71 | Male | 54 | Yes | *N/A* | *N/A* |
| 77 | Male | 54 | Yes | Yes | 95%PGTV 42.8Gy/2.l4Gy /20f; 95%PTV 36Gy/1.8Gy/20f |
| 89 | Male | 56 | Yes | *N/A* | *N/A* |
| 110 | Male | 55 | Yes | *N/A* | *N/A* |
| 111 | Male | 54 | Yes | *N/A* | *N/A* |
| 117 | Male | 66 | Yes | Yes | 95%PGTV 49.22Gy/2.14Gy/23f; 95%PTV 41.4Gy/1.8Gy/23f |
| 118 | Male | 63 | Yes | Yes | 95%PGTV 49.22Gy/2.14Gy /23f; 95 %PTV 41.4Gy /l.8Gy /23f |
| 120 | Male | 48 | Yes | Yes | 95%PTY 60Gy/2Gy/30f; 95% PTVI 60Gy/2Gy/30f |
| 132 | Male | 70 | Yes | Yes | 95%PGTV 55.12Gy/2.12Gy/26f; 95%GTV 55.12Gy/12Gy/26f; 95%PTVI 47.32Gy/1.82Gy/26f |
| 133 | Male | 72 | Yes | Yes | 95%PGTV 49.22Gy/2.l4Gy/23f; 95%PTV 41.4Gy/1.8Gy/23f |
| 144 | Male | 62 | Yes | *N/A* | *N/A* |
| 151 | Male | 64 | Yes | Yes | 95%PGTV 44.94Gy/2.14Gy/21f; 95%PTV 37.8Gy/1.86y/21f |
| 153 | Female | 65 | Yes | Yes | 95 %PGTV 40.28Gy/2.14Gy/20f; 95 %PTV 36Gy/1.8Gy/20f |
| 161 | Male | 69 | Yes | Yes | 95%PTV 36.0Gy/1.8Gy/20f; 95%PGTV 42.8Gy/2.14Gy/20f |

ENI: Elective Nodal Irradiation. Radiation field is marked with preventive elective nodal irradiation in our hospital.

*N/A*: not available. Neoadjuvant therapy of the patient was not done in our hospital, thus the neoadjuvant dose was not available.
