# Supplementary material for: Automatic detection of squamous cell carcinoma metastasis in esophageal lymph nodes using semantic segmentation
Source: Clin Transl Med. 2020 Jul 28;10(3):e129. doi: 10.1002/ctm2.129 (PMC7418811; doi:10.1002/ctm2.129)
Supplement: Supplementary file 4 — SUPPORTING INFORMATION [file CTM2-10-e129-s004.docx]

**Supplementary Table S4** Model predictions of the esophageal lymph node WSI test set using the three different models

| **Slide name** | **File path** | **Diagnosis** | **Score_1** | **Score_2** | **Score_3** |
| --- | --- | --- | --- | --- | --- |
| 1 | /data5/data5/dongzhong/LymphGland/esophagus/2018-07-26/767262-12.kfb | 1 | 0.92218431 | 0.996078431 | 0.995701961 |
| 2 | /data5/data5/dongzhong/LymphGland/esophagus/2018-07-26/726681-13.kfb | 1 | 0.91070196 | 0.996078431 | 0.996078431 |
| 3 | /data5/data5/dongzhong/LymphGland/esophagus/2018-08-03/767200-21.tif | 1 | 0.94344706 | 0.996078431 | 0.994733333 |
| 4 | /data5/data5/dongzhong/LymphGland/esophagus/2018-08-10/720783-13.tif | 1 | 0.90691373 | 0.996078431 | 0.989054902 |
| 5 | /data5/data5/dongzhong/LymphGland/esophagus/2018-08-10/723312-3.tif | 1 | 0.95306667 | 0.996078431 | 0.996078431 |
| 6 | /data5/data5/dongzhong/LymphGland/esophagus/2018-08-10/723325-17.tif | 1 | 0.94001961 | 0.996078431 | 0.996078431 |
| 7 | /data5/data5/dongzhong/LymphGland/esophagus/2018-08-17/723992-41-5;5.tif | 1 | 0.95662745 | 0.996078431 | 0.996078431 |
| 8 | /data5/data5/dongzhong/LymphGland/esophagus/2018-08-17/724177-14-1;1.tif | 1 | 0.86011373 | 0.992890196 | 0.996078431 |
| 9 | /data5/data5/dongzhong/LymphGland/esophagus/2018-08-17/736608-13-1;1.tif | 1 | 0.94885882 | 0.996078431 | 0.996078431 |
| 10 | /data5/data5/dongzhong/LymphGland/esophagus/2018-08-17/738868-19-4;5.tif | 1 | 0.90115686 | 0.996078431 | 0.988007843 |
| 11 | /data5/data5/dongzhong/LymphGland/esophagus/2018-08-17/743168-17-1;5.tif | 1 | 0.89518824 | 0.993156863 | 0.980611765 |
| 12 | /data5/data5/dongzhong/LymphGland/esophagus/2018-08-17/750621-19-1;3.tif | 1 | 0.95236078 | 0.996078431 | 0.996078431 |
| 13 | /data5/data5/dongzhong/LymphGland/esophagus/2018-08-17/751696-25-1;1.tif | 1 | 0.96118039 | 0.996078431 | 0.996078431 |
| 14 | /data5/data5/dongzhong/LymphGland/esophagus/2018-08-17/747855-31-2;2.tif | 1 | 0.95696078 | 0.996078431 | 0.99327451 |
| 15 | /data5/data5/dongzhong/LymphGland/esophagus/2018-08-17/763565-11-2;5.tif | 1 | 0.88247451 | 0.990917647 | 0.991741176 |
| 16 | /data5/data5/dongzhong/LymphGland/esophagus/2018-08-24/714556-26-1;1.tif | 1 | 0.93746275 | 0.996078431 | 0.996078431 |
| 17 | /data5/data5/dongzhong/LymphGland/esophagus/2018-08-24/719365-22-1;2.tif | 1 | 0.92752157 | 0.996078431 | 0.956560784 |
| 18 | /data5/data5/dongzhong/LymphGland/esophagus/2018-08-24/710821-15-1;1.tif | 1 | 0.95132549 | 0.996078431 | 0.996078431 |
| 19 | /data5/data5/dongzhong/LymphGland/esophagus/2018-08-24/705235-11-3;6.tif | 1 | 0.94436078 | 0.996078431 | 0.996078431 |
| 20 | /data5/data5/dongzhong/LymphGland/esophagus/2018-08-24/706626-15-1;2.tif | 1 | 0.95245882 | 0.996078431 | 0.996078431 |
| 21 | /data5/data5/dongzhong/LymphGland/esophagus/2018-08-24/707164-11-1;1.tif | 1 | 0.92710196 | 0.996078431 | 0.996078431 |
| 22 | /data5/data5/dongzhong/LymphGland/esophagus/2018-08-24/708454-17-2;8.tif | 1 | 0.94909412 | 0.996078431 | 0.996078431 |
| 23 | /data5/data5/dongzhong/LymphGland/esophagus/2018-08-24/713984-17-1;6.tif | 1 | 0.96567843 | 0.996078431 | 0.996078431 |
| 24 | /data5/data5/dongzhong/LymphGland/esophagus/2018-08-24/708900-11-2;5.tif | 1 | 0.93167059 | 0.996078431 | 0.996078431 |
| 25 | /data5/data5/dongzhong/LymphGland/esophagus/2018-08-24/707307-32-1;1.tif | 1 | 0.98714902 | 0.996078431 | 0.996078431 |
| 26 | /data5/data5/dongzhong/LymphGland/esophagus/2018-08-24/709988-13-2;3.tif | 1 | 0.92724314 | 0.996078431 | 0.985909804 |
| 27 | /data5/data5/dongzhong/LymphGland/esophagus/2018-08-24/705537-17-1;4.tif | 1 | 0.94051765 | 0.996078431 | 0.996078431 |
| 28 | /data5/data5/dongzhong/LymphGland/esophagus/2018-08-24/708896-12-1;4.tif | 1 | 0.9188 | 0.996078431 | 0.996078431 |
| 29 | /data5/data5/dongzhong/LymphGland/esophagus/2018-08-24/713045-16-1;5.tif | 1 | 0.95846275 | 0.996078431 | 0.996078431 |
| 30 | /data5/data5/dongzhong/LymphGland/esophagus/2018-08-24/709081-14-1;3.tif | 1 | 0.91837255 | 0.996078431 | 0.996078431 |
| 31 | /data5/data5/dongzhong/LymphGland/esophagus/2018-11-09/789260-12-1;1.ndpi | 1 | 0.93372157 | 0.996078431 | 0.988341176 |
| 32 | /data5/data5/dongzhong/LymphGland/esophagus/2018-11-09/789261-22-1;4.ndpi | 1 | 0.80372941 | 0.044133333 | 0.007501961 |
| 33 | /data5/data5/dongzhong/LymphGland/esophagus/2018-11-02/789931-13-1;3.ndpi | 1 | 0.73076863 | 0.010964706 | 0.000619608 |
| 34 | /data5/data5/dongzhong/LymphGland/esophagus/2018-11-02/790210-9-1;3.ndpi | 1 | 0.90593333 | 0.656831373 | 0.234078431 |
| 35 | /data5/data5/dongzhong/LymphGland/esophagus/2018-11-02/781405-22-2;5.ndpi | 1 | 0.91441569 | 0.944129412 | 0.494462745 |
| 36 | /data5/data5/dongzhong/LymphGland/esophagus/2018-11-02/781530-12-1;1.ndpi | 1 | 0.86411373 | 0.229772549 | 0.004431373 |
| 37 | /data5/data5/dongzhong/LymphGland/esophagus/2018-12-28/2018-12-21_13.04.37.ndpi | 1 | 0.63440784 | 0.067815686 | 0.686976471 |
| 38 | /data5/data5/dongzhong/LymphGland/esophagus/2018-12-28/2018-12-27_16.01.24.ndpi | 1 | 0.93863137 | 0.996078431 | 0.996078431 |
| 39 | /data5/data5/dongzhong/LymphGland/esophagus/2018-11-02/783372-16-1;1.ndpi | 1 | 0.93145098 | 0.741019608 | 0.548160784 |
| 40 | /data5/data5/dongzhong/LymphGland/esophagus/2018-12-28/2018-12-27_16.08.26.ndpi | 1 | 0.96103922 | 0.996078431 | 0.996078431 |
| 41 | /data5/data5/dongzhong/LymphGland/esophagus/2018-11-02/785318-11-2;5.ndpi | 1 | 0.81692941 | 0.02134902 | 0.001862745 |
| 42 | /data5/data5/dongzhong/LymphGland/esophagus/2018-11-02/785912-16-1;5.ndpi | 1 | 0.8629098 | 0.000113725 | 0.001972549 |
| 43 | /data5/data5/dongzhong/LymphGland/esophagus/2018-11-02/786098-8-2;4.ndpi | 1 | 0.88860392 | 0.893556863 | 0.021478431 |
| 44 | /data5/data5/dongzhong/LymphGland/esophagus/2018-11-02/786457-17-1;5.ndpi | 1 | 0.91648235 | 0.931521569 | 0.261368627 |
| 45 | /data5/data5/dongzhong/LymphGland/esophagus/2018-11-02/787239-22-1;2.ndpi | 1 | 0.92743529 | 0.981635294 | 0.096984314 |
| 46 | /data5/data5/dongzhong/LymphGland/esophagus/2018-11-02/787941-13-2;3.ndpi | 1 | 0.73820392 | 0.043858824 | 0.006329412 |
| 47 | /data5/data5/dongzhong/LymphGland/esophagus/2018-11-09/787945-15-1;4.ndpi | 1 | 0.92876078 | 0.994713725 | 0.901870588 |
| 48 | /data5/data5/dongzhong/LymphGland/esophagus/2018-12-20/790564-13-1;1.ndpi | 1 | 0.91547843 | 0.995345098 | 0.991392157 |
| 49 | /data5/data5/dongzhong/LymphGland/esophagus/2018-12-20/790573-22-1;2.ndpi | 1 | 0.77073333 | 0.926772549 | 0.45025098 |
| 50 | /data5/data5/dongzhong/LymphGland/esophagus/2018-11-02/791683-10-1;2.ndpi | 1 | 0.8198549 | 0.067235294 | 5.09804E-05 |
| 51 | /data5/data5/dongzhong/LymphGland/esophagus/2018-12-20/792225-7-1;1.ndpi | 1 | 0.95410588 | 0.99252549 | 0.996078431 |
| 52 | /data5/data5/dongzhong/LymphGland/esophagus/2018-12-20/793167-18-2;3.ndpi | 1 | 0.91271765 | 0.98054902 | 0.985282353 |
| 53 | /data5/data5/dongzhong/LymphGland/esophagus/2018-12-20/793533-23-1;1.ndpi | 1 | 0.91472549 | 0.964772549 | 0.991929412 |
| 54 | /data5/data5/dongzhong/LymphGland/esophagus/2018-12-20/793834-19-0;3.ndpi | 1 | 0.90112941 | 0.904713725 | 0.960333333 |
| 55 | /data5/data5/dongzhong/LymphGland/esophagus/2018-12-20/794196-11-1;6.ndpi | 1 | 0.94762745 | 0.996078431 | 0.996078431 |
| 56 | /data5/data5/dongzhong/LymphGland/esophagus/2018-12-20/794889-16-3;3.ndpi | 1 | 0.93312941 | 0.988023529 | 0.996078431 |
| 57 | /data5/data5/dongzhong/LymphGland/esophagus/2018-11-02/795996-12-1;3.ndpi | 1 | 0.90999216 | 0.996078431 | 0.875615686 |
| 58 | /data5/data5/dongzhong/LymphGland/esophagus/2018-12-20/796186-15-1;5.ndpi | 1 | 0.85558039 | 0.97507451 | 0.893952941 |
| 59 | /data5/data5/dongzhong/LymphGland/esophagus/2018-12-20/797701-16-1;6.ndpi | 1 | 0.94216863 | 0.996078431 | 0.996078431 |
| 60 | /data5/data5/dongzhong/LymphGland/esophagus/2018-11-09/798305-11-1;3.ndpi | 1 | 0.86550196 | 0.037388235 | 0.981713725 |
| 61 | /data5/data5/dongzhong/LymphGland/esophagus/2018-12-20/798347-19-1；3.ndpi | 1 | 0.92023137 | 0.98694902 | 0.9884 |
| 62 | /data5/data5/dongzhong/LymphGland/esophagus/2018-11-09/804579-13-1;4.ndpi | 1 | 0.66748627 | 0.0058 | 0 |
| 63 | /data5/data5/dongzhong/LymphGland/esophagus/2018-11-09/805750-22-1;2.ndpi | 1 | 0.86691765 | 0.945215686 | 0.948113725 |
| 64 | /data5/data5/dongzhong/LymphGland/esophagus/2018-11-09/775705-16-1;1.ndpi | 1 | 0.75366667 | 0.019745098 | 3.92157E-05 |
| 65 | /data5/data5/dongzhong/LymphGland/esophagus/2018-11-09/775522-21-1;4.ndpi | 1 | 0.92281176 | 0.948960784 | 0.063196078 |
| 66 | /data5/data5/dongzhong/LymphGland/esophagus/2018-11-09/773527-23-1;1.ndpi | 1 | 0.9326 | 0.16605098 | 0.177466667 |
| 67 | /data5/data5/dongzhong/LymphGland/esophagus/2018-11-09/773083-17-2;5.ndpi | 1 | 0.93007059 | 0.97274902 | 0.002113725 |
| 68 | /data5/data5/dongzhong/LymphGland/esophagus/2018-11-09/771977-12-1;5.ndpi | 1 | 0.87439608 | 0.703909804 | 0.000145098 |
| 69 | /data5/data5/dongzhong/LymphGland/esophagus/2018-11-09/769058-17-4;4.ndpi | 1 | 0.92693725 | 0.96492549 | 0.267411765 |
| 70 | /data5/data5/dongzhong/LymphGland/esophagus/2018-11-09/768903-9-1;4.ndpi | 1 | 0.86885098 | 0.04827451 | 0.008027451 |
| 71 | /data5/data5/dongzhong/LymphGland/esophagus/2018-11-09/768724-11-1;3.ndpi | 1 | 0.92173725 | 0.966333333 | 0.047980392 |
| 72 | /data5/data5/dongzhong/LymphGland/esophagus/2018-11-02/768709-18-1;1.ndpi | 1 | 0.88476078 | 0.058215686 | 0.068423529 |
| 73 | /data5/data5/dongzhong/LymphGland/esophagus/2018-11-02/784527-23-1;5.ndpi | 1 | 0.95415686 | 0.996078431 | 0.9768 |
| 74 | /data5/data5/dongzhong/LymphGland/esophagus/2018-11-02/783685-18-1;6.ndpi | 1 | 0.94989412 | 0.990956863 | 0.716890196 |
| 75 | /data5/data5/dongzhong/LymphGland/esophagus/2018-11-02/776561-17-1;7.ndpi | 1 | 0.94647451 | 0.992819608 | 0.598333333 |
| 76 | /data5/data5/dongzhong/LymphGland/esophagus/2018-11-02/776703-30-1;2.ndpi | 1 | 0.94523922 | 0.991556863 | 0.996078431 |
| 77 | /data5/data5/dongzhong/LymphGland/esophagus/2018-11-02/777581-12-1;1.ndpi | 1 | 0.92935686 | 0.761611765 | 0.976552941 |
| 78 | /data5/data5/dongzhong/LymphGland/esophagus/2018-11-02/778514-16-1;1.ndpi | 1 | 0.84857647 | 0.198996078 | 0.004101961 |
| 79 | /data5/data5/dongzhong/LymphGland/esophagus/2019-02-03/2018-12-27 19.01.30.ndpi | 1 | 0.86396471 | 0.856129412 | 0.785956863 |
| 80 | /data5/data5/dongzhong/LymphGland/esophagus/2019-02-03/2018-12-27 19.23.48.ndpi | 1 | 0.60955686 | 0.14614902 | 0.835094118 |
| 81 | /data5/data5/dongzhong/LymphGland/esophagus/2019-02-03/2018-12-27 19.42.53.ndpi | 1 | 0.89777647 | 0.797576471 | 0.980294118 |
| 82 | /data5/data5/dongzhong/LymphGland/esophagus/2019-02-03/800400-32-32 - 2019-01-11 10.55.04.ndpi | 1 | 0.88058431 | 0.977435294 | 0.924737255 |
| 83 | /data5/data5/dongzhong/LymphGland/esophagus/2019-02-03/800645-14-14 - 2019-01-11 11.08.17.ndpi | 1 | 0.84447451 | 0.895737255 | 0.950352941 |
| 84 | /data5/data5/dongzhong/LymphGland/esophagus/2019-02-03/801625-9-9 - 2019-01-11 10.33.38.ndpi | 1 | 0.58764706 | 0.555101961 | 0.127980392 |
| 85 | /data5/data5/dongzhong/LymphGland/esophagus/2019-02-03/801768-1-1 - 2019-01-11 11.47.37.ndpi | 1 | 0.16612157 | 7.84314E-06 | 0.000176471 |
| 86 | /data5/data5/dongzhong/LymphGland/esophagus/2019-02-03/801804-17-17 - 2019-01-11 14.48.40.ndpi | 1 | 0.89241569 | 0.991717647 | 0.996078431 |
| 87 | /data5/data5/dongzhong/LymphGland/esophagus/2019-02-03/802277-12-12 - 2019-01-11 12.09.08.ndpi | 1 | 0.90403922 | 0.95452549 | 0.912203922 |
| 88 | /data5/data5/dongzhong/LymphGland/esophagus/2019-02-03/802465-14-14 - 2019-01-11 14.55.46.ndpi | 1 | 0.78647059 | 0.841352941 | 0.50972549 |
| 89 | /data5/data5/dongzhong/LymphGland/esophagus/2019-02-03/802624-10-10 - 2019-01-11 12.46.29.ndpi | 1 | 0.64285882 | 0.575988235 | 0.099733333 |
| 90 | /data5/data5/dongzhong/LymphGland/esophagus/2019-02-03/802630-32-32 - 2019-01-11 15.19.46.ndpi | 1 | 0.89626667 | 0.989858824 | 0.987713725 |
| 91 | /data5/data5/dongzhong/LymphGland/esophagus/2019-02-03/803307-14-14 - 2019-01-11 13.35.12.ndpi | 1 | 0.8079098 | 0.782168627 | 0.63505098 |
| 92 | /data5/data5/dongzhong/LymphGland/esophagus/2019-02-03/803626-12-12 - 2019-01-11 14.29.35.ndpi | 1 | 0.90953333 | 0.704792157 | 0.994721569 |
| 93 | /data6/医科院肿瘤医院正常和其他/2018-11-09/淋巴（食管）正常/805556-16-1;1.ndpi | 1 | 0.78559608 | 0.081388235 | 2.35294E-05 |
| 94 | /data5/data5/dongzhong/LymphGland/esophagus/2018-08-17/723992-30-8;8.tif | 1 | 0.95531765 | 0.996078431 | 0.99365098 |
| 95 | /data5/data5/dongzhong/LymphGland/esophagus/2018-08-17/736608-14-1;1.tif | 1 | 0.93558039 | 0.996078431 | 0.993568627 |
| 96 | /data5/data5/dongzhong/LymphGland/esophagus/2018-08-17/738868-14-1;3.tif | 1 | 0.48672157 | 0.504556863 | 0.545094118 |
| 97 | /data5/data5/dongzhong/LymphGland/esophagus/2018-08-17/750621-13-2;3.tif | 1 | 0.92673725 | 0.996078431 | 0.996078431 |
| 98 | /data5/data5/dongzhong/LymphGland/esophagus/2018-08-17/747855-33-1;4.tif | 1 | 0.92527843 | 0.996078431 | 0.974247059 |
| 99 | /data5/data5/dongzhong/LymphGland/esophagus/2018-08-24/714556-25-3;3.tif | 1 | 0.92443529 | 0.996078431 | 0.995658824 |
| 100 | /data5/data5/dongzhong/LymphGland/esophagus/2018-08-24/719365-13-1;5.tif | 1 | 0.69983137 | 0.991964706 | 0.693917647 |
| 101 | /data5/data5/dongzhong/LymphGland/esophagus/2018-08-24/710821-12-1;1.tif | 1 | 0.95121176 | 0.996078431 | 0.996078431 |
| 102 | /data5/data5/dongzhong/LymphGland/esophagus/2018-08-24/705235-12-3;6.tif | 1 | 0.96639608 | 0.996078431 | 0.996078431 |
| 103 | /data5/data5/dongzhong/LymphGland/esophagus/2018-08-24/707164-20-1;1.tif | 1 | 0.96417647 | 0.996078431 | 0.996078431 |
| 104 | /data5/data5/dongzhong/LymphGland/esophagus/2018-08-24/708454-19-1;1.tif | 1 | 0.95778431 | 0.996078431 | 0.996078431 |
| 105 | /data5/data5/dongzhong/LymphGland/esophagus/2018-08-24/705537-16-1;1.tif | 1 | 0.93174118 | 0.996078431 | 0.996078431 |
| 106 | /data5/data5/dongzhong/LymphGland/esophagus/2018-08-24/708896-19-1;2.tif | 1 | 0.95020784 | 0.996078431 | 0.992843137 |
| 107 | /data5/data5/dongzhong/LymphGland/esophagus/2018-11-09/789260-21-1;1.ndpi | 1 | 0.73179608 | 0.447356863 | 0.011501961 |
| 108 | /data5/data5/dongzhong/LymphGland/esophagus/2018-11-02/789931-10-1;1.ndpi | 1 | 0.91311765 | 0.970094118 | 0.654917647 |
| 109 | /data5/data5/dongzhong/LymphGland/esophagus/2018-12-28/2018-12-21_12.30.57.ndpi | 1 | 0.91052941 | 0.996078431 | 0.995996078 |
| 110 | /data5/data5/dongzhong/LymphGland/esophagus/2018-11-02/782266-11-2;2.ndpi | 1 | 0.93153725 | 0.444733333 | 0.447584314 |
| 111 | /data5/data5/dongzhong/LymphGland/esophagus/2018-12-28/2018-12-27_15.59.34.ndpi | 1 | 0.90048627 | 0.994768627 | 0.985180392 |
| 112 | /data5/data5/dongzhong/LymphGland/esophagus/2018-11-02/783372-21-1;2.ndpi | 1 | 0.94487059 | 0.987109804 | 0.993776471 |
| 113 | /data5/data5/dongzhong/LymphGland/esophagus/2018-12-28/2018-12-27_16.07.30.ndpi | 1 | 0.30583137 | 0.345839216 | 0.062007843 |
| 114 | /data5/data5/dongzhong/LymphGland/esophagus/2018-11-02/785318-9-1;3.ndpi | 1 | 0.86276078 | 0.038545098 | 0.003062745 |
| 115 | /data5/data5/dongzhong/LymphGland/esophagus/2018-12-20/790573-13-1;1.ndpi | 1 | 0.89921176 | 0.986290196 | 0.97905098 |
| 116 | /data5/data5/dongzhong/LymphGland/esophagus/2018-12-20/793533-20-3;3.ndpi | 1 | 0.86374902 | 0.778415686 | 0.167439216 |
| 117 | /data5/data5/dongzhong/LymphGland/esophagus/2018-11-02/795996-11-1;1.ndpi | 1 | 0.9409451 | 0.951168627 | 0.67592549 |
| 118 | /data5/data5/dongzhong/LymphGland/esophagus/2018-12-20/797701-12-2;5.ndpi | 1 | 0.91356863 | 0.993360784 | 0.995521569 |
| 119 | /data5/data5/dongzhong/LymphGland/esophagus/2018-12-20/798347-14-1；1.ndpi | 1 | 0.93988235 | 0.975913725 | 0.996078431 |
| 120 | /data5/data5/dongzhong/LymphGland/esophagus/2018-11-09/775522-20-1;1.ndpi | 1 | 0.91571765 | 0.538894118 | 0.006764706 |
| 121 | /data5/data5/dongzhong/LymphGland/esophagus/2018-11-09/773527-12-1;1.ndpi | 1 | 0.90914902 | 0.637709804 | 0.116105882 |
| 122 | /data5/data5/dongzhong/LymphGland/esophagus/2018-11-09/771977-13-2;3.ndpi | 1 | 0.93632941 | 0.91885098 | 0.187894118 |
| 123 | /data5/data5/dongzhong/LymphGland/esophagus/2018-11-09/768709-14-2;4.ndpi | 1 | 0.85838824 | 0.904517647 | 0.070843137 |
| 124 | /data5/data5/dongzhong/LymphGland/esophagus/2018-11-02/778514-12-1;1.ndpi | 1 | 0.83935294 | 0.372694118 | 2.35294E-05 |
| 125 | /data5/data5/dongzhong/LymphGland/esophagus/2019-02-03/2018-12-27 19.25.29.ndpi | 1 | 0.77138039 | 0.251070588 | 0.899972549 |
| 126 | /data5/data5/dongzhong/LymphGland/esophagus/2019-02-03/800400-34-34 - 2019-01-11 10.58.09.ndpi | 1 | 0.91815294 | 0.995039216 | 0.995537255 |
| 127 | /data5/data5/dongzhong/LymphGland/esophagus/2019-02-03/800645-13-13 - 2019-01-11 11.06.28.ndpi | 1 | 0.92293725 | 0.996078431 | 0.996078431 |
| 128 | /data5/data5/dongzhong/LymphGland/esophagus/2019-02-03/802630-20-20 - 2019-01-11 12.54.53.ndpi | 1 | 0.92205098 | 0.989764706 | 0.991341176 |
| 129 | /data5/data5/dongzhong/LymphGland/esophagus/2018-08-17/724177-23-1;3.tif | 1 | 0.90801961 | 0.996078431 | 0.983933333 |
| 130 | /data5/data5/dongzhong/LymphGland/esophagus/2018-08-17/751696-26-1;6.tif | 1 | 0.91116863 | 0.996078431 | 0.987113725 |
| 131 | /data5/data5/dongzhong/LymphGland/esophagus/2018-08-17/747855-24-2;2.tif | 1 | 0.94574902 | 0.996078431 | 0.99565098 |
| 132 | /data5/data5/dongzhong/LymphGland/esophagus/2018-08-24/705235-13-2;5.tif | 1 | 0.96224314 | 0.996078431 | 0.996078431 |
| 133 | /data5/data5/dongzhong/LymphGland/esophagus/2018-08-24/707164-19-1;1.tif | 1 | 0.96247059 | 0.996078431 | 0.996078431 |
| 134 | /data5/data5/dongzhong/LymphGland/esophagus/2018-08-24/708454-7-1;1.tif | 1 | 0.9529451 | 0.996078431 | 0.996078431 |
| 135 | /data5/data5/dongzhong/LymphGland/esophagus/2018-08-24/708900-7-1;4.tif | 1 | 0.88847059 | 0.993545098 | 0.986839216 |
| 136 | /data5/data5/dongzhong/LymphGland/esophagus/2018-11-02/789931-16-2;6.ndpi | 1 | 0.88337647 | 0.310305882 | 0.086501961 |
| 137 | /data5/data5/dongzhong/LymphGland/esophagus/2018-11-02/783372-20-1;1.ndpi | 1 | 0.95396863 | 0.995423529 | 0.969854902 |
| 138 | /data5/data5/dongzhong/LymphGland/esophagus/2018-11-02/783371-15-1;1.ndpi | 1 | 0.88809412 | 0.898631373 | 0.967498039 |
| 139 | /data5/data5/dongzhong/LymphGland/esophagus/2018-11-02/785318-12-2;3.ndpi | 1 | 0.86353333 | 0.048980392 | 0.026619608 |
| 140 | /data5/data5/dongzhong/LymphGland/esophagus/2018-12-20/792225-9-1;4.ndpi | 1 | 0.92481176 | 0.971941176 | 0.820576471 |
| 141 | /data5/data5/dongzhong/LymphGland/esophagus/2018-12-20/793533-14-6;6.ndpi | 1 | 0.90550588 | 0.930211765 | 0.996078431 |
| 142 | /data5/data5/dongzhong/LymphGland/esophagus/2018-12-20/794196-10-2;4.ndpi | 1 | 0.93981961 | 0.994733333 | 0.996078431 |
| 143 | /data5/data5/dongzhong/LymphGland/esophagus/2018-12-20/794889-17-1;2.ndpi | 1 | 0.93139608 | 0.992956863 | 0.99565098 |
| 144 | /data5/data5/dongzhong/LymphGland/esophagus/2018-12-20/795996-8-1;5.ndpi | 1 | 0.94318431 | 0.993129412 | 0.996078431 |
| 145 | /data5/data5/dongzhong/LymphGland/esophagus/2018-12-20/797701-14-2;5.ndpi | 1 | 0.93892157 | 0.996078431 | 0.996078431 |
| 146 | /data5/data5/dongzhong/LymphGland/esophagus/2018-11-09/804579-14-1;5.ndpi | 1 | 0.93526275 | 0.208411765 | 0.00032549 |
| 147 | /data5/data5/dongzhong/LymphGland/esophagus/2018-11-02/776703-31-2;3.ndpi | 1 | 0.91857255 | 0.47865098 | 0.234313725 |
| 148 | /data5/data5/dongzhong/LymphGland/esophagus/2018-11-02/777581-4-1;1.ndpi | 1 | 0.91402353 | 0.11745098 | 0.030937255 |
| 149 | /data5/data5/dongzhong/LymphGland/esophagus/2019-02-03/2018-12-27 18.57.47.ndpi | 1 | 0.8545098 | 0.671372549 | 0.036858824 |
| 150 | /data5/data5/dongzhong/LymphGland/esophagus/2019-02-03/2018-12-27 19.44.22.ndpi | 1 | 0.9024549 | 0.959137255 | 0.99025098 |
| 151 | /data5/data5/dongzhong/LymphGland/esophagus/2019-02-03/800645-15-15 - 2019-01-11 11.10.34.ndpi | 1 | 0.91601961 | 0.996078431 | 0.996078431 |
| 152 | /data5/data5/dongzhong/LymphGland/esophagus/2019-02-03/801804-18-18 - 2019-01-11 14.50.03.ndpi | 1 | 0.87742745 | 0.674541176 | 0.700458824 |
| 153 | /data5/data5/dongzhong/LymphGland/esophagus/2018-08-17/724177-4-1;1.tif | 1 | 0.85923529 | 0.985458824 | 0.991180392 |
| 154 | /data5/data5/dongzhong/LymphGland/esophagus/2018-08-17/738868-20-2;6.tif | 1 | 0.67431765 | 0.830011765 | 0.581145098 |
| 155 | /data5/data5/dongzhong/LymphGland/esophagus/2018-08-17/743168-12-2;2.tif | 1 | 0.92242745 | 0.996078431 | 0.993592157 |
| 156 | /data5/data5/dongzhong/LymphGland/esophagus/2018-08-17/751696-27-1;8.tif | 1 | 0.91288235 | 0.996078431 | 0.981188235 |
| 157 | /data5/data5/dongzhong/LymphGland/esophagus/2018-08-17/747855-26-4;4.tif | 1 | 0.94984706 | 0.996078431 | 0.955329412 |
| 158 | /data5/data5/dongzhong/LymphGland/esophagus/2018-08-24/707307-31-4;5.tif | 1 | 0.98824706 | 0.996078431 | 0.996078431 |
| 159 | /data5/data5/dongzhong/LymphGland/esophagus/2018-11-02/783371-20-2;2.ndpi | 1 | 0.93512157 | 0.987921569 | 0.953752941 |
| 160 | /data5/data5/dongzhong/LymphGland/esophagus/2018-12-20/793533-25-2;2.ndpi | 1 | 0.94395294 | 0.991686275 | 0.996078431 |
| 161 | /data5/data5/dongzhong/LymphGland/esophagus/2018-11-09/771977-15-1;4.ndpi | 1 | 0.96579608 | 0.996078431 | 0.990470588 |
| 162 | /data5/data5/dongzhong/LymphGland/esophagus/2018-11-09/768709-13-5;5.ndpi | 1 | 0.95251373 | 0.994776471 | 0.983976471 |
| 163 | /data5/data5/dongzhong/LymphGland/esophagus/2018-11-02/778514-11-1;2.ndpi | 1 | 0.80389804 | 0.024956863 | 0.01467451 |
| 164 | /data5/data5/dongzhong/LymphGland/esophagus/2019-02-03/2018-12-27 19.46.39.ndpi | 1 | 0.90556471 | 0.972996078 | 0.986380392 |
| 165 | /data5/data5/dongzhong/LymphGland/esophagus/2018-08-17/723992-25-7;7.tif | 1 | 0.95783137 | 0.996078431 | 0.984180392 |
| 166 | /data5/data5/dongzhong/LymphGland/esophagus/2018-08-17/738868-17-2;7.tif | 1 | 0.96260392 | 0.996078431 | 0.996078431 |
| 167 | /data5/data5/dongzhong/LymphGland/esophagus/2018-08-17/747855-29-1;1.tif | 1 | 0.94534118 | 0.996078431 | 0.993396078 |
| 168 | /data5/data5/dongzhong/LymphGland/esophagus/2018-08-24/708900-8-1;3.tif | 1 | 0.9043098 | 0.996078431 | 0.995027451 |
| 169 | /data5/data5/dongzhong/LymphGland/esophagus/2018-11-02/786098-12-2;2.ndpi | 1 | 0.78161569 | 0.141941176 | 0.001654902 |
| 170 | /data5/data5/dongzhong/LymphGland/esophagus/2018-11-09/804579-19-1;1.ndpi | 1 | 0.84334902 | 0.256717647 | 7.84314E-06 |
| 171 | /data5/data5/dongzhong/LymphGland/esophagus/2018-11-09/769058-19-2;6.ndpi | 1 | 0.85854118 | 0.631623529 | 0.020345098 |
| 172 | /data5/data5/dongzhong/LymphGland/esophagus/2018-11-02/777581-9-1;4.ndpi | 1 | 0.89557647 | 0.550164706 | 0.018929412 |
| 173 | /data5/data5/dongzhong/LymphGland/esophagus/2019-02-03/802630-30-30 - 2019-01-11 15.17.08.ndpi | 1 | 0.92760392 | 0.971831373 | 0.996078431 |
| 174 | /data5/data5/dongzhong/LymphGland/esophagus/2018-08-17/723992-18-1;1.tif | 1 | 0.97658431 | 0.996078431 | 0.987101961 |
| 175 | /data5/data5/dongzhong/LymphGland/esophagus/2018-08-17/724177-16-1;8.tif | 1 | 0.68636863 | 0.875270588 | 0.833058824 |
| 176 | /data5/data5/dongzhong/LymphGland/esophagus/2018-08-17/736608-15-1;1.tif | 1 | 0.80488235 | 0.978764706 | 0.774396078 |
| 177 | /data5/data5/dongzhong/LymphGland/esophagus/2018-08-17/750621-17-1;3.tif | 1 | 0.89571373 | 0.996078431 | 0.991282353 |
| 178 | /data5/data5/dongzhong/LymphGland/esophagus/2018-08-17/747855-18-1;1.tif | 1 | 0.90786667 | 0.996078431 | 0.99232549 |
| 179 | /data5/data5/dongzhong/LymphGland/esophagus/2018-08-24/710821-11-1;1.tif | 1 | 0.96406275 | 0.996078431 | 0.996078431 |
| 180 | /data5/data5/dongzhong/LymphGland/esophagus/2018-08-24/708454-18-1;2.tif | 1 | 0.93777255 | 0.996078431 | 0.996078431 |
| 181 | /data5/data5/dongzhong/LymphGland/esophagus/2018-08-24/713984-19-1;1.tif | 1 | 0.96186667 | 0.996078431 | 0.996078431 |
| 182 | /data5/data5/dongzhong/LymphGland/esophagus/2018-08-24/709081-17-1;2.tif | 1 | 0.93633333 | 0.996078431 | 0.996078431 |
| 183 | /data5/data5/dongzhong/LymphGland/esophagus/2018-12-28/2018-12-27_15.54.43.ndpi | 1 | 0.92407451 | 0.994482353 | 0.990576471 |
| 184 | /data5/data5/dongzhong/LymphGland/esophagus/2018-12-28/2018-12-21_13.30.50.ndpi | 1 | 0.94689804 | 0.996078431 | 0.996078431 |
| 185 | /data5/data5/dongzhong/LymphGland/esophagus/2018-12-20/793533-29-2;5.ndpi | 1 | 0.78249804 | 0.89332549 | 0.64432549 |
| 186 | /data5/data5/dongzhong/LymphGland/esophagus/2018-11-09/773527-13-2;5.ndpi | 1 | 0.88657255 | 0.326745098 | 0.08685098 |
| 187 | /data5/data5/dongzhong/LymphGland/esophagus/2018-11-09/769058-18-3;3.ndpi | 1 | 0.89047843 | 0.755788235 | 0.487019608 |
| 188 | /data5/data5/dongzhong/LymphGland/esophagus/2018-08-17/723992-34-4;4.tif | 1 | 0.96223922 | 0.996078431 | 0.996078431 |
| 189 | /data5/data5/dongzhong/LymphGland/esophagus/2018-08-17/724177-13-1;2.tif | 1 | 0.83431373 | 0.995694118 | 0.990576471 |
| 190 | /data5/data5/dongzhong/LymphGland/esophagus/2018-08-17/738868-18-2;7.tif | 1 | 0.86841961 | 0.993733333 | 0.936956863 |
| 191 | /data5/data5/dongzhong/LymphGland/esophagus/2018-12-28/2018-12-27_16.09.19.ndpi | 1 | 0.94481961 | 0.996078431 | 0.928262745 |
| 192 | /data5/data5/dongzhong/LymphGland/esophagus/2018-12-20/793533-12-1;1.ndpi | 1 | 0.83585882 | 0.956176471 | 0.966109804 |
| 193 | /data5/data5/dongzhong/LymphGland/esophagus/2018-08-17/723992-37-1;1.tif | 1 | 0.96027059 | 0.996078431 | 0.995701961 |
| 194 | /data5/data5/dongzhong/LymphGland/esophagus/2018-08-17/724177-21-1;4.tif | 1 | 0.92452157 | 0.996078431 | 0.971937255 |
| 195 | /data5/data5/dongzhong/LymphGland/esophagus/2018-08-17/738868-21-2;2.tif | 1 | 0.9664 | 0.996078431 | 0.996078431 |
| 196 | /data5/data5/dongzhong/LymphGland/esophagus/2018-08-24/705235-22-1;2.tif | 1 | 0.96205098 | 0.996078431 | 0.996078431 |
| 197 | /data5/data5/dongzhong/LymphGland/esophagus/2018-11-02/781405-4-1;2.ndpi | 1 | 0.95398431 | 0.995286275 | 0.995760784 |
| 198 | /data5/data5/dongzhong/LymphGland/esophagus/2018-12-20/793533-26-2;2.ndpi | 1 | 0.91506275 | 0.992729412 | 0.990337255 |
| 199 | /data5/data5/dongzhong/LymphGland/esophagus/2019-02-03/801804-13-13 - 2019-01-11 11.53.08.ndpi | 1 | 0.91132157 | 0.990890196 | 0.996078431 |
| 200 | /data5/data5/dongzhong/LymphGland/esophagus/2018-08-24/705235-17-2;2.tif | 1 | 0.95876078 | 0.996078431 | 0.996078431 |
| 201 | /data5/data5/dongzhong/LymphGland/esophagus/2018-11-02/781405-5-2;2.ndpi | 1 | 0.91861961 | 0.965972549 | 0.962384314 |
| 202 | /data5/data5/dongzhong/LymphGland/esophagus/2018-12-20/794889-11-2;6.ndpi | 1 | 0.93490196 | 0.996078431 | 0.996078431 |
| 203 | /data5/data5/dongzhong/LymphGland/esophagus/2018-08-17/750621-15-2;6.tif | 1 | 0.95140784 | 0.996078431 | 0.992529412 |
| 204 | /data5/data5/dongzhong/LymphGland/esophagus/2018-12-20/793533-21-1;1.ndpi | 1 | 0.78907843 | 0.945435294 | 0.549901961 |
| 205 | /data5/data5/dongzhong/LymphGland/esophagus/2018-08-17/723992-40-2;2.tif | 1 | 0.95404314 | 0.996078431 | 0.992713725 |
| 206 | /data5/data5/dongzhong/LymphGland/esophagus/2018-08-17/750621-20-1;5.tif | 1 | 0.93114118 | 0.996078431 | 0.976454902 |
| 207 | /data5/data5/dongzhong/LymphGland/esophagus/2018-08-17/723992-29-5;5.tif | 1 | 0.95482745 | 0.996078431 | 0.991509804 |
| 208 | /data5/data5/dongzhong/LymphGland/esophagus/2018-08-17/724177-12-1;1.tif | 1 | 0.90998824 | 0.996078431 | 0.993447059 |
| 209 | /data5/data5/dongzhong/LymphGland/esophagus/2018-08-24/705235-14-3;3.tif | 1 | 0.94960784 | 0.996078431 | 0.996078431 |
| 210 | /data5/data5/dongzhong/LymphGland/esophagus/2018-08-24/708454-13-1;1.tif | 1 | 0.93763922 | 0.996078431 | 0.996078431 |
| 211 | /data5/data5/dongzhong/LymphGland/esophagus/2018-12-28/2018-12-21_09.25.58.ndpi | 1 | 0.83781569 | 0.604219608 | 0.813019608 |
| 212 | /data5/data5/dongzhong/LymphGland/esophagus/2018-12-20/793533-18-1;1.ndpi | 1 | 0.88649412 | 0.979968627 | 0.876305882 |
| 213 | /data5/data5/dongzhong/LymphGland/esophagus/2018-08-17/723992-23-5;5.tif | 1 | 0.96235686 | 0.996078431 | 0.990945098 |
| 214 | /data5/data5/dongzhong/LymphGland/esophagus/2018-08-17/724177-20-1;1.tif | 1 | 0.82679216 | 0.991137255 | 0.976858824 |
| 215 | /data5/data5/dongzhong/LymphGland/esophagus/2018-12-20/794889-15-1;1.ndpi | 1 | 0.93534902 | 0.99314902 | 0.996078431 |
| 216 | /data5/data5/dongzhong/LymphGland/esophagus/2018-08-17/723992-28-4;4.tif | 1 | 0.96013725 | 0.996078431 | 0.992913725 |
| 217 | /data5/data5/dongzhong/LymphGland/esophagus/2018-12-20/793533-16-2;2.ndpi | 1 | 0.8425098 | 0.809898039 | 0.779003922 |
| 218 | /data5/data5/dongzhong/LymphGland/esophagus/2018-08-17/736608-19-1;1.tif | 1 | 0.95547059 | 0.996078431 | 0.996078431 |
| 219 | /data5/data5/dongzhong/LymphGland/esophagus/2018-08-17/750621-14-4;5.tif | 1 | 0.97729804 | 0.996078431 | 0.996078431 |
| 220 | /data5/data5/dongzhong/LymphGland/esophagus/2018-08-24/705235-18-2;2.tif | 1 | 0.96109804 | 0.996078431 | 0.996078431 |
| 221 | /data5/data5/dongzhong/LymphGland/esophagus/2018-12-20/793533-24-1;1.ndpi | 1 | 0.90359216 | 0.989592157 | 0.996078431 |
| 222 | /data5/data5/dongzhong/LymphGland/esophagus/2018-08-17/723992-35-3;3.tif | 1 | 0.96795294 | 0.996078431 | 0.996078431 |
| 223 | /data6/医科院肿瘤医院正常和其他/2018-08-24/淋巴（食道）正常/714556-22-0;1.tif | 0 | 0.19676863 | 0.061219608 | 0.212580392 |
| 224 | /data6/医科院肿瘤医院正常和其他/2018-08-24/淋巴（食道）正常/719365-23-0;1.tif | 0 | 0.04644314 | 0.058341176 | 0.004862745 |
| 225 | /data5/data5/dongzhong/LymphGland/esophagus/2018-12-28/2018-12-20_18.50.38.ndpi | 0 | 0.26415686 | 0.00012549 | 0.034211765 |
| 226 | /data5/data5/dongzhong/LymphGland/esophagus/2018-12-28/2018-12-20_18.59.10.ndpi | 0 | 0.60889804 | 0.000737255 | 0.009592157 |
| 227 | /data5/data5/dongzhong/LymphGland/esophagus/2018-12-28/2018-12-20_19.07.44.ndpi | 0 | 0.60111373 | 0.007894118 | 0.207717647 |
| 228 | /data5/data5/dongzhong/LymphGland/esophagus/2018-12-28/2018-12-20_19.15.35.ndpi | 0 | 0.42215686 | 0.314070588 | 0.093152941 |
| 229 | /data5/data5/dongzhong/LymphGland/esophagus/2018-12-28/2018-12-20_19.19.22.ndpi | 0 | 0.54059608 | 0.377278431 | 0.076313725 |
| 230 | /data5/data5/dongzhong/LymphGland/esophagus/2018-12-28/2018-12-21_11.17.08.ndpi | 0 | 0.5278902 | 0.248784314 | 0.400537255 |
| 231 | /data6/医科院肿瘤医院正常和其他/2018-11-02/淋巴(食管)正常/790210-13-0;3.ndpi | 0 | 0.42237255 | 0.007576471 | 0.000870588 |
| 232 | /data5/data5/dongzhong/LymphGland/esophagus/2018-12-28/2018-12-21_09.34.50.ndpi | 0 | 0.70813333 | 0.376031373 | 0.131494118 |
| 233 | /data5/data5/dongzhong/LymphGland/esophagus/2018-12-28/2018-12-21_09.35.36.ndpi | 0 | 0.83232157 | 0.367133333 | 0.775431373 |
| 234 | /data5/data5/dongzhong/LymphGland/esophagus/2018-12-28/2018-12-21_09.45.09.ndpi | 0 | 0.48579216 | 0.059152941 | 0.050003922 |
| 235 | /data5/data5/dongzhong/LymphGland/esophagus/2018-12-28/2018-12-21_09.57.45.ndpi | 0 | 0.75770196 | 0.287431373 | 0.103003922 |
| 236 | /data5/data5/dongzhong/LymphGland/esophagus/2018-12-28/2018-12-21_10.06.05.ndpi | 0 | 0.90002745 | 0.98947451 | 0.97552549 |
| 237 | /data5/data5/dongzhong/LymphGland/esophagus/2018-12-28/2018-12-21_10.16.08.ndpi | 0 | 0.30113725 | 0.05685098 | 0.046913725 |
| 238 | /data5/data5/dongzhong/LymphGland/esophagus/2018-12-28/2018-12-21_10.27.31.ndpi | 0 | 0.41135686 | 0.016078431 | 0.027913725 |
| 239 | /data5/data5/dongzhong/LymphGland/esophagus/2018-12-28/2018-12-21_11.59.50.ndpi | 0 | 0.67357255 | 0.560152941 | 0.230423529 |
| 240 | /data5/data5/dongzhong/LymphGland/esophagus/2018-12-28/2018-12-21_12.01.37.ndpi | 0 | 0.71114118 | 0.132984314 | 0.6158 |
| 241 | /data6/医科院肿瘤医院正常和其他/2018-11-02/淋巴(食管)正常/781405-23-0;5.ndpi | 0 | 0.61993333 | 0.002698039 | 3.13725E-05 |
| 242 | /data6/医科院肿瘤医院正常和其他/2018-11-02/淋巴(食管)正常/781530-14-0;1.ndpi | 0 | 0.08107451 | 0.000227451 | 0 |
| 243 | /data5/data5/dongzhong/LymphGland/esophagus/2018-12-28/2018-12-24_15.35.55.ndpi | 0 | 0.90118039 | 0.993462745 | 0.981588235 |
| 244 | /data6/医科院肿瘤医院正常和其他/2018-11-02/淋巴(食管)正常/782266-13-0;2.ndpi | 0 | 0.09970196 | 7.84314E-05 | 0 |
| 245 | /data5/data5/dongzhong/LymphGland/esophagus/2018-12-28/2018-12-21_13.11.56.ndpi | 0 | 0.17158431 | 0.002258824 | 0.006631373 |
| 246 | /data5/data5/dongzhong/LymphGland/esophagus/2018-12-28/2018-12-27_15.53.47.ndpi | 0 | 0.37822745 | 0.449192157 | 0.264090196 |
| 247 | /data5/data5/dongzhong/LymphGland/esophagus/2018-12-28/2018-12-21_13.17.00.ndpi | 0 | 0.14927843 | 0.062360784 | 0.035709804 |
| 248 | /data6/医科院肿瘤医院正常和其他/2018-11-02/淋巴(食管)正常/783372-17-0;3.ndpi | 0 | 0.1506 | 0.000839216 | 0 |
| 249 | /data5/data5/dongzhong/LymphGland/esophagus/2018-12-28/2018-12-21_13.37.09.ndpi | 0 | 0.29271765 | 0.124403922 | 0.120082353 |
| 250 | /data5/data5/dongzhong/LymphGland/esophagus/2019-02-03/2018-12-27 18.41.39.ndpi | 0 | 0.45680784 | 0.159721569 | 0.087698039 |
| 251 | /data5/data5/dongzhong/LymphGland/esophagus/2018-12-20/2018-12-19 18.04.36.ndpi | 0 | 0.68212157 | 0.003737255 | 0.061003922 |
| 252 | /data5/data5/dongzhong/LymphGland/esophagus/2018-12-20/2018-12-19 18.15.51.ndpi | 0 | 0.16287059 | 0.046933333 | 0.039411765 |
| 253 | /data5/data5/dongzhong/LymphGland/esophagus/2018-12-20/2018-12-19 18.18.51.ndpi | 0 | 0.90787059 | 0.953164706 | 0.988043137 |
| 254 | /data5/data5/dongzhong/LymphGland/esophagus/2018-12-20/2018-12-19 18.30.22.ndpi | 0 | 0.87200392 | 0.961823529 | 0.064435294 |
| 255 | /data5/data5/dongzhong/LymphGland/esophagus/2018-12-20/2018-12-19 18.38.40.ndpi | 0 | 0.88747451 | 0.9798 | 0.644952941 |
| 256 | /data6/医科院肿瘤医院正常和其他/2018-11-02/淋巴(食管)正常/785318-13-0;3.ndpi | 0 | 0.16989804 | 1.96078E-05 | 5.09804E-05 |
| 257 | /data5/data5/dongzhong/LymphGland/esophagus/2018-12-20/2018-12-19 18.47.48.ndpi | 0 | 0.30203922 | 0.004886275 | 0.134392157 |
| 258 | /data5/data5/dongzhong/LymphGland/esophagus/2018-12-20/2018-12-19 18.59.13.ndpi | 0 | 0.81090196 | 0.093223529 | 0.357823529 |
| 259 | /data5/data5/dongzhong/LymphGland/esophagus/2018-12-20/2018-12-20 13.07.49.ndpi | 0 | 0.05761176 | 0 | 0.064937255 |
| 260 | /data5/data5/dongzhong/LymphGland/esophagus/2018-12-20/2018-12-19 19.11.43.ndpi | 0 | 0.73467843 | 0.446603922 | 0.034117647 |
| 261 | /data6/医科院肿瘤医院正常和其他/2018-11-02/淋巴(食管)正常/785912-11-0;3.ndpi | 0 | 0.82390196 | 0.526498039 | 0.052160784 |
| 262 | /data5/data5/dongzhong/LymphGland/esophagus/2018-12-20/2018-12-20 13.22.24.ndpi | 0 | 0.79559216 | 0.05574902 | 0.537203922 |
| 263 | /data5/data5/dongzhong/LymphGland/esophagus/2018-12-20/2018-12-19 19.35.50.ndpi | 0 | 0.64132941 | 0.065929412 | 0.137454902 |
| 264 | /data5/data5/dongzhong/LymphGland/esophagus/2018-12-20/2018-12-19 19.42.58.ndpi | 0 | 0.33187451 | 0.002988235 | 0.071262745 |
| 265 | /data5/data5/dongzhong/LymphGland/esophagus/2018-12-20/2018-12-20 13.44.59.ndpi | 0 | 0.68717255 | 0.112364706 | 0.119035294 |
| 266 | /data5/data5/dongzhong/LymphGland/esophagus/2018-12-20/2018-12-19 19.58.17.ndpi | 0 | 0.39285098 | 0.06005098 | 0.052129412 |
| 267 | /data5/data5/dongzhong/LymphGland/esophagus/2018-12-20/2018-12-19 20.08.56.ndpi | 0 | 0.6686 | 0.133121569 | 0.094984314 |
| 268 | /data6/医科院肿瘤医院正常和其他/2018-11-02/淋巴(食管)正常/787941-14-0;1.ndpi | 0 | 0.16793333 | 0.000156863 | 5.88235E-05 |
| 269 | /data6/医科院肿瘤医院正常和其他/2018-11-09/淋巴（食管）正常/787945-19-0;6.ndpi | 0 | 0.1914549 | 0.000203922 | 0.000152941 |
| 270 | /data5/data5/dongzhong/LymphGland/esophagus/2018-12-20/2018-12-20 15.00.52.ndpi | 0 | 0.55669412 | 0.404541176 | 0.23572549 |
| 271 | /data5/data5/dongzhong/LymphGland/esophagus/2018-12-20/2018-12-20 15.48.54.ndpi | 0 | 0.57843137 | 0.243780392 | 0.150960784 |
| 272 | /data5/data5/dongzhong/LymphGland/esophagus/2018-12-20/2018-12-20 15.06.40.ndpi | 0 | 0.51838039 | 0.560047059 | 0.060768627 |
| 273 | /data5/data5/dongzhong/LymphGland/esophagus/2018-12-20/790564-17-0;4.ndpi | 0 | 0.35900784 | 0.153796078 | 0.040921569 |
| 274 | /data5/data5/dongzhong/LymphGland/esophagus/2018-12-20/2018-12-20 15.10.52.ndpi | 0 | 0.79115686 | 0.095584314 | 0.094392157 |
| 275 | /data5/data5/dongzhong/LymphGland/esophagus/2018-12-20/790573-14-1;2.ndpi | 0 | 0.48106275 | 0.382729412 | 0.380235294 |
| 276 | /data5/data5/dongzhong/LymphGland/esophagus/2018-12-20/791683-19-0;4.ndpi | 0 | 0.20288235 | 0.057168627 | 0.031592157 |
| 277 | /data5/data5/dongzhong/LymphGland/esophagus/2018-12-20/792225-11-0;1.ndpi | 0 | 0.23830196 | 0.054109804 | 0.03725098 |
| 278 | /data5/data5/dongzhong/LymphGland/esophagus/2018-12-20/792483-14-0;3.ndpi | 0 | 0.34990196 | 0.440654902 | 0.177756863 |
| 279 | /data5/data5/dongzhong/LymphGland/esophagus/2018-12-20/792915-18-0;3.ndpi | 0 | 0.81631765 | 0.141239216 | 0.163058824 |
| 280 | /data5/data5/dongzhong/LymphGland/esophagus/2018-12-20/793059-16-0;2.ndpi | 0 | 0.18357255 | 0 | 0.012635294 |
| 281 | /data5/data5/dongzhong/LymphGland/esophagus/2018-12-20/793167-16-0;6.ndpi | 0 | 0.40118431 | 0.250862745 | 0.402109804 |
| 282 | /data5/data5/dongzhong/LymphGland/esophagus/2018-12-20/793533-19-0;1.ndpi | 0 | 0.45980784 | 0.212219608 | 0.06687451 |
| 283 | /data5/data5/dongzhong/LymphGland/esophagus/2018-12-20/793834-20-0;3.ndpi | 0 | 0.55958039 | 0.140792157 | 0.404623529 |
| 284 | /data5/data5/dongzhong/LymphGland/esophagus/2018-12-20/794196-12-0;5.ndpi | 0 | 0.23765882 | 0.768729412 | 0.090141176 |
| 285 | /data5/data5/dongzhong/LymphGland/esophagus/2018-12-20/794683-22-0;2.ndpi | 0 | 0.6628 | 0.266835294 | 0.214403922 |
| 286 | /data5/data5/dongzhong/LymphGland/esophagus/2018-12-20/794889-18-0;1.ndpi | 0 | 0.25248235 | 0.041372549 | 0.21387451 |
| 287 | /data5/data5/dongzhong/LymphGland/esophagus/2018-12-20/795608-17-0；5.ndpi | 0 | 0.61123922 | 0.263011765 | 0.111945098 |
| 288 | /data5/data5/dongzhong/LymphGland/esophagus/2018-12-20/795928-13-0;1.ndpi | 0 | 0.82764706 | 0.340713725 | 0.986819608 |
| 289 | /data6/医科院肿瘤医院正常和其他/2018-11-02/淋巴(食管)正常/795996-13-0;2.ndpi | 0 | 0.02343922 | 0.004658824 | 0.008866667 |
| 290 | /data5/data5/dongzhong/LymphGland/esophagus/2018-12-20/796186-18-0;4.ndpi | 0 | 0.78059608 | 0.455831373 | 0.819996078 |
| 291 | /data5/data5/dongzhong/LymphGland/esophagus/2018-12-20/796657-26-0;3.ndpi | 0 | 0.72752941 | 0.397945098 | 0.289023529 |
| 292 | /data5/data5/dongzhong/LymphGland/esophagus/2018-12-20/797267-2-0;2.ndpi | 0 | 0.41204706 | 0.278682353 | 0.163772549 |
| 293 | /data5/data5/dongzhong/LymphGland/esophagus/2018-12-20/797479-12-0;3.ndpi | 0 | 0.07931373 | 0.008223529 | 0.005580392 |
| 294 | /data5/data5/dongzhong/LymphGland/esophagus/2018-12-20/797701-15-0;5.ndpi | 0 | 0.55818431 | 0.369752941 | 0.04467451 |
| 295 | /data5/data5/dongzhong/LymphGland/esophagus/2018-12-20/798305-13-0;4.ndpi | 0 | 0.53118824 | 0.58207451 | 0.379345098 |
| 296 | /data5/data5/dongzhong/LymphGland/esophagus/2018-12-20/798347-18-0;2.ndpi | 0 | 0.56453333 | 0.10707451 | 0.08312549 |
| 297 | /data5/data5/dongzhong/LymphGland/esophagus/2018-12-20/798348-20-0；2.ndpi | 0 | 0.10667451 | 0.00627451 | 0.008552941 |
| 298 | /data5/data5/dongzhong/LymphGland/esophagus/2018-12-20/798363-18-0；1.ndpi | 0 | 0.26794902 | 0.002294118 | 0.012082353 |
| 299 | /data5/data5/dongzhong/LymphGland/esophagus/2018-12-20/798467-18-0；2.ndpi | 0 | 0.00553725 | 0 | 0.000396078 |
| 300 | /data5/data5/dongzhong/LymphGland/esophagus/2018-12-20/798539-25-0;1.ndpi | 0 | 0.32079608 | 0.492043137 | 0.046623529 |
| 301 | /data6/医科院肿瘤医院正常和其他/2018-11-09/淋巴（食管）正常/804579-15-0;1.ndpi | 0 | 0.8415451 | 0.146015686 | 3.92157E-06 |
| 302 | /data6/医科院肿瘤医院正常和其他/2018-11-09/淋巴（食管）正常/805750-21-0;3.ndpi | 0 | 0.03663922 | 0 | 0 |
| 303 | /data6/医科院肿瘤医院正常和其他/2018-11-09/淋巴（食管）正常/775705-17-0;3.ndpi | 0 | 0.69167059 | 0.233396078 | 2.7451E-05 |
| 304 | /data6/医科院肿瘤医院正常和其他/2018-11-09/淋巴（食管）正常/775522-15-0;1.ndpi | 0 | 0.6417098 | 0 | 0 |
| 305 | /data6/医科院肿瘤医院正常和其他/2018-11-09/淋巴（食管）正常/773527-14-0;7.ndpi | 0 | 0.47691373 | 0.01892549 | 1.56863E-05 |
| 306 | /data6/医科院肿瘤医院正常和其他/2018-11-09/淋巴（食管）正常/773083-10-0;1.ndpi | 0 | 0.29952157 | 0.00165098 | 1.96078E-05 |
| 307 | /data6/医科院肿瘤医院正常和其他/2018-11-09/淋巴（食管）正常/771977-14-0;1.ndpi | 0 | 0.65392549 | 0.010901961 | 0.000486275 |
| 308 | /data6/医科院肿瘤医院正常和其他/2018-11-09/淋巴（食管）正常/768903-10-0;3.ndpi | 0 | 0.06125882 | 0 | 0 |
| 309 | /data6/医科院肿瘤医院正常和其他/2018-11-09/淋巴（食管）正常/768724-13-0;4.ndpi | 0 | 0.43265882 | 0.035172549 | 2.7451E-05 |
| 310 | /data6/医科院肿瘤医院正常和其他/2018-11-09/淋巴（食管）正常/768709-15-0;5.ndpi | 0 | 0.64750196 | 0.091047059 | 3.92157E-06 |
| 311 | /data5/data5/dongzhong/LymphGland/esophagus/2019-02-03/2018-12-27 19.35.03.ndpi | 0 | 0.53465882 | 0.024415686 | 0.208917647 |
| 312 | /data5/data5/dongzhong/LymphGland/esophagus/2019-02-03/2018-12-27 19.03.56.ndpi | 0 | 0.62356471 | 0.15185098 | 0.600517647 |
| 313 | /data6/医科院肿瘤医院正常和其他/2018-11-02/淋巴(食管)正常/776561-13-0;3.ndpi | 0 | 0.76381176 | 0.007160784 | 6.27451E-05 |
| 314 | /data6/医科院肿瘤医院正常和其他/2018-11-02/淋巴(食管)正常/776703-22-0;2.ndpi | 0 | 0.06441569 | 0 | 3.92157E-06 |
| 315 | /data6/医科院肿瘤医院正常和其他/2018-11-02/淋巴(食管)正常/777581-19-0;2.ndpi | 0 | 0.58623922 | 6.66667E-05 | 0.002254902 |
| 316 | /data6/医科院肿瘤医院正常和其他/2018-11-02/淋巴(食管)正常/778514-14-0;1.ndpi | 0 | 0.76612941 | 0.002717647 | 0 |
| 317 | /data5/data5/dongzhong/LymphGland/esophagus/2019-02-03/2018-12-27 18.58.33.ndpi | 0 | 0.17450196 | 0.01214902 | 0.096196078 |
| 318 | /data5/data5/dongzhong/LymphGland/esophagus/2019-02-03/2018-12-27 19.14.45.ndpi | 0 | 0.91512941 | 0.947090196 | 0.073160784 |
| 319 | /data5/data5/dongzhong/LymphGland/esophagus/2019-02-03/2018-12-27 19.26.40.ndpi | 0 | 0.09971765 | 0.000168627 | 0.046588235 |
| 320 | /data5/data5/dongzhong/LymphGland/esophagus/2019-02-03/2018-12-27 19.30.10.ndpi | 0 | 0.43392941 | 0.09145098 | 0.166090196 |
| 321 | /data5/data5/dongzhong/LymphGland/esophagus/2019-02-03/2018-12-27 19.45.33.ndpi | 0 | 0.35112157 | 0.137721569 | 0.460086275 |
| 322 | /data5/data5/dongzhong/LymphGland/esophagus/2019-02-03/2018-12-27 19.51.21.ndpi | 0 | 0.59140784 | 0.333321569 | 0.962713725 |
| 323 | /data5/data5/dongzhong/LymphGland/esophagus/2019-02-03/800190-19-19 - 2019-01-11 10.42.52.ndpi | 0 | 0.40607843 | 0.108207843 | 0.030737255 |
| 324 | /data5/data5/dongzhong/LymphGland/esophagus/2019-02-03/800368-18-18 - 2019-01-11 10.48.26.ndpi | 0 | 0.70309412 | 0.809015686 | 0.153407843 |
| 325 | /data5/data5/dongzhong/LymphGland/esophagus/2019-02-03/800400-39-39 - 2019-01-11 11.02.48.ndpi | 0 | 0.28155686 | 0.06054902 | 0.056188235 |
| 326 | /data5/data5/dongzhong/LymphGland/esophagus/2019-02-03/800645-18-18 - 2019-01-11 11.15.27.ndpi | 0 | 0.49156863 | 0.140741176 | 0.105368627 |
| 327 | /data5/data5/dongzhong/LymphGland/esophagus/2019-02-03/800811-12-12 - 2019-01-11 14.38.18.ndpi | 0 | 0.71489412 | 0.894796078 | 0.257764706 |
| 328 | /data5/data5/dongzhong/LymphGland/esophagus/2019-02-03/801225-17-17 - 2019-01-11 11.24.34.ndpi | 0 | 0.2397098 | 0.22012549 | 0.209 |
| 329 | /data5/data5/dongzhong/LymphGland/esophagus/2019-02-03/801343-18-18 - 2019-01-11 11.30.22.ndpi | 0 | 0.28509412 | 0.430819608 | 0.076721569 |
| 330 | /data5/data5/dongzhong/LymphGland/esophagus/2019-02-03/801625-10-10 - 2019-01-11 10.35.27.ndpi | 0 | 0.50248627 | 0.011498039 | 0.289062745 |
| 331 | /data5/data5/dongzhong/LymphGland/esophagus/2019-02-03/801768-2-2 - 2019-01-11 11.48.33.ndpi | 0 | 0.03679216 | 9.41176E-05 | 0.014541176 |
| 332 | /data5/data5/dongzhong/LymphGland/esophagus/2019-02-03/801804-9-9 - 2019-01-11 11.49.35.ndpi | 0 | 0.54183137 | 0.161466667 | 0.085639216 |
| 333 | /data5/data5/dongzhong/LymphGland/esophagus/2019-02-03/802277-17-17 - 2019-01-11 12.12.29.ndpi | 0 | 0.81932941 | 0.353235294 | 0.051607843 |
| 334 | /data5/data5/dongzhong/LymphGland/esophagus/2019-02-03/802465-13-13 - 2019-01-11 12.20.46.ndpi | 0 | 0.49741569 | 0.026537255 | 0.029686275 |
| 335 | /data5/data5/dongzhong/LymphGland/esophagus/2019-02-03/802501-24-24 - 2019-01-11 12.38.58.ndpi | 0 | 0.36276863 | 0.193117647 | 0.013447059 |
| 336 | /data5/data5/dongzhong/LymphGland/esophagus/2019-02-03/802624-13-13 - 2019-01-11 15.10.06.ndpi | 0 | 0.13360784 | 0 | 0.267156863 |
| 337 | /data5/data5/dongzhong/LymphGland/esophagus/2019-02-03/802629-15-15 - 2019-01-11 12.49.12.ndpi | 0 | 0.15070196 | 0.018407843 | 0.040866667 |
| 338 | /data5/data5/dongzhong/LymphGland/esophagus/2019-02-03/802630-22-22 - 2019-01-11 12.57.17.ndpi | 0 | 0.77204706 | 0.835513725 | 0.292031373 |
| 339 | /data5/data5/dongzhong/LymphGland/esophagus/2019-02-03/802679-14-14 - 2019-01-11 13.07.59.ndpi | 0 | 0.12441569 | 0.036847059 | 0.084223529 |
| 340 | /data5/data5/dongzhong/LymphGland/esophagus/2019-02-03/802855-16-16 - 2019-01-11 15.29.53.ndpi | 0 | 0.61204314 | 0.8324 | 0.592792157 |
| 341 | /data5/data5/dongzhong/LymphGland/esophagus/2019-02-03/803307-15-15 - 2019-01-11 13.36.48.ndpi | 0 | 0.60295294 | 0.748258824 | 0.0924 |
| 342 | /data5/data5/dongzhong/LymphGland/esophagus/2019-02-03/803309-15-15 - 2019-01-11 15.36.30.ndpi | 0 | 0.13117647 | 0 | 0.005847059 |
| 343 | /data5/data5/dongzhong/LymphGland/esophagus/2019-02-03/803342-16-16 - 2019-01-11 13.54.08.ndpi | 0 | 0.40339608 | 0.062490196 | 0.030937255 |
| 344 | /data5/data5/dongzhong/LymphGland/esophagus/2019-02-03/803453-23-23 - 2019-01-11 14.04.44.ndpi | 0 | 0.26850196 | 0.052141176 | 0.10434902 |
| 345 | /data5/data5/dongzhong/LymphGland/esophagus/2019-02-03/803626-14-14 - 2019-01-11 14.31.25.ndpi | 0 | 0.20176471 | 0.042117647 | 0.129376471 |
| 346 | /data6/医科院肿瘤医院正常和其他/2018-11-09/淋巴（食管）正常/805556-9-0;3.ndpi | 0 | 0.36172549 | 0 | 0 |
| 347 | /data6/医科院肿瘤医院正常和其他/2018-08-24/淋巴（食道）正常/714556-22-0;1.tif | 0 | 0.19676863 | 0.061219608 | 0.212580392 |
| 348 | /data6/医科院肿瘤医院正常和其他/2018-08-24/淋巴（食道）正常/719365-23-0;1.tif | 0 | 0.04644314 | 0.058341176 | 0.004862745 |
| 349 | /data5/data5/dongzhong/LymphGland/esophagus/2018-12-28/2018-12-20_18.50.38.ndpi | 0 | 0.26415686 | 0.00012549 | 0.034211765 |
| 350 | /data5/data5/dongzhong/LymphGland/esophagus/2018-12-28/2018-12-20_18.56.12.ndpi | 0 | 0.29887843 | 0.001831373 | 0.029772549 |
| 351 | /data5/data5/dongzhong/LymphGland/esophagus/2018-12-28/2018-12-21_11.14.39.ndpi | 0 | 0.3640902 | 0.003176471 | 0.057984314 |
| 352 | /data6/医科院肿瘤医院正常和其他/2018-11-09/淋巴（食管）正常/789261-18-0;2.ndpi | 0 | 0.2517098 | 0.001407843 | 0 |
| 353 | /data5/data5/dongzhong/LymphGland/esophagus/2018-12-28/2018-12-20_19.19.22.ndpi | 0 | 0.54059608 | 0.377278431 | 0.076313725 |
| 354 | /data5/data5/dongzhong/LymphGland/esophagus/2018-12-28/2018-12-21_09.26.36.ndpi | 0 | 0.44911373 | 0.293027451 | 0.144647059 |
| 355 | /data5/data5/dongzhong/LymphGland/esophagus/2018-12-28/2018-12-25_14.31.19.ndpi | 0 | 0.7420902 | 0.664917647 | 0.642937255 |
| 356 | /data5/data5/dongzhong/LymphGland/esophagus/2018-12-28/2018-12-21_09.33.57.ndpi | 0 | 0.41903529 | 0.702133333 | 0.080172549 |
| 357 | /data5/data5/dongzhong/LymphGland/esophagus/2018-12-28/2018-12-21_09.38.25.ndpi | 0 | 0.55252941 | 0.234764706 | 0.219298039 |
| 358 | /data5/data5/dongzhong/LymphGland/esophagus/2018-12-28/2018-12-21_09.42.10.ndpi | 0 | 0.34878039 | 0.027301961 | 0.004364706 |
| 359 | /data5/data5/dongzhong/LymphGland/esophagus/2018-12-28/2018-12-21_10.02.49.ndpi | 0 | 0.77454902 | 0.000435294 | 0.012286275 |
| 360 | /data5/data5/dongzhong/LymphGland/esophagus/2018-12-28/2018-12-21_10.10.04.ndpi | 0 | 0.22419608 | 0.003886275 | 0.023368627 |
| 361 | /data5/data5/dongzhong/LymphGland/esophagus/2018-12-28/2018-12-21_10.24.11.ndpi | 0 | 0.53608627 | 0.086666667 | 0.05447451 |
| 362 | /data5/data5/dongzhong/LymphGland/esophagus/2018-12-28/2018-12-25_14.33.40.ndpi | 0 | 0.33549804 | 0.000388235 | 0.019537255 |
| 363 | /data5/data5/dongzhong/LymphGland/esophagus/2018-12-28/2018-12-27_16.17.10.ndpi | 0 | 0.17386275 | 0.000886275 | 0.018015686 |
| 364 | /data5/data5/dongzhong/LymphGland/esophagus/2018-12-28/2018-12-21_12.00.56.ndpi | 0 | 0.64093333 | 0.007337255 | 0.355776471 |
| 365 | /data6/医科院肿瘤医院正常和其他/2018-11-02/淋巴(食管)正常/781405-24-0;1.ndpi | 0 | 0.26770196 | 0 | 0 |
| 366 | /data5/data5/dongzhong/LymphGland/esophagus/2018-12-28/2018-12-21_12.35.08.ndpi | 0 | 0.06500392 | 0 | 0.016027451 |
| 367 | /data5/data5/dongzhong/LymphGland/esophagus/2018-12-28/2018-12-21_12.58.33.ndpi | 0 | 0.18036078 | 0.003223529 | 0.103117647 |
| 368 | /data6/医科院肿瘤医院正常和其他/2018-11-02/淋巴(食管)正常/782266-13-0;2.ndpi | 0 | 0.09970196 | 7.84314E-05 | 0 |
| 369 | /data5/data5/dongzhong/LymphGland/esophagus/2018-12-28/2018-12-21_13.10.09.ndpi | 0 | 0.66126667 | 0.818635294 | 0.40412549 |
| 370 | /data5/data5/dongzhong/LymphGland/esophagus/2018-12-28/2018-12-27_15.52.14.ndpi | 0 | 0.49777255 | 0.596062745 | 0.251466667 |
| 371 | /data5/data5/dongzhong/LymphGland/esophagus/2018-12-28/2018-12-21_13.21.15.ndpi | 0 | 0.4077098 | 0.417901961 | 0.17765098 |
| 372 | /data6/医科院肿瘤医院正常和其他/2018-11-02/淋巴(食管)正常/783372-17-0;3.ndpi | 0 | 0.1506 | 0.000839216 | 0 |
| 373 | /data5/data5/dongzhong/LymphGland/esophagus/2018-12-28/2018-12-21_13.32.29.ndpi | 0 | 0.5079098 | 0.788427451 | 0.117658824 |
| 374 | /data5/data5/dongzhong/LymphGland/esophagus/2019-02-03/2018-12-27 18.41.39.ndpi | 0 | 0.45680784 | 0.159721569 | 0.087698039 |
| 375 | /data5/data5/dongzhong/LymphGland/esophagus/2018-12-20/797114-10-0;2.ndpi | 0 | 0.55088235 | 0.491054902 | 0.041576471 |
| 376 | /data5/data5/dongzhong/LymphGland/esophagus/2018-12-20/2018-12-19 18.15.51.ndpi | 0 | 0.16287059 | 0.046933333 | 0.039411765 |
| 377 | /data5/data5/dongzhong/LymphGland/esophagus/2018-12-20/2018-12-19 18.24.37.ndpi | 0 | 0.40596078 | 0.419509804 | 0.098145098 |
| 378 | /data5/data5/dongzhong/LymphGland/esophagus/2018-12-20/2018-12-19 18.32.39.ndpi | 0 | 0.16175294 | 0.009682353 | 0.02025098 |
| 379 | /data5/data5/dongzhong/LymphGland/esophagus/2018-12-20/2018-12-19 18.36.54.ndpi | 0 | 0.83950588 | 0.771494118 | 0.974458824 |
| 380 | /data6/医科院肿瘤医院正常和其他/2018-11-02/淋巴(食管)正常/785318-13-0;3.ndpi | 0 | 0.16989804 | 1.96078E-05 | 5.09804E-05 |
| 381 | /data5/data5/dongzhong/LymphGland/esophagus/2018-12-20/2018-12-20 12.56.34.ndpi | 0 | 0.33305098 | 0.018807843 | 0.033388235 |
| 382 | /data5/data5/dongzhong/LymphGland/esophagus/2018-12-20/2018-12-20 13.04.57.ndpi | 0 | 0.83664706 | 0.987890196 | 0.981192157 |
| 383 | /data5/data5/dongzhong/LymphGland/esophagus/2018-12-20/2018-12-20 13.06.27.ndpi | 0 | 0.1127451 | 0.000137255 | 0.143039216 |
| 384 | /data5/data5/dongzhong/LymphGland/esophagus/2018-12-20/2018-12-19 19.09.37.ndpi | 0 | 0.34394118 | 0.497152941 | 0.024996078 |
| 385 | /data5/data5/dongzhong/LymphGland/esophagus/2018-12-20/2018-12-19 19.15.16.ndpi | 0 | 0.87549412 | 0.763443137 | 0.934513725 |
| 386 | /data5/data5/dongzhong/LymphGland/esophagus/2018-12-20/2018-12-19 19.22.33.ndpi | 0 | 0.27408627 | 0.003517647 | 0.048372549 |
| 387 | /data5/data5/dongzhong/LymphGland/esophagus/2018-12-20/2018-12-19 19.32.59.ndpi | 0 | 0.06294118 | 2.35294E-05 | 0.000156863 |
| 388 | /data5/data5/dongzhong/LymphGland/esophagus/2018-12-20/2018-12-19 19.41.33.ndpi | 0 | 0.68525098 | 0.091945098 | 0.01427451 |
| 389 | /data5/data5/dongzhong/LymphGland/esophagus/2018-12-20/2018-12-19 19.52.07.ndpi | 0 | 0.671 | 0.097956863 | 0.090352941 |
| 390 | /data5/data5/dongzhong/LymphGland/esophagus/2018-12-20/2018-12-19 19.58.17.ndpi | 0 | 0.39285098 | 0.06005098 | 0.052129412 |
| 391 | /data5/data5/dongzhong/LymphGland/esophagus/2018-12-20/2018-12-19 20.03.14.ndpi | 0 | 0.59082745 | 0.069113725 | 0.158854902 |
| 392 | /data5/data5/dongzhong/LymphGland/esophagus/2018-12-20/2018-12-20 14.49.33.ndpi | 0 | 0.87170196 | 0.002835294 | 0.046556863 |
| 393 | /data5/data5/dongzhong/LymphGland/esophagus/2018-12-20/2018-12-20 14.55.14.ndpi | 0 | 0.29701569 | 0.00165098 | 0.03054902 |
| 394 | /data5/data5/dongzhong/LymphGland/esophagus/2018-12-20/2018-12-20 15.00.52.ndpi | 0 | 0.55669412 | 0.404541176 | 0.23572549 |
| 395 | /data5/data5/dongzhong/LymphGland/esophagus/2018-12-20/2018-12-20 15.47.21.ndpi | 0 | 0.44010196 | 0.317054902 | 0.132627451 |
| 396 | /data5/data5/dongzhong/LymphGland/esophagus/2018-12-20/2018-12-20 15.09.44.ndpi | 0 | 0.57285098 | 0 | 0.188764706 |
| 397 | /data5/data5/dongzhong/LymphGland/esophagus/2018-12-20/790564-17-0;4.ndpi | 0 | 0.35900784 | 0.153796078 | 0.040921569 |
| 398 | /data5/data5/dongzhong/LymphGland/esophagus/2018-12-20/2018-12-20 15.12.07.ndpi | 0 | 0.76763922 | 0.198772549 | 0.111188235 |
| 399 | /data5/data5/dongzhong/LymphGland/esophagus/2018-12-20/790573-19-0;2.ndpi | 0 | 0.52785882 | 0.706976471 | 0.102803922 |
| 400 | /data5/data5/dongzhong/LymphGland/esophagus/2018-12-20/791683-21-0;1.ndpi | 0 | 0.38535686 | 0.369011765 | 0.061062745 |
| 401 | /data5/data5/dongzhong/LymphGland/esophagus/2018-12-20/792225-11-0;1.ndpi | 0 | 0.23830196 | 0.054109804 | 0.03725098 |
| 402 | /data5/data5/dongzhong/LymphGland/esophagus/2018-12-20/792483-12-0;4.ndpi | 0 | 0.08475294 | 0 | 0.010294118 |
| 403 | /data5/data5/dongzhong/LymphGland/esophagus/2018-12-20/792915-17-0;3.ndpi | 0 | 0.32277647 | 0.077729412 | 0.017647059 |
| 404 | /data5/data5/dongzhong/LymphGland/esophagus/2018-12-20/793059-16-0;2.ndpi | 0 | 0.18357255 | 0 | 0.012635294 |
| 405 | /data5/data5/dongzhong/LymphGland/esophagus/2018-12-20/793167-19-0;3.ndpi | 0 | 0.34977255 | 0.052098039 | 0.195333333 |
| 406 | /data5/data5/dongzhong/LymphGland/esophagus/2018-12-20/793533-17-0;1.ndpi | 0 | 0.04261569 | 0 | 0.002988235 |
| 407 | /data5/data5/dongzhong/LymphGland/esophagus/2018-12-20/793834-21-0;1.ndpi | 0 | 0.40701176 | 0.001301961 | 0.009996078 |
| 408 | /data5/data5/dongzhong/LymphGland/esophagus/2018-12-20/794196-17-0;1.ndpi | 0 | 0.54856471 | 0.30314902 | 0.051196078 |
| 409 | /data5/data5/dongzhong/LymphGland/esophagus/2018-12-20/794683-21-0;3.ndpi | 0 | 0.49619608 | 0.317447059 | 0.065972549 |
| 410 | /data6/医科院肿瘤医院正常和其他/2018-11-02/淋巴(食管)正常/794889-8-0;1.ndpi | 0 | 0.098 | 0.000364706 | 0 |
| 411 | /data5/data5/dongzhong/LymphGland/esophagus/2018-12-20/795608-19-0；1.ndpi | 0 | 0.47528627 | 0.01167451 | 0.033937255 |
| 412 | /data5/data5/dongzhong/LymphGland/esophagus/2018-12-20/795928-13-0;1.ndpi | 0 | 0.82764706 | 0.340713725 | 0.986819608 |
| 413 | /data5/data5/dongzhong/LymphGland/esophagus/2018-12-20/795996-17-0;4.ndpi | 0 | 0.59767059 | 0.438819608 | 0.150996078 |
| 414 | /data5/data5/dongzhong/LymphGland/esophagus/2018-12-20/796186-18-0;4.ndpi | 0 | 0.78059608 | 0.455831373 | 0.819996078 |
| 415 | /data5/data5/dongzhong/LymphGland/esophagus/2018-12-20/796657-23-0;3.ndpi | 0 | 0.41260392 | 0.398023529 | 0.189980392 |
| 416 | /data5/data5/dongzhong/LymphGland/esophagus/2018-12-20/797267-3-0;2.ndpi | 0 | 0.17970588 | 0 | 2.35294E-05 |
| 417 | /data5/data5/dongzhong/LymphGland/esophagus/2018-12-20/797479-12-0;3.ndpi | 0 | 0.07931373 | 0.008223529 | 0.005580392 |
| 418 | /data5/data5/dongzhong/LymphGland/esophagus/2018-12-20/797701-13-0;6.ndpi | 0 | 0.68288627 | 0.632933333 | 0.338505882 |
| 419 | /data5/data5/dongzhong/LymphGland/esophagus/2018-12-20/798305-15-0;1.ndpi | 0 | 0.18244314 | 0.220031373 | 0.034082353 |
| 420 | /data5/data5/dongzhong/LymphGland/esophagus/2018-12-20/798347-17-0;2.ndpi | 0 | 0.7811098 | 0.524992157 | 0.13647451 |
| 421 | /data5/data5/dongzhong/LymphGland/esophagus/2018-12-20/798348-19-0；2.ndpi | 0 | 0.88883137 | 0.219945098 | 0.027984314 |
| 422 | /data5/data5/dongzhong/LymphGland/esophagus/2018-12-20/798363-23-0；4.ndpi | 0 | 0.2383451 | 0.041619608 | 0.381513725 |
| 423 | /data5/data5/dongzhong/LymphGland/esophagus/2018-12-20/798467-10-0;1.ndpi | 0 | 0.13389804 | 0.00134902 | 0.034407843 |
| 424 | /data5/data5/dongzhong/LymphGland/esophagus/2018-12-20/798539-27-0;1.ndpi | 0 | 0.90446275 | 0.62794902 | 0.08207451 |
| 425 | /data6/医科院肿瘤医院正常和其他/2018-11-09/淋巴（食管）正常/804579-16-0;5.ndpi | 0 | 0.92322745 | 0.221 | 9.41176E-05 |
| 426 | /data6/医科院肿瘤医院正常和其他/2018-11-09/淋巴（食管）正常/805750-20-0;2.ndpi | 0 | 0.14608235 | 0.003745098 | 5.4902E-05 |
| 427 | /data6/医科院肿瘤医院正常和其他/2018-11-09/淋巴（食管）正常/775705-18-0;3.ndpi | 0 | 0.66328235 | 0.109623529 | 0.000141176 |
| 428 | /data6/医科院肿瘤医院正常和其他/2018-11-09/淋巴（食管）正常/775522-15-0;1.ndpi | 0 | 0.6417098 | 0 | 0 |
| 429 | /data6/医科院肿瘤医院正常和其他/2018-11-09/淋巴（食管）正常/773527-15-0;2.ndpi | 0 | 0.4695451 | 4.31373E-05 | 0 |
| 430 | /data6/医科院肿瘤医院正常和其他/2018-11-09/淋巴（食管）正常/773083-10-0;1.ndpi | 0 | 0.29952157 | 0.00165098 | 1.96078E-05 |
| 431 | /data6/医科院肿瘤医院正常和其他/2018-11-09/淋巴（食管）正常/771977-10-0;1.ndpi | 0 | 0.3483451 | 0.000552941 | 0 |
| 432 | /data6/医科院肿瘤医院正常和其他/2018-11-09/淋巴（食管）正常/768903-10-0;3.ndpi | 0 | 0.06125882 | 0 | 0 |
| 433 | /data6/医科院肿瘤医院正常和其他/2018-11-09/淋巴（食管）正常/768724-13-0;4.ndpi | 0 | 0.43265882 | 0.035172549 | 2.7451E-05 |
| 434 | /data6/医科院肿瘤医院正常和其他/2018-11-09/淋巴（食管）正常/768709-15-0;5.ndpi | 0 | 0.64750196 | 0.091047059 | 3.92157E-06 |
| 435 | /data5/data5/dongzhong/LymphGland/esophagus/2019-02-03/2018-12-27 19.35.03.ndpi | 0 | 0.53465882 | 0.024415686 | 0.208917647 |
| 436 | /data5/data5/dongzhong/LymphGland/esophagus/2019-02-03/2018-12-27 19.02.35.ndpi | 0 | 0.69249412 | 0.148992157 | 0.610752941 |
| 437 | /data6/医科院肿瘤医院正常和其他/2018-11-02/淋巴(食管)正常/776561-12-0；1.ndpi | 0 | 0.0584549 | 0 | 0 |
| 438 | /data6/医科院肿瘤医院正常和其他/2018-11-02/淋巴(食管)正常/776703-21-0;3.ndpi | 0 | 0.3238549 | 0.001509804 | 0 |
| 439 | /data6/医科院肿瘤医院正常和其他/2018-11-02/淋巴(食管)正常/777581-17-0;2.ndpi | 0 | 0.61186275 | 0.002137255 | 0.000192157 |
| 440 | /data6/医科院肿瘤医院正常和其他/2018-11-02/淋巴(食管)正常/778514-14-0;1.ndpi | 0 | 0.76612941 | 0.002717647 | 0 |
| 441 | /data5/data5/dongzhong/LymphGland/esophagus/2019-02-03/2018-12-27 18.58.33.ndpi | 0 | 0.17450196 | 0.01214902 | 0.096196078 |
| 442 | /data5/data5/dongzhong/LymphGland/esophagus/2019-02-03/2018-12-27 19.18.31.ndpi | 0 | 0.27318039 | 0.383647059 | 0.036882353 |
| 443 | /data5/data5/dongzhong/LymphGland/esophagus/2019-02-03/2018-12-27 19.21.48.ndpi | 0 | 0.38638039 | 0.483733333 | 0.091580392 |
| 444 | /data5/data5/dongzhong/LymphGland/esophagus/2019-02-03/2018-12-27 19.27.23.ndpi | 0 | 0.46638431 | 0.05834902 | 0.046988235 |
| 445 | /data5/data5/dongzhong/LymphGland/esophagus/2019-02-03/2018-12-27 19.47.50.ndpi | 0 | 0.36119608 | 0.046643137 | 0.303898039 |
| 446 | /data5/data5/dongzhong/LymphGland/esophagus/2019-02-03/2018-12-27 19.52.49.ndpi | 0 | 0.4551098 | 0.281145098 | 0.565184314 |
| 447 | /data5/data5/dongzhong/LymphGland/esophagus/2019-02-03/800190-20-20 - 2019-01-11 10.44.38.ndpi | 0 | 0.2731451 | 0.252282353 | 0.072196078 |
| 448 | /data5/data5/dongzhong/LymphGland/esophagus/2019-02-03/800368-19-19 - 2019-01-11 10.50.09.ndpi | 0 | 0.46072549 | 0.591792157 | 0.081611765 |
| 449 | /data5/data5/dongzhong/LymphGland/esophagus/2019-02-03/800400-35-35 - 2019-01-11 11.00.32.ndpi | 0 | 0.48520392 | 0.09067451 | 0.5138 |
| 450 | /data5/data5/dongzhong/LymphGland/esophagus/2019-02-03/800645-18-18 - 2019-01-11 11.15.27.ndpi | 0 | 0.49156863 | 0.140741176 | 0.105368627 |
| 451 | /data5/data5/dongzhong/LymphGland/esophagus/2019-02-03/800811-15-15 - 2019-01-11 15.47.38.ndpi | 0 | 0.02330588 | 0 | 0 |
| 452 | /data5/data5/dongzhong/LymphGland/esophagus/2019-02-03/801225-18-18 - 2019-01-11 11.25.37.ndpi | 0 | 0.33657255 | 0.088133333 | 0.137411765 |
| 453 | /data5/data5/dongzhong/LymphGland/esophagus/2019-02-03/801343-20-20 - 2019-01-11 11.33.30.ndpi | 0 | 0.36469412 | 0.068435294 | 0.03807451 |
| 454 | /data5/data5/dongzhong/LymphGland/esophagus/2019-02-03/801625-11-11 - 2019-01-11 10.37.40.ndpi | 0 | 0.79837255 | 0.687309804 | 0.453368627 |
| 455 | /data5/data5/dongzhong/LymphGland/esophagus/2019-02-03/801768-2-2 - 2019-01-11 11.48.33.ndpi | 0 | 0.03679216 | 9.41176E-05 | 0.014541176 |
| 456 | /data5/data5/dongzhong/LymphGland/esophagus/2019-02-03/801804-9-9 - 2019-01-11 11.49.35.ndpi | 0 | 0.54183137 | 0.161466667 | 0.085639216 |
| 457 | /data5/data5/dongzhong/LymphGland/esophagus/2019-02-03/802277-13-13 - 2019-01-11 14.52.09.ndpi | 0 | 0.58007843 | 0.719988235 | 0.100003922 |
| 458 | /data5/data5/dongzhong/LymphGland/esophagus/2019-02-03/802465-15-15 - 2019-01-11 12.22.37.ndpi | 0 | 0.66647059 | 0.867737255 | 0.21294902 |
| 459 | /data5/data5/dongzhong/LymphGland/esophagus/2019-02-03/802501-33-33 - 2019-01-11 15.08.46.ndpi | 0 | 0.37309804 | 0.101509804 | 0.072039216 |
| 460 | /data5/data5/dongzhong/LymphGland/esophagus/2019-02-03/802624-8-8 - 2019-01-11 12.43.32.ndpi | 0 | 0.46680392 | 0.007584314 | 0.060560784 |
| 461 | /data5/data5/dongzhong/LymphGland/esophagus/2019-02-03/802629-16-16 - 2019-01-11 15.13.44.ndpi | 0 | 0.25035686 | 0.07427451 | 0.04547451 |
| 462 | /data5/data5/dongzhong/LymphGland/esophagus/2019-02-03/802630-31-31 - 2019-01-11 15.18.53.ndpi | 0 | 0.30672549 | 0.361713725 | 0.474152941 |
| 463 | /data5/data5/dongzhong/LymphGland/esophagus/2019-02-03/802679-18-18 - 2019-01-11 13.14.08.ndpi | 0 | 0.22553725 | 0.108698039 | 0.150203922 |
| 464 | /data5/data5/dongzhong/LymphGland/esophagus/2019-02-03/802855-13-13 - 2019-01-11 15.26.09.ndpi | 0 | 0.67748235 | 0.622647059 | 0.099113725 |
| 465 | /data5/data5/dongzhong/LymphGland/esophagus/2019-02-03/803307-15-15 - 2019-01-11 13.36.48.ndpi | 0 | 0.60295294 | 0.748258824 | 0.0924 |
| 466 | /data5/data5/dongzhong/LymphGland/esophagus/2019-02-03/803309-15-15 - 2019-01-11 15.36.30.ndpi | 0 | 0.13117647 | 0 | 0.005847059 |
| 467 | /data5/data5/dongzhong/LymphGland/esophagus/2019-02-03/803342-13-13 - 2019-01-11 13.50.00.ndpi | 0 | 0.23377255 | 0.270517647 | 0.208839216 |
| 468 | /data5/data5/dongzhong/LymphGland/esophagus/2019-02-03/803453-23-23 - 2019-01-11 14.04.44.ndpi | 0 | 0.26850196 | 0.052141176 | 0.10434902 |
| 469 | /data5/data5/dongzhong/LymphGland/esophagus/2019-02-03/803626-11-11 - 2019-01-11 14.27.32.ndpi | 0 | 0.56113333 | 0.433807843 | 0.230513725 |
| 470 | /data6/医科院肿瘤医院正常和其他/2018-11-09/淋巴（食管）正常/805556-13-0;1.ndpi | 0 | 0.04871373 | 0 | 0 |
| 471 | /data6/医科院肿瘤医院正常和其他/2018-08-24/淋巴（食道）正常/714556-22-0;1.tif | 0 | 0.19676863 | 0.061219608 | 0.212580392 |
| 472 | /data6/医科院肿瘤医院正常和其他/2018-08-24/淋巴（食道）正常/719365-23-0;1.tif | 0 | 0.04644314 | 0.058341176 | 0.004862745 |
| 473 | /data5/data5/dongzhong/LymphGland/esophagus/2018-12-28/2018-12-20_18.51.47.ndpi | 0 | 0.37301176 | 0.194258824 | 0.079184314 |
| 474 | /data5/data5/dongzhong/LymphGland/esophagus/2018-12-28/2018-12-20_18.56.12.ndpi | 0 | 0.29887843 | 0.001831373 | 0.029772549 |
| 475 | /data5/data5/dongzhong/LymphGland/esophagus/2018-12-28/2018-12-20_19.09.19.ndpi | 0 | 0.79856078 | 0.794482353 | 0.85667451 |
| 476 | /data5/data5/dongzhong/LymphGland/esophagus/2018-12-28/2018-12-24_12.31.37.ndpi | 0 | 0.4891451 | 0.02652549 | 0.028752941 |
| 477 | /data5/data5/dongzhong/LymphGland/esophagus/2018-12-28/2018-12-21_11.44.58.ndpi | 0 | 0.08008235 | 0 | 0.00034902 |
| 478 | /data6/医科院肿瘤医院正常和其他/2018-11-02/淋巴(食管)正常/789931-12-0;2.ndpi | 0 | 0.22430196 | 6.27451E-05 | 0.086262745 |
| 479 | /data6/医科院肿瘤医院正常和其他/2018-11-02/淋巴(食管)正常/790210-13-0;3.ndpi | 0 | 0.42237255 | 0.007576471 | 0.000870588 |
| 480 | /data5/data5/dongzhong/LymphGland/esophagus/2018-12-28/2018-12-21_09.33.57.ndpi | 0 | 0.41903529 | 0.702133333 | 0.080172549 |
| 481 | /data5/data5/dongzhong/LymphGland/esophagus/2018-12-28/2018-12-21_09.38.25.ndpi | 0 | 0.55252941 | 0.234764706 | 0.219298039 |
| 482 | /data5/data5/dongzhong/LymphGland/esophagus/2018-12-28/2018-12-21_09.47.12.ndpi | 0 | 0.65766667 | 0.59174902 | 0.240086275 |
| 483 | /data5/data5/dongzhong/LymphGland/esophagus/2018-12-28/2018-12-21_09.52.36.ndpi | 0 | 0.13090196 | 0 | 0.004137255 |
| 484 | /data5/data5/dongzhong/LymphGland/esophagus/2018-12-28/2018-12-21_10.04.50.ndpi | 0 | 0.12340784 | 0.000658824 | 0.059121569 |
| 485 | /data5/data5/dongzhong/LymphGland/esophagus/2018-12-28/2018-12-21_10.17.09.ndpi | 0 | 0.59072941 | 0.778698039 | 0.068627451 |
| 486 | /data5/data5/dongzhong/LymphGland/esophagus/2018-12-28/2018-12-21_10.27.31.ndpi | 0 | 0.41135686 | 0.016078431 | 0.027913725 |
| 487 | /data5/data5/dongzhong/LymphGland/esophagus/2018-12-28/2018-12-21_11.59.50.ndpi | 0 | 0.67357255 | 0.560152941 | 0.230423529 |
| 488 | /data5/data5/dongzhong/LymphGland/esophagus/2018-12-28/2018-12-21_12.00.56.ndpi | 0 | 0.64093333 | 0.007337255 | 0.355776471 |
| 489 | /data6/医科院肿瘤医院正常和其他/2018-11-02/淋巴(食管)正常/781405-23-0;5.ndpi | 0 | 0.61993333 | 0.002698039 | 3.13725E-05 |
| 490 | /data6/医科院肿瘤医院正常和其他/2018-11-02/淋巴(食管)正常/781530-16-0;5.ndpi | 0 | 0.24460784 | 0.003333333 | 3.52941E-05 |
| 491 | /data5/data5/dongzhong/LymphGland/esophagus/2018-12-28/2018-12-21_12.57.46.ndpi | 0 | 0.28416863 | 0.000121569 | 0.007231373 |
| 492 | /data6/医科院肿瘤医院正常和其他/2018-11-02/淋巴(食管)正常/782266-14-0;1.ndpi | 0 | 0.34564314 | 0 | 0 |
| 493 | /data5/data5/dongzhong/LymphGland/esophagus/2018-12-28/2018-12-21_13.11.09.ndpi | 0 | 0.91067843 | 0.527717647 | 0.169882353 |
| 494 | /data5/data5/dongzhong/LymphGland/esophagus/2018-12-28/2018-12-21_13.13.40.ndpi | 0 | 0.39247451 | 0.222184314 | 0.352027451 |
| 495 | /data5/data5/dongzhong/LymphGland/esophagus/2018-12-28/2018-12-27_15.58.29.ndpi | 0 | 0.63854118 | 0.838129412 | 0.202317647 |
| 496 | /data6/医科院肿瘤医院正常和其他/2018-11-02/淋巴(食管)正常/783372-17-0;3.ndpi | 0 | 0.1506 | 0.000839216 | 0 |
| 497 | /data5/data5/dongzhong/LymphGland/esophagus/2018-12-28/2018-12-21_13.32.29.ndpi | 0 | 0.5079098 | 0.788427451 | 0.117658824 |
| 498 | /data6/医科院肿瘤医院正常和其他/2018-11-02/淋巴(食管)正常/783371-13-0;4.ndpi | 0 | 0.17327843 | 5.4902E-05 | 0 |
| 499 | /data5/data5/dongzhong/LymphGland/esophagus/2018-12-20/797114-17-0;1.ndpi | 0 | 0.57368627 | 0.129588235 | 0.274152941 |
| 500 | /data5/data5/dongzhong/LymphGland/esophagus/2018-12-20/2018-12-19 18.15.04.ndpi | 0 | 0.21679216 | 0.050184314 | 0.129062745 |
| 501 | /data5/data5/dongzhong/LymphGland/esophagus/2018-12-20/2018-12-19 18.21.41.ndpi | 0 | 0.25512549 | 0.005509804 | 0.061796078 |
| 502 | /data5/data5/dongzhong/LymphGland/esophagus/2018-12-20/2018-12-19 18.31.11.ndpi | 0 | 0.11234902 | 0.000427451 | 0.029831373 |
| 503 | /data5/data5/dongzhong/LymphGland/esophagus/2018-12-20/2018-12-19 18.41.49.ndpi | 0 | 0.79778431 | 0.610627451 | 0.824117647 |
| 504 | /data6/医科院肿瘤医院正常和其他/2018-11-02/淋巴(食管)正常/785318-13-0;3.ndpi | 0 | 0.16989804 | 1.96078E-05 | 5.09804E-05 |
| 505 | /data5/data5/dongzhong/LymphGland/esophagus/2018-12-20/2018-12-19 18.47.48.ndpi | 0 | 0.30203922 | 0.004886275 | 0.134392157 |
| 506 | /data5/data5/dongzhong/LymphGland/esophagus/2018-12-20/2018-12-19 18.58.22.ndpi | 0 | 0.76637647 | 0.000129412 | 0.013137255 |
| 507 | /data5/data5/dongzhong/LymphGland/esophagus/2018-12-20/2018-12-19 19.02.19.ndpi | 0 | 0.03639216 | 7.84314E-05 | 0.116505882 |
| 508 | /data5/data5/dongzhong/LymphGland/esophagus/2018-12-20/2018-12-19 19.12.09.ndpi | 0 | 0.31137647 | 0.094933333 | 0.050235294 |
| 509 | /data5/data5/dongzhong/LymphGland/esophagus/2018-12-20/2018-12-19 19.19.25.ndpi | 0 | 0.93942353 | 0.614835294 | 0.97872549 |
| 510 | /data5/data5/dongzhong/LymphGland/esophagus/2018-12-20/2018-12-19 19.24.28.ndpi | 0 | 0.55125882 | 0.1404 | 0.033878431 |
| 511 | /data5/data5/dongzhong/LymphGland/esophagus/2018-12-20/2018-12-19 19.34.38.ndpi | 0 | 0.4560549 | 0.018639216 | 0.008419608 |
| 512 | /data5/data5/dongzhong/LymphGland/esophagus/2018-12-20/2018-12-19 19.48.47.ndpi | 0 | 0.75461961 | 0.870811765 | 0.798278431 |
| 513 | /data5/data5/dongzhong/LymphGland/esophagus/2018-12-20/2018-12-19 19.54.55.ndpi | 0 | 0.31177255 | 0.081505882 | 0.027501961 |
| 514 | /data5/data5/dongzhong/LymphGland/esophagus/2018-12-20/2018-12-19 19.58.17.ndpi | 0 | 0.39285098 | 0.06005098 | 0.052129412 |
| 515 | /data5/data5/dongzhong/LymphGland/esophagus/2018-12-20/2018-12-19 20.07.54.ndpi | 0 | 0.62780784 | 0.023266667 | 0.02405098 |
| 516 | /data5/data5/dongzhong/LymphGland/esophagus/2018-12-20/2018-12-20 14.49.33.ndpi | 0 | 0.87170196 | 0.002835294 | 0.046556863 |
| 517 | /data5/data5/dongzhong/LymphGland/esophagus/2018-12-20/2018-12-20 14.53.15.ndpi | 0 | 0.44374118 | 0.026486275 | 0.092086275 |
| 518 | /data5/data5/dongzhong/LymphGland/esophagus/2018-12-20/2018-12-20 15.00.07.ndpi | 0 | 0.26952549 | 0.04314902 | 0.05165098 |
| 519 | /data5/data5/dongzhong/LymphGland/esophagus/2018-12-20/2018-12-20 15.03.50.ndpi | 0 | 0.68551765 | 0.657231373 | 0.28785098 |
| 520 | /data5/data5/dongzhong/LymphGland/esophagus/2018-12-20/2018-12-20 15.09.44.ndpi | 0 | 0.57285098 | 0 | 0.188764706 |
| 521 | /data5/data5/dongzhong/LymphGland/esophagus/2018-12-20/790564-14-0;5.ndpi | 0 | 0.28174902 | 0.514003922 | 0.078129412 |
| 522 | /data5/data5/dongzhong/LymphGland/esophagus/2018-12-20/2018-12-20 15.19.09.ndpi | 0 | 0.0472 | 0 | 0 |
| 523 | /data5/data5/dongzhong/LymphGland/esophagus/2018-12-20/790573-21-0;5.ndpi | 0 | 0.19051373 | 0.124533333 | 0.10565098 |
| 524 | /data5/data5/dongzhong/LymphGland/esophagus/2018-12-20/791683-25-0;1.ndpi | 0 | 0.76001569 | 0.779266667 | 0.477188235 |
| 525 | /data5/data5/dongzhong/LymphGland/esophagus/2018-12-20/792225-17-0;2.ndpi | 0 | 0.59931373 | 0.423505882 | 0.070752941 |
| 526 | /data5/data5/dongzhong/LymphGland/esophagus/2018-12-20/792483-10-0;4.ndpi | 0 | 0.65343922 | 0.011482353 | 0.168611765 |
| 527 | /data5/data5/dongzhong/LymphGland/esophagus/2018-12-20/792915-16-0;6.ndpi | 0 | 0.4648549 | 0.124035294 | 0.028321569 |
| 528 | /data5/data5/dongzhong/LymphGland/esophagus/2018-12-20/793059-11-0;1.ndpi | 0 | 0.05826667 | 0 | 0.001847059 |
| 529 | /data5/data5/dongzhong/LymphGland/esophagus/2018-12-20/793167-14-0;1.ndpi | 0 | 0.37785098 | 0.008521569 | 0.030345098 |
| 530 | /data5/data5/dongzhong/LymphGland/esophagus/2018-12-20/793533-19-0;1.ndpi | 0 | 0.45980784 | 0.212219608 | 0.06687451 |
| 531 | /data5/data5/dongzhong/LymphGland/esophagus/2018-12-20/793834-21-0;1.ndpi | 0 | 0.40701176 | 0.001301961 | 0.009996078 |
| 532 | /data5/data5/dongzhong/LymphGland/esophagus/2018-12-20/794196-12-0;5.ndpi | 0 | 0.23765882 | 0.768729412 | 0.090141176 |
| 533 | /data5/data5/dongzhong/LymphGland/esophagus/2018-12-20/794683-22-0;2.ndpi | 0 | 0.6628 | 0.266835294 | 0.214403922 |
| 534 | /data6/医科院肿瘤医院正常和其他/2018-11-02/淋巴(食管)正常/794889-8-0;1.ndpi | 0 | 0.098 | 0.000364706 | 0 |
| 535 | /data5/data5/dongzhong/LymphGland/esophagus/2018-12-20/795608-18-0；6.ndpi | 0 | 0.46686275 | 0.428737255 | 0.052603922 |
| 536 | /data5/data5/dongzhong/LymphGland/esophagus/2018-12-20/795928-2-0；1.ndpi | 0 | 0.92305098 | 0.969929412 | 0.98705098 |
| 537 | /data6/医科院肿瘤医院正常和其他/2018-11-02/淋巴(食管)正常/795996-13-0;2.ndpi | 0 | 0.02343922 | 0.004658824 | 0.008866667 |
| 538 | /data5/data5/dongzhong/LymphGland/esophagus/2018-12-20/796186-13-0;2.ndpi | 0 | 0.43803529 | 0.027039216 | 0.284388235 |
| 539 | /data5/data5/dongzhong/LymphGland/esophagus/2018-12-20/796657-22-0;3.ndpi | 0 | 0.70487059 | 0.360713725 | 0.749392157 |
| 540 | /data5/data5/dongzhong/LymphGland/esophagus/2018-12-20/797267-1-0;3.ndpi | 0 | 0.55747059 | 0.499003922 | 0.105235294 |
| 541 | /data5/data5/dongzhong/LymphGland/esophagus/2018-12-20/797479-11-0;2.ndpi | 0 | 0.46882745 | 0.140458824 | 0.008831373 |
| 542 | /data5/data5/dongzhong/LymphGland/esophagus/2018-12-20/797701-15-0;5.ndpi | 0 | 0.55818431 | 0.369752941 | 0.04467451 |
| 543 | /data5/data5/dongzhong/LymphGland/esophagus/2018-12-20/798305-17-0；6.ndpi | 0 | 0.65492941 | 0.14847451 | 0.07592549 |
| 544 | /data5/data5/dongzhong/LymphGland/esophagus/2018-12-20/798347-16-0;2.ndpi | 0 | 0.46616471 | 0.579576471 | 0.008368627 |
| 545 | /data5/data5/dongzhong/LymphGland/esophagus/2018-12-20/798348-16-0；4.ndpi | 0 | 0.34312549 | 0.033631373 | 0.118976471 |
| 546 | /data5/data5/dongzhong/LymphGland/esophagus/2018-12-20/798363-23-0；4.ndpi | 0 | 0.2383451 | 0.041619608 | 0.381513725 |
| 547 | /data5/data5/dongzhong/LymphGland/esophagus/2018-12-20/798467-14-0；2.ndpi | 0 | 0.11744314 | 0.013156863 | 0.009760784 |
| 548 | /data5/data5/dongzhong/LymphGland/esophagus/2018-12-20/798539-32-0;5.ndpi | 0 | 0.22935686 | 0.399121569 | 0.017580392 |
| 549 | /data6/医科院肿瘤医院正常和其他/2018-11-09/淋巴（食管）正常/804579-16-0;5.ndpi | 0 | 0.92322745 | 0.221 | 9.41176E-05 |
| 550 | /data6/医科院肿瘤医院正常和其他/2018-11-09/淋巴（食管）正常/805750-20-0;2.ndpi | 0 | 0.14608235 | 0.003745098 | 5.4902E-05 |
| 551 | /data6/医科院肿瘤医院正常和其他/2018-11-09/淋巴（食管）正常/775705-19-0;3.ndpi | 0 | 0.5144 | 0.00034902 | 0 |
| 552 | /data6/医科院肿瘤医院正常和其他/2018-11-09/淋巴（食管）正常/775522-13-0;1.ndpi | 0 | 0.09843529 | 0.024227451 | 2.7451E-05 |
| 553 | /data6/医科院肿瘤医院正常和其他/2018-11-09/淋巴（食管）正常/773527-15-0;2.ndpi | 0 | 0.4695451 | 4.31373E-05 | 0 |
| 554 | /data6/医科院肿瘤医院正常和其他/2018-11-09/淋巴（食管）正常/773083-13-0;4.ndpi | 0 | 0.49283529 | 0.078588235 | 0 |
| 555 | /data6/医科院肿瘤医院正常和其他/2018-11-09/淋巴（食管）正常/771977-14-0;1.ndpi | 0 | 0.65392549 | 0.010901961 | 0.000486275 |
| 556 | /data6/医科院肿瘤医院正常和其他/2018-11-09/淋巴（食管）正常/768903-11-0;2.ndpi | 0 | 0.58762353 | 0.016541176 | 8.62745E-05 |
| 557 | /data6/医科院肿瘤医院正常和其他/2018-11-09/淋巴（食管）正常/768724-9-0;2.ndpi | 0 | 0.35099608 | 6.66667E-05 | 1.17647E-05 |
| 558 | /data6/医科院肿瘤医院正常和其他/2018-11-09/淋巴（食管）正常/768709-15-0;5.ndpi | 0 | 0.64750196 | 0.091047059 | 3.92157E-06 |
| 559 | /data5/data5/dongzhong/LymphGland/esophagus/2019-02-03/2018-12-27 19.34.09.ndpi | 0 | 0.14356863 | 0.001329412 | 0.013101961 |
| 560 | /data5/data5/dongzhong/LymphGland/esophagus/2019-02-03/2018-12-27 19.07.36.ndpi | 0 | 0.67902353 | 0.003588235 | 0.160764706 |
| 561 | /data6/医科院肿瘤医院正常和其他/2018-11-02/淋巴(食管)正常/776561-11-0；2.ndpi | 0 | 0.01943137 | 0 | 3.92157E-06 |
| 562 | /data6/医科院肿瘤医院正常和其他/2018-11-02/淋巴(食管)正常/776703-23-0;2.ndpi | 0 | 0.05698431 | 0 | 0 |
| 563 | /data6/医科院肿瘤医院正常和其他/2018-11-02/淋巴(食管)正常/777581-17-0;2.ndpi | 0 | 0.61186275 | 0.002137255 | 0.000192157 |
| 564 | /data6/医科院肿瘤医院正常和其他/2018-11-02/淋巴(食管)正常/778514-13-0;1.ndpi | 0 | 0.70948627 | 0.00192549 | 0 |
| 565 | /data5/data5/dongzhong/LymphGland/esophagus/2019-02-03/2018-12-27 18.58.33.ndpi | 0 | 0.17450196 | 0.01214902 | 0.096196078 |
| 566 | /data5/data5/dongzhong/LymphGland/esophagus/2019-02-03/2018-12-27 19.18.31.ndpi | 0 | 0.27318039 | 0.383647059 | 0.036882353 |
| 567 | /data5/data5/dongzhong/LymphGland/esophagus/2019-02-03/2018-12-27 19.21.48.ndpi | 0 | 0.38638039 | 0.483733333 | 0.091580392 |
| 568 | /data5/data5/dongzhong/LymphGland/esophagus/2019-02-03/2018-12-27 19.27.23.ndpi | 0 | 0.46638431 | 0.05834902 | 0.046988235 |
| 569 | /data5/data5/dongzhong/LymphGland/esophagus/2019-02-03/2018-12-27 19.49.56.ndpi | 0 | 0.74682745 | 0.218682353 | 0.358301961 |
| 570 | /data5/data5/dongzhong/LymphGland/esophagus/2019-02-03/2018-12-27 19.53.48.ndpi | 0 | 0.63322745 | 0.828788235 | 0.038423529 |
| 571 | /data5/data5/dongzhong/LymphGland/esophagus/2019-02-03/800190-19-19 - 2019-01-11 10.42.52.ndpi | 0 | 0.40607843 | 0.108207843 | 0.030737255 |
| 572 | /data5/data5/dongzhong/LymphGland/esophagus/2019-02-03/800368-16-16 - 2019-01-11 10.46.22.ndpi | 0 | 0.50661961 | 0.633901961 | 0.077047059 |
| 573 | /data5/data5/dongzhong/LymphGland/esophagus/2019-02-03/800400-33-33 - 2019-01-11 10.57.25.ndpi | 0 | 0.27166667 | 0.105894118 | 0.174011765 |
| 574 | /data5/data5/dongzhong/LymphGland/esophagus/2019-02-03/800645-16-16 - 2019-01-11 11.12.23.ndpi | 0 | 0.46487451 | 0.278062745 | 0.151603922 |
| 575 | /data5/data5/dongzhong/LymphGland/esophagus/2019-02-03/800811-12-12 - 2019-01-11 14.38.18.ndpi | 0 | 0.71489412 | 0.894796078 | 0.257764706 |
| 576 | /data5/data5/dongzhong/LymphGland/esophagus/2019-02-03/801225-12-12 - 2019-01-11 11.24.04.ndpi | 0 | 0.20218824 | 0.001901961 | 0.048760784 |
| 577 | /data5/data5/dongzhong/LymphGland/esophagus/2019-02-03/801343-20-20 - 2019-01-11 11.33.30.ndpi | 0 | 0.36469412 | 0.068435294 | 0.03807451 |
| 578 | /data5/data5/dongzhong/LymphGland/esophagus/2019-02-03/801625-11-11 - 2019-01-11 10.37.40.ndpi | 0 | 0.79837255 | 0.687309804 | 0.453368627 |
| 579 | /data5/data5/dongzhong/LymphGland/esophagus/2019-02-03/801768-2-2 - 2019-01-11 11.48.33.ndpi | 0 | 0.03679216 | 9.41176E-05 | 0.014541176 |
| 580 | /data5/data5/dongzhong/LymphGland/esophagus/2019-02-03/801804-19-19 - 2019-01-11 12.02.28.ndpi | 0 | 0.72267059 | 0.491835294 | 0.394137255 |
| 581 | /data5/data5/dongzhong/LymphGland/esophagus/2019-02-03/802277-20-20 - 2019-01-11 12.15.49.ndpi | 0 | 0.77273725 | 0.538901961 | 0.127031373 |
| 582 | /data5/data5/dongzhong/LymphGland/esophagus/2019-02-03/802465-15-15 - 2019-01-11 12.22.37.ndpi | 0 | 0.66647059 | 0.867737255 | 0.21294902 |
| 583 | /data5/data5/dongzhong/LymphGland/esophagus/2019-02-03/802501-26-26 - 2019-01-11 15.04.12.ndpi | 0 | 0.68065882 | 0.000847059 | 0.025913725 |
| 584 | /data5/data5/dongzhong/LymphGland/esophagus/2019-02-03/802624-8-8 - 2019-01-11 12.43.32.ndpi | 0 | 0.46680392 | 0.007584314 | 0.060560784 |
| 585 | /data5/data5/dongzhong/LymphGland/esophagus/2019-02-03/802629-19-19 - 2019-01-11 15.16.11.ndpi | 0 | 0.21589804 | 0.004282353 | 0.047701961 |
| 586 | /data5/data5/dongzhong/LymphGland/esophagus/2019-02-03/802630-29-29 - 2019-01-11 13.04.25.ndpi | 0 | 0.84054118 | 0.789160784 | 0.17394902 |
| 587 | /data5/data5/dongzhong/LymphGland/esophagus/2019-02-03/802679-18-18 - 2019-01-11 13.14.08.ndpi | 0 | 0.22553725 | 0.108698039 | 0.150203922 |
| 588 | /data5/data5/dongzhong/LymphGland/esophagus/2019-02-03/802855-18-18 - 2019-01-11 15.32.28.ndpi | 0 | 0.75272157 | 0.65974902 | 0.366439216 |
| 589 | /data5/data5/dongzhong/LymphGland/esophagus/2019-02-03/803307-11-11 - 2019-01-11 13.31.31.ndpi | 0 | 0.60978431 | 0.717819608 | 0.144886275 |
| 590 | /data5/data5/dongzhong/LymphGland/esophagus/2019-02-03/803309-13-13 - 2019-01-11 15.34.46.ndpi | 0 | 0.41663137 | 0.55512549 | 0.198741176 |
| 591 | /data5/data5/dongzhong/LymphGland/esophagus/2019-02-03/803342-12-12 - 2019-01-11 13.48.32.ndpi | 0 | 0.09000784 | 0.211662745 | 0.073419608 |
| 592 | /data5/data5/dongzhong/LymphGland/esophagus/2019-02-03/803453-24-24 - 2019-01-11 14.06.32.ndpi | 0 | 0.11355294 | 0.023482353 | 0.022768627 |
| 593 | /data5/data5/dongzhong/LymphGland/esophagus/2019-02-03/803626-11-11 - 2019-01-11 14.27.32.ndpi | 0 | 0.56113333 | 0.433807843 | 0.230513725 |
| 594 | /data6/医科院肿瘤医院正常和其他/2018-11-09/淋巴（食管）正常/805556-9-0;3.ndpi | 0 | 0.36172549 | 0 | 0 |
| 595 | /data6/医科院肿瘤医院正常和其他/2018-08-24/淋巴（食道）正常/714556-23-0;4.tif | 0 | 0.18790196 | 0.046792157 | 0.185588235 |
| 596 | /data6/医科院肿瘤医院正常和其他/2018-08-24/淋巴（食道）正常/719365-23-0;1.tif | 0 | 0.04644314 | 0.058341176 | 0.004862745 |
| 597 | /data5/data5/dongzhong/LymphGland/esophagus/2018-12-28/2018-12-20_18.50.03.ndpi | 0 | 0.0692 | 0.001792157 | 0.029211765 |
| 598 | /data5/data5/dongzhong/LymphGland/esophagus/2018-12-28/2018-12-21_10.54.11.ndpi | 0 | 0.43630588 | 0.019956863 | 0.24865098 |
| 599 | /data5/data5/dongzhong/LymphGland/esophagus/2018-12-28/2018-12-20_19.00.45.ndpi | 0 | 0.32176078 | 0.011015686 | 0.137843137 |
| 600 | /data5/data5/dongzhong/LymphGland/esophagus/2018-12-28/2018-12-20_19.15.35.ndpi | 0 | 0.42215686 | 0.314070588 | 0.093152941 |
| 601 | /data5/data5/dongzhong/LymphGland/esophagus/2018-12-28/2018-12-21_11.44.58.ndpi | 0 | 0.08008235 | 0 | 0.00034902 |
| 602 | /data5/data5/dongzhong/LymphGland/esophagus/2018-12-28/2018-12-24_12.35.47.ndpi | 0 | 0.66060784 | 0.193541176 | 0.183494118 |
| 603 | /data6/医科院肿瘤医院正常和其他/2018-11-02/淋巴(食管)正常/790210-14-0;2.ndpi | 0 | 0.30636078 | 0 | 0 |
| 604 | /data5/data5/dongzhong/LymphGland/esophagus/2018-12-28/2018-12-21_09.33.57.ndpi | 0 | 0.41903529 | 0.702133333 | 0.080172549 |
| 605 | /data5/data5/dongzhong/LymphGland/esophagus/2018-12-28/2018-12-21_09.38.59.ndpi | 0 | 0.36712941 | 0.1636 | 0.195109804 |
| 606 | /data5/data5/dongzhong/LymphGland/esophagus/2018-12-28/2018-12-21_09.41.01.ndpi | 0 | 0.84167451 | 0.862513725 | 0.152509804 |
| 607 | /data5/data5/dongzhong/LymphGland/esophagus/2018-12-28/2018-12-21_09.57.45.ndpi | 0 | 0.75770196 | 0.287431373 | 0.103003922 |
| 608 | /data5/data5/dongzhong/LymphGland/esophagus/2018-12-28/2018-12-21_10.11.47.ndpi | 0 | 0.50425098 | 0.578145098 | 0.187772549 |
| 609 | /data5/data5/dongzhong/LymphGland/esophagus/2018-12-28/2018-12-21_10.14.54.ndpi | 0 | 0.88414902 | 0.169254902 | 0.600698039 |
| 610 | /data5/data5/dongzhong/LymphGland/esophagus/2018-12-28/2018-12-21_10.30.04.ndpi | 0 | 0.10367059 | 0.003141176 | 0.010439216 |
| 611 | /data5/data5/dongzhong/LymphGland/esophagus/2018-12-28/2018-12-21_11.59.05.ndpi | 0 | 0.32323529 | 0.000552941 | 0.026027451 |
| 612 | /data5/data5/dongzhong/LymphGland/esophagus/2018-12-28/2018-12-21_12.01.37.ndpi | 0 | 0.71114118 | 0.132984314 | 0.6158 |
| 613 | /data6/医科院肿瘤医院正常和其他/2018-11-02/淋巴(食管)正常/781405-23-0;5.ndpi | 0 | 0.61993333 | 0.002698039 | 3.13725E-05 |
| 614 | /data5/data5/dongzhong/LymphGland/esophagus/2018-12-28/2018-12-21_12.35.08.ndpi | 0 | 0.06500392 | 0 | 0.016027451 |
| 615 | /data5/data5/dongzhong/LymphGland/esophagus/2018-12-28/2018-12-21_12.58.33.ndpi | 0 | 0.18036078 | 0.003223529 | 0.103117647 |
| 616 | /data5/data5/dongzhong/LymphGland/esophagus/2018-12-28/2018-12-21_13.04.14.ndpi | 0 | 0.21454902 | 0.028529412 | 0.076396078 |
| 617 | /data5/data5/dongzhong/LymphGland/esophagus/2018-12-28/2018-12-21_13.10.09.ndpi | 0 | 0.66126667 | 0.818635294 | 0.40412549 |
| 618 | /data5/data5/dongzhong/LymphGland/esophagus/2018-12-28/2018-12-27_15.53.47.ndpi | 0 | 0.37822745 | 0.449192157 | 0.264090196 |
| 619 | /data5/data5/dongzhong/LymphGland/esophagus/2018-12-28/2018-12-27_16.00.49.ndpi | 0 | 0.34943922 | 0.131152941 | 0.038278431 |
| 620 | /data5/data5/dongzhong/LymphGland/esophagus/2019-02-03/2018-12-27 18.49.55.ndpi | 0 | 0.63452941 | 0.853811765 | 0.647227451 |
| 621 | /data5/data5/dongzhong/LymphGland/esophagus/2018-12-28/2018-12-21_13.37.09.ndpi | 0 | 0.29271765 | 0.124403922 | 0.120082353 |
| 622 | /data6/医科院肿瘤医院正常和其他/2018-11-02/淋巴(食管)正常/783371-9-0;1.ndpi | 0 | 0.74376863 | 0.317921569 | 0.000203922 |
| 623 | /data5/data5/dongzhong/LymphGland/esophagus/2018-12-20/797114-15-0;4.ndpi | 0 | 0.48041961 | 0.00474902 | 0.018082353 |
| 624 | /data5/data5/dongzhong/LymphGland/esophagus/2018-12-20/2018-12-19 18.15.51.ndpi | 0 | 0.16287059 | 0.046933333 | 0.039411765 |
| 625 | /data5/data5/dongzhong/LymphGland/esophagus/2018-12-20/2018-12-19 18.23.54.ndpi | 0 | 0.11907843 | 0 | 0.001239216 |
| 626 | /data5/data5/dongzhong/LymphGland/esophagus/2018-12-20/2018-12-19 18.30.22.ndpi | 0 | 0.87200392 | 0.961823529 | 0.064435294 |
| 627 | /data5/data5/dongzhong/LymphGland/esophagus/2018-12-20/2018-12-19 18.39.35.ndpi | 0 | 0.45864314 | 0.175368627 | 0.156329412 |
| 628 | /data5/data5/dongzhong/LymphGland/esophagus/2018-12-20/2018-12-19 18.43.23.ndpi | 0 | 0.87112549 | 0.973403922 | 0.964141176 |
| 629 | /data5/data5/dongzhong/LymphGland/esophagus/2018-12-20/2018-12-20 13.02.06.ndpi | 0 | 0.04515686 | 0 | 0.002831373 |
| 630 | /data5/data5/dongzhong/LymphGland/esophagus/2018-12-20/2018-12-19 18.59.13.ndpi | 0 | 0.81090196 | 0.093223529 | 0.357823529 |
| 631 | /data5/data5/dongzhong/LymphGland/esophagus/2018-12-20/2018-12-20 13.06.27.ndpi | 0 | 0.1127451 | 0.000137255 | 0.143039216 |
| 632 | /data5/data5/dongzhong/LymphGland/esophagus/2018-12-20/2018-12-19 19.11.43.ndpi | 0 | 0.73467843 | 0.446603922 | 0.034117647 |
| 633 | /data5/data5/dongzhong/LymphGland/esophagus/2018-12-20/2018-12-19 19.20.37.ndpi | 0 | 0.15594118 | 0.421270588 | 0.116254902 |
| 634 | /data5/data5/dongzhong/LymphGland/esophagus/2018-12-20/2018-12-19 19.23.35.ndpi | 0 | 0.3479098 | 0.011411765 | 0.008070588 |
| 635 | /data5/data5/dongzhong/LymphGland/esophagus/2018-12-20/2018-12-19 19.34.38.ndpi | 0 | 0.4560549 | 0.018639216 | 0.008419608 |
| 636 | /data5/data5/dongzhong/LymphGland/esophagus/2018-12-20/2018-12-19 19.42.58.ndpi | 0 | 0.33187451 | 0.002988235 | 0.071262745 |
| 637 | /data5/data5/dongzhong/LymphGland/esophagus/2018-12-20/2018-12-19 19.57.24.ndpi | 0 | 0.70209412 | 0.558305882 | 0.285443137 |
| 638 | /data5/data5/dongzhong/LymphGland/esophagus/2018-12-20/2018-12-19 19.59.17.ndpi | 0 | 0.23856078 | 0.010231373 | 0.017317647 |
| 639 | /data5/data5/dongzhong/LymphGland/esophagus/2018-12-20/2018-12-19 20.05.28.ndpi | 0 | 0.21071765 | 0.000215686 | 0.17274902 |
| 640 | /data5/data5/dongzhong/LymphGland/esophagus/2018-12-20/2018-12-20 14.44.29.ndpi | 0 | 0.87388235 | 0.980698039 | 0.968513725 |
| 641 | /data5/data5/dongzhong/LymphGland/esophagus/2018-12-20/2018-12-20 14.53.15.ndpi | 0 | 0.44374118 | 0.026486275 | 0.092086275 |
| 642 | /data5/data5/dongzhong/LymphGland/esophagus/2018-12-20/2018-12-20 15.00.07.ndpi | 0 | 0.26952549 | 0.04314902 | 0.05165098 |
| 643 | /data5/data5/dongzhong/LymphGland/esophagus/2018-12-20/2018-12-20 15.47.21.ndpi | 0 | 0.44010196 | 0.317054902 | 0.132627451 |
| 644 | /data5/data5/dongzhong/LymphGland/esophagus/2018-12-20/2018-12-20 15.06.40.ndpi | 0 | 0.51838039 | 0.560047059 | 0.060768627 |
| 645 | /data5/data5/dongzhong/LymphGland/esophagus/2018-12-20/790564-20-0;12.ndpi | 0 | 0.32552157 | 0.214870588 | 0.184541176 |
| 646 | /data5/data5/dongzhong/LymphGland/esophagus/2018-12-20/2018-12-20 15.19.09.ndpi | 0 | 0.0472 | 0 | 0 |
| 647 | /data5/data5/dongzhong/LymphGland/esophagus/2018-12-20/790573-20-0;1.ndpi | 0 | 0.31783137 | 0.320411765 | 0.08492549 |
| 648 | /data6/医科院肿瘤医院正常和其他/2018-11-02/淋巴(食管)正常/791683-15-0;3.ndpi | 0 | 0.25673725 | 0.001039216 | 4.70588E-05 |
| 649 | /data5/data5/dongzhong/LymphGland/esophagus/2018-12-20/792225-12-0;3.ndpi | 0 | 0.77199216 | 0.103423529 | 0.115972549 |
| 650 | /data5/data5/dongzhong/LymphGland/esophagus/2018-12-20/792483-11-0;4.ndpi | 0 | 0.38336863 | 0.064164706 | 0.060039216 |
| 651 | /data5/data5/dongzhong/LymphGland/esophagus/2018-12-20/792915-10-0;4.ndpi | 0 | 0.33486275 | 0.211494118 | 0.216392157 |
| 652 | /data5/data5/dongzhong/LymphGland/esophagus/2018-12-20/793059-10-0;2.ndpi | 0 | 0.2371451 | 0.0076 | 0.01305098 |
| 653 | /data5/data5/dongzhong/LymphGland/esophagus/2018-12-20/793167-14-0;1.ndpi | 0 | 0.37785098 | 0.008521569 | 0.030345098 |
| 654 | /data5/data5/dongzhong/LymphGland/esophagus/2018-12-20/793533-17-0;1.ndpi | 0 | 0.04261569 | 0 | 0.002988235 |
| 655 | /data5/data5/dongzhong/LymphGland/esophagus/2018-12-20/793834-20-0;3.ndpi | 0 | 0.55958039 | 0.140792157 | 0.404623529 |
| 656 | /data5/data5/dongzhong/LymphGland/esophagus/2018-12-20/794196-13-0;4.ndpi | 0 | 0.51913725 | 0.820094118 | 0.024556863 |
| 657 | /data5/data5/dongzhong/LymphGland/esophagus/2018-12-20/794683-21-0;3.ndpi | 0 | 0.49619608 | 0.317447059 | 0.065972549 |
| 658 | /data5/data5/dongzhong/LymphGland/esophagus/2018-12-20/794889-18-0;1.ndpi | 0 | 0.25248235 | 0.041372549 | 0.21387451 |
| 659 | /data5/data5/dongzhong/LymphGland/esophagus/2018-12-20/795608-17-0；5.ndpi | 0 | 0.61123922 | 0.263011765 | 0.111945098 |
| 660 | /data5/data5/dongzhong/LymphGland/esophagus/2018-12-20/795928-2-0；1.ndpi | 0 | 0.92305098 | 0.969929412 | 0.98705098 |
| 661 | /data6/医科院肿瘤医院正常和其他/2018-11-02/淋巴(食管)正常/795996-13-0;2.ndpi | 0 | 0.02343922 | 0.004658824 | 0.008866667 |
| 662 | /data5/data5/dongzhong/LymphGland/esophagus/2018-12-20/796186-13-0;2.ndpi | 0 | 0.43803529 | 0.027039216 | 0.284388235 |
| 663 | /data5/data5/dongzhong/LymphGland/esophagus/2018-12-20/796657-23-0;3.ndpi | 0 | 0.41260392 | 0.398023529 | 0.189980392 |
| 664 | /data5/data5/dongzhong/LymphGland/esophagus/2018-12-20/797267-3-0;2.ndpi | 0 | 0.17970588 | 0 | 2.35294E-05 |
| 665 | /data5/data5/dongzhong/LymphGland/esophagus/2018-12-20/797479-17-0;2.ndpi | 0 | 0.34335686 | 0.030254902 | 0.03867451 |
| 666 | /data5/data5/dongzhong/LymphGland/esophagus/2018-12-20/797701-15-0;5.ndpi | 0 | 0.55818431 | 0.369752941 | 0.04467451 |
| 667 | /data5/data5/dongzhong/LymphGland/esophagus/2018-12-20/798305-14-0;1.ndpi | 0 | 0.64217647 | 0.732090196 | 0.329956863 |
| 668 | /data5/data5/dongzhong/LymphGland/esophagus/2018-12-20/798347-7-0;1.ndpi | 0 | 0.60931765 | 0.493392157 | 0.736301961 |
| 669 | /data5/data5/dongzhong/LymphGland/esophagus/2018-12-20/798348-19-0；2.ndpi | 0 | 0.88883137 | 0.219945098 | 0.027984314 |
| 670 | /data5/data5/dongzhong/LymphGland/esophagus/2018-12-20/798363-18-0；1.ndpi | 0 | 0.26794902 | 0.002294118 | 0.012082353 |
| 671 | /data5/data5/dongzhong/LymphGland/esophagus/2018-12-20/798467-18-0；2.ndpi | 0 | 0.00553725 | 0 | 0.000396078 |
| 672 | /data5/data5/dongzhong/LymphGland/esophagus/2018-12-20/798539-25-0;1.ndpi | 0 | 0.32079608 | 0.492043137 | 0.046623529 |
| 673 | /data6/医科院肿瘤医院正常和其他/2018-11-09/淋巴（食管）正常/804579-9-0;2.ndpi | 0 | 0.66381176 | 0.000576471 | 0 |
| 674 | /data6/医科院肿瘤医院正常和其他/2018-11-09/淋巴（食管）正常/805750-21-0;3.ndpi | 0 | 0.03663922 | 0 | 0 |
| 675 | /data6/医科院肿瘤医院正常和其他/2018-11-09/淋巴（食管）正常/775705-17-0;3.ndpi | 0 | 0.69167059 | 0.233396078 | 2.7451E-05 |
| 676 | /data6/医科院肿瘤医院正常和其他/2018-11-09/淋巴（食管）正常/775522-14-0;1.ndpi | 0 | 0.51938824 | 9.01961E-05 | 0.001972549 |
| 677 | /data6/医科院肿瘤医院正常和其他/2018-11-09/淋巴（食管）正常/773527-14-0;7.ndpi | 0 | 0.47691373 | 0.01892549 | 1.56863E-05 |
| 678 | /data6/医科院肿瘤医院正常和其他/2018-11-09/淋巴（食管）正常/773083-10-0;1.ndpi | 0 | 0.29952157 | 0.00165098 | 1.96078E-05 |
| 679 | /data6/医科院肿瘤医院正常和其他/2018-11-09/淋巴（食管）正常/771977-14-0;1.ndpi | 0 | 0.65392549 | 0.010901961 | 0.000486275 |
| 680 | /data6/医科院肿瘤医院正常和其他/2018-11-09/淋巴（食管）正常/768903-11-0;2.ndpi | 0 | 0.58762353 | 0.016541176 | 8.62745E-05 |
| 681 | /data6/医科院肿瘤医院正常和其他/2018-11-09/淋巴（食管）正常/768724-9-0;2.ndpi | 0 | 0.35099608 | 6.66667E-05 | 1.17647E-05 |
| 682 | /data6/医科院肿瘤医院正常和其他/2018-11-09/淋巴（食管）正常/768709-17-0;4.ndpi | 0 | 0.27366275 | 0.011513725 | 0 |
| 683 | /data5/data5/dongzhong/LymphGland/esophagus/2019-02-03/2018-12-27 19.41.16.ndpi | 0 | 0.6928549 | 0.205070588 | 0.371972549 |
| 684 | /data6/医科院肿瘤医院正常和其他/2018-11-09/淋巴（食管）正常/783685-15-0;5.ndpi | 0 | 0.19816471 | 0.006658824 | 9.80392E-05 |
| 685 | /data6/医科院肿瘤医院正常和其他/2018-11-02/淋巴(食管)正常/776561-12-0；1.ndpi | 0 | 0.0584549 | 0 | 0 |
| 686 | /data6/医科院肿瘤医院正常和其他/2018-11-02/淋巴(食管)正常/776703-21-0;3.ndpi | 0 | 0.3238549 | 0.001509804 | 0 |
| 687 | /data6/医科院肿瘤医院正常和其他/2018-11-02/淋巴(食管)正常/777581-18-0;4.ndpi | 0 | 0.34555294 | 0.027588235 | 0 |
| 688 | /data6/医科院肿瘤医院正常和其他/2018-11-02/淋巴(食管)正常/778514-14-0;1.ndpi | 0 | 0.76612941 | 0.002717647 | 0 |
| 689 | /data5/data5/dongzhong/LymphGland/esophagus/2019-02-03/2018-12-27 18.59.27.ndpi | 0 | 0.52630196 | 0.410443137 | 0.379882353 |
| 690 | /data5/data5/dongzhong/LymphGland/esophagus/2019-02-03/2018-12-27 19.13.18.ndpi | 0 | 0.75206667 | 0.184462745 | 0.07705098 |
| 691 | /data5/data5/dongzhong/LymphGland/esophagus/2019-02-03/2018-12-27 19.20.54.ndpi | 0 | 0.28206667 | 0.390819608 | 0.07234902 |
| 692 | /data5/data5/dongzhong/LymphGland/esophagus/2019-02-03/2018-12-27 19.31.08.ndpi | 0 | 0.44837647 | 0.269113725 | 0.134843137 |
| 693 | /data5/data5/dongzhong/LymphGland/esophagus/2019-02-03/2018-12-27 19.47.50.ndpi | 0 | 0.36119608 | 0.046643137 | 0.303898039 |
| 694 | /data5/data5/dongzhong/LymphGland/esophagus/2019-02-03/2018-12-27 19.53.22.ndpi | 0 | 0.34141569 | 0.517090196 | 0.055411765 |
| 695 | /data5/data5/dongzhong/LymphGland/esophagus/2019-02-03/800190-21-21 - 2019-01-11 10.45.36.ndpi | 0 | 0.22764706 | 1.96078E-05 | 0.015015686 |
| 696 | /data5/data5/dongzhong/LymphGland/esophagus/2019-02-03/800368-20-20 - 2019-01-11 10.50.56.ndpi | 0 | 0.36957647 | 0.05372549 | 0.053294118 |
| 697 | /data5/data5/dongzhong/LymphGland/esophagus/2019-02-03/800400-7-7 - 2019-01-11 10.53.33.ndpi | 0 | 0.81554902 | 0.764968627 | 0.885607843 |
| 698 | /data5/data5/dongzhong/LymphGland/esophagus/2019-02-03/800645-16-16 - 2019-01-11 11.12.23.ndpi | 0 | 0.46487451 | 0.278062745 | 0.151603922 |
| 699 | /data5/data5/dongzhong/LymphGland/esophagus/2019-02-03/800811-14-14 - 2019-01-11 11.21.44.ndpi | 0 | 0.30890588 | 0.044470588 | 0.023270588 |
| 700 | /data5/data5/dongzhong/LymphGland/esophagus/2019-02-03/801225-19-19 - 2019-01-11 11.27.26.ndpi | 0 | 0.32914902 | 0.249984314 | 0.175737255 |
| 701 | /data5/data5/dongzhong/LymphGland/esophagus/2019-02-03/801343-18-18 - 2019-01-11 11.30.22.ndpi | 0 | 0.28509412 | 0.430819608 | 0.076721569 |
| 702 | /data5/data5/dongzhong/LymphGland/esophagus/2019-02-03/801625-14-14 - 2019-01-11 14.46.52.ndpi | 0 | 0.28720784 | 0.195956863 | 0.036313725 |
| 703 | /data5/data5/dongzhong/LymphGland/esophagus/2019-02-03/801768-2-2 - 2019-01-11 11.48.33.ndpi | 0 | 0.03679216 | 9.41176E-05 | 0.014541176 |
| 704 | /data5/data5/dongzhong/LymphGland/esophagus/2019-02-03/801804-21-21 - 2019-01-11 12.03.52.ndpi | 0 | 0.571 | 0.068388235 | 0.389043137 |
| 705 | /data5/data5/dongzhong/LymphGland/esophagus/2019-02-03/802277-13-13 - 2019-01-11 14.52.09.ndpi | 0 | 0.58007843 | 0.719988235 | 0.100003922 |
| 706 | /data5/data5/dongzhong/LymphGland/esophagus/2019-02-03/802465-22-22 - 2019-01-11 12.28.29.ndpi | 0 | 0.45570196 | 0.009945098 | 0.000466667 |
| 707 | /data5/data5/dongzhong/LymphGland/esophagus/2019-02-03/802501-22-22 - 2019-01-11 12.37.06.ndpi | 0 | 0.3911098 | 0.023756863 | 0.251062745 |
| 708 | /data5/data5/dongzhong/LymphGland/esophagus/2019-02-03/802624-16-16 - 2019-01-11 15.12.15.ndpi | 0 | 0.58150196 | 0.035403922 | 0.188929412 |
| 709 | /data5/data5/dongzhong/LymphGland/esophagus/2019-02-03/802629-18-18 - 2019-01-11 15.15.10.ndpi | 0 | 0.21385098 | 0.004290196 | 0.247345098 |
| 710 | /data5/data5/dongzhong/LymphGland/esophagus/2019-02-03/802630-31-31 - 2019-01-11 15.18.53.ndpi | 0 | 0.30672549 | 0.361713725 | 0.474152941 |
| 711 | /data5/data5/dongzhong/LymphGland/esophagus/2019-02-03/802679-14-14 - 2019-01-11 13.07.59.ndpi | 0 | 0.12441569 | 0.036847059 | 0.084223529 |
| 712 | /data5/data5/dongzhong/LymphGland/esophagus/2019-02-03/802855-18-18 - 2019-01-11 15.32.28.ndpi | 0 | 0.75272157 | 0.65974902 | 0.366439216 |
| 713 | /data5/data5/dongzhong/LymphGland/esophagus/2019-02-03/803307-11-11 - 2019-01-11 13.31.31.ndpi | 0 | 0.60978431 | 0.717819608 | 0.144886275 |
| 714 | /data5/data5/dongzhong/LymphGland/esophagus/2019-02-03/803309-16-16 - 2019-01-11 15.36.59.ndpi | 0 | 0.23241961 | 0.000145098 | 0 |
| 715 | /data5/data5/dongzhong/LymphGland/esophagus/2019-02-03/803342-14-14 - 2019-01-11 13.51.42.ndpi | 0 | 0.14640392 | 0.290247059 | 0.902482353 |
| 716 | /data5/data5/dongzhong/LymphGland/esophagus/2019-02-03/803453-21-21 - 2019-01-11 14.01.04.ndpi | 0 | 0.3212902 | 0.37805098 | 0.161333333 |
| 717 | /data5/data5/dongzhong/LymphGland/esophagus/2019-02-03/803626-15-15 - 2019-01-11 15.45.55.ndpi | 0 | 0.76767843 | 0.453156863 | 0.048368627 |
| 718 | /data6/医科院肿瘤医院正常和其他/2018-11-09/淋巴（食管）正常/805556-12-0;6.ndpi | 0 | 0.14613725 | 0 | 2.35294E-05 |
| 719 | /data6/医科院肿瘤医院正常和其他/2018-08-24/淋巴（食道）正常/714556-23-0;4.tif | 0 | 0.18790196 | 0.046792157 | 0.185588235 |
| 720 | /data6/医科院肿瘤医院正常和其他/2018-08-24/淋巴（食道）正常/719365-23-0;1.tif | 0 | 0.04644314 | 0.058341176 | 0.004862745 |
| 721 | /data5/data5/dongzhong/LymphGland/esophagus/2018-12-28/2018-12-20_18.52.43.ndpi | 0 | 0.21441961 | 0.16652549 | 0.04747451 |
| 722 | /data5/data5/dongzhong/LymphGland/esophagus/2018-12-28/2018-12-21_10.54.11.ndpi | 0 | 0.43630588 | 0.019956863 | 0.24865098 |
| 723 | /data6/医科院肿瘤医院正常和其他/2018-11-09/淋巴（食管）正常/789260-14-0;5.ndpi | 0 | 0.53127451 | 0.019133333 | 0.000145098 |
| 724 | /data5/data5/dongzhong/LymphGland/esophagus/2018-12-28/2018-12-20_19.17.46.ndpi | 0 | 0.40496078 | 0.725070588 | 0.152619608 |
| 725 | /data5/data5/dongzhong/LymphGland/esophagus/2018-12-28/2018-12-20_19.19.22.ndpi | 0 | 0.54059608 | 0.377278431 | 0.076313725 |
| 726 | /data5/data5/dongzhong/LymphGland/esophagus/2018-12-28/2018-12-21_09.26.36.ndpi | 0 | 0.44911373 | 0.293027451 | 0.144647059 |
| 727 | /data6/医科院肿瘤医院正常和其他/2018-11-02/淋巴(食管)正常/790210-14-0;2.ndpi | 0 | 0.30636078 | 0 | 0 |
| 728 | /data5/data5/dongzhong/LymphGland/esophagus/2018-12-28/2018-12-21_09.34.50.ndpi | 0 | 0.70813333 | 0.376031373 | 0.131494118 |
| 729 | /data5/data5/dongzhong/LymphGland/esophagus/2018-12-28/2018-12-21_09.38.59.ndpi | 0 | 0.36712941 | 0.1636 | 0.195109804 |
| 730 | /data5/data5/dongzhong/LymphGland/esophagus/2018-12-28/2018-12-21_09.41.01.ndpi | 0 | 0.84167451 | 0.862513725 | 0.152509804 |
| 731 | /data5/data5/dongzhong/LymphGland/esophagus/2018-12-28/2018-12-21_09.57.45.ndpi | 0 | 0.75770196 | 0.287431373 | 0.103003922 |
| 732 | /data5/data5/dongzhong/LymphGland/esophagus/2018-12-28/2018-12-21_10.12.53.ndpi | 0 | 0.54810588 | 0.515301961 | 0.192160784 |
| 733 | /data5/data5/dongzhong/LymphGland/esophagus/2018-12-28/2018-12-21_10.14.54.ndpi | 0 | 0.88414902 | 0.169254902 | 0.600698039 |
| 734 | /data5/data5/dongzhong/LymphGland/esophagus/2018-12-28/2018-12-21_10.30.04.ndpi | 0 | 0.10367059 | 0.003141176 | 0.010439216 |
| 735 | /data5/data5/dongzhong/LymphGland/esophagus/2018-12-28/2018-12-27_16.14.19.ndpi | 0 | 0.88220784 | 0.949231373 | 0.961937255 |
| 736 | /data5/data5/dongzhong/LymphGland/esophagus/2018-12-28/2018-12-21_12.00.56.ndpi | 0 | 0.64093333 | 0.007337255 | 0.355776471 |
| 737 | /data6/医科院肿瘤医院正常和其他/2018-11-02/淋巴(食管)正常/781405-14-0;2.ndpi | 0 | 0.73804706 | 0.003341176 | 0 |
| 738 | /data5/data5/dongzhong/LymphGland/esophagus/2018-12-28/2018-12-21_12.35.08.ndpi | 0 | 0.06500392 | 0 | 0.016027451 |
| 739 | /data5/data5/dongzhong/LymphGland/esophagus/2018-12-28/2018-12-21_12.48.23.ndpi | 0 | 0.75852157 | 0.960682353 | 0.742843137 |
| 740 | /data5/data5/dongzhong/LymphGland/esophagus/2018-12-28/2018-12-24_15.38.25.ndpi | 0 | 0.53496078 | 0.170615686 | 0.303847059 |
| 741 | /data5/data5/dongzhong/LymphGland/esophagus/2018-12-28/2018-12-21_13.10.09.ndpi | 0 | 0.66126667 | 0.818635294 | 0.40412549 |
| 742 | /data5/data5/dongzhong/LymphGland/esophagus/2018-12-28/2018-12-27_15.52.14.ndpi | 0 | 0.49777255 | 0.596062745 | 0.251466667 |
| 743 | /data5/data5/dongzhong/LymphGland/esophagus/2018-12-28/2018-12-27_16.00.49.ndpi | 0 | 0.34943922 | 0.131152941 | 0.038278431 |
| 744 | /data6/医科院肿瘤医院正常和其他/2018-11-02/淋巴(食管)正常/783372-18-0;3.ndpi | 0 | 0.40900392 | 0.014462745 | 5.88235E-05 |
| 745 | /data5/data5/dongzhong/LymphGland/esophagus/2019-02-03/2018-12-27 18.51.17.ndpi | 0 | 0.44865098 | 0.008666667 | 0.032592157 |
| 746 | /data6/医科院肿瘤医院正常和其他/2018-11-02/淋巴(食管)正常/783371-13-0;4.ndpi | 0 | 0.17327843 | 5.4902E-05 | 0 |
| 747 | /data5/data5/dongzhong/LymphGland/esophagus/2018-12-20/797114-10-0;2.ndpi | 0 | 0.55088235 | 0.491054902 | 0.041576471 |
| 748 | /data5/data5/dongzhong/LymphGland/esophagus/2018-12-20/2018-12-19 18.15.51.ndpi | 0 | 0.16287059 | 0.046933333 | 0.039411765 |
| 749 | /data5/data5/dongzhong/LymphGland/esophagus/2018-12-20/2018-12-19 18.24.37.ndpi | 0 | 0.40596078 | 0.419509804 | 0.098145098 |
| 750 | /data5/data5/dongzhong/LymphGland/esophagus/2018-12-20/2018-12-19 18.30.22.ndpi | 0 | 0.87200392 | 0.961823529 | 0.064435294 |
| 751 | /data5/data5/dongzhong/LymphGland/esophagus/2018-12-20/2018-12-19 18.33.25.ndpi | 0 | 0.50293725 | 0.319835294 | 0.499458824 |
| 752 | /data5/data5/dongzhong/LymphGland/esophagus/2018-12-20/2018-12-19 18.43.23.ndpi | 0 | 0.87112549 | 0.973403922 | 0.964141176 |
| 753 | /data5/data5/dongzhong/LymphGland/esophagus/2018-12-20/2018-12-20 13.00.34.ndpi | 0 | 0.13696078 | 0.003098039 | 0.031654902 |
| 754 | /data5/data5/dongzhong/LymphGland/esophagus/2018-12-20/2018-12-19 18.58.22.ndpi | 0 | 0.76637647 | 0.000129412 | 0.013137255 |
| 755 | /data5/data5/dongzhong/LymphGland/esophagus/2018-12-20/2018-12-20 13.06.27.ndpi | 0 | 0.1127451 | 0.000137255 | 0.143039216 |
| 756 | /data5/data5/dongzhong/LymphGland/esophagus/2018-12-20/2018-12-19 19.09.37.ndpi | 0 | 0.34394118 | 0.497152941 | 0.024996078 |
| 757 | /data5/data5/dongzhong/LymphGland/esophagus/2018-12-20/2018-12-19 19.18.19.ndpi | 0 | 0.37160784 | 0.082580392 | 0.232545098 |
| 758 | /data5/data5/dongzhong/LymphGland/esophagus/2018-12-20/2018-12-19 19.23.35.ndpi | 0 | 0.3479098 | 0.011411765 | 0.008070588 |
| 759 | /data6/医科院肿瘤医院正常和其他/2018-11-02/淋巴(食管)正常/786098-10-0;6.ndpi | 0 | 0.36348627 | 0.001376471 | 0 |
| 760 | /data5/data5/dongzhong/LymphGland/esophagus/2018-12-20/2018-12-19 19.41.12.ndpi | 0 | 0.62337255 | 0.071113725 | 0.104333333 |
| 761 | /data5/data5/dongzhong/LymphGland/esophagus/2018-12-20/2018-12-19 19.57.24.ndpi | 0 | 0.70209412 | 0.558305882 | 0.285443137 |
| 762 | /data5/data5/dongzhong/LymphGland/esophagus/2018-12-20/2018-12-19 19.58.17.ndpi | 0 | 0.39285098 | 0.06005098 | 0.052129412 |
| 763 | /data5/data5/dongzhong/LymphGland/esophagus/2018-12-20/2018-12-19 20.06.15.ndpi | 0 | 0.85683922 | 0.761168627 | 0.496690196 |
| 764 | /data5/data5/dongzhong/LymphGland/esophagus/2018-12-20/2018-12-20 15.30.58.ndpi | 0 | 0.85632157 | 0.075431373 | 0.102360784 |
| 765 | /data6/医科院肿瘤医院正常和其他/2018-11-09/淋巴（食管）正常/787945-18-0;5.ndpi | 0 | 0.44070196 | 0.008372549 | 7.45098E-05 |
| 766 | /data5/data5/dongzhong/LymphGland/esophagus/2018-12-20/2018-12-20 15.00.07.ndpi | 0 | 0.26952549 | 0.04314902 | 0.05165098 |
| 767 | /data5/data5/dongzhong/LymphGland/esophagus/2018-12-20/2018-12-20 15.47.21.ndpi | 0 | 0.44010196 | 0.317054902 | 0.132627451 |
| 768 | /data5/data5/dongzhong/LymphGland/esophagus/2018-12-20/2018-12-20 15.09.13.ndpi | 0 | 0.0194549 | 0 | 0.000454902 |
| 769 | /data5/data5/dongzhong/LymphGland/esophagus/2018-12-20/790564-18-0;3.ndpi | 0 | 0.64609804 | 0.093141176 | 0.407031373 |
| 770 | /data5/data5/dongzhong/LymphGland/esophagus/2018-12-20/2018-12-20 15.17.22.ndpi | 0 | 0.73939608 | 0.238894118 | 0.340686275 |
| 771 | /data5/data5/dongzhong/LymphGland/esophagus/2018-12-20/790573-21-0;5.ndpi | 0 | 0.19051373 | 0.124533333 | 0.10565098 |
| 772 | /data5/data5/dongzhong/LymphGland/esophagus/2018-12-20/791683-19-0;4.ndpi | 0 | 0.20288235 | 0.057168627 | 0.031592157 |
| 773 | /data5/data5/dongzhong/LymphGland/esophagus/2018-12-20/792225-15-0;2.ndpi | 0 | 0.32061569 | 0.324886275 | 0.040882353 |
| 774 | /data5/data5/dongzhong/LymphGland/esophagus/2018-12-20/792483-13-0;3.ndpi | 0 | 0.1705098 | 0.000207843 | 0.011156863 |
| 775 | /data5/data5/dongzhong/LymphGland/esophagus/2018-12-20/792915-15-0;1.ndpi | 0 | 0.12920392 | 0 | 0.001458824 |
| 776 | /data5/data5/dongzhong/LymphGland/esophagus/2018-12-20/793059-10-0;2.ndpi | 0 | 0.2371451 | 0.0076 | 0.01305098 |
| 777 | /data5/data5/dongzhong/LymphGland/esophagus/2018-12-20/793167-14-0;1.ndpi | 0 | 0.37785098 | 0.008521569 | 0.030345098 |
| 778 | /data5/data5/dongzhong/LymphGland/esophagus/2018-12-20/793533-17-0;1.ndpi | 0 | 0.04261569 | 0 | 0.002988235 |
| 779 | /data5/data5/dongzhong/LymphGland/esophagus/2018-12-20/793834-18-0;5.ndpi | 0 | 0.81128627 | 0.623678431 | 0.595568627 |
| 780 | /data5/data5/dongzhong/LymphGland/esophagus/2018-12-20/794196-12-0;5.ndpi | 0 | 0.23765882 | 0.768729412 | 0.090141176 |
| 781 | /data5/data5/dongzhong/LymphGland/esophagus/2018-12-20/794683-21-0;3.ndpi | 0 | 0.49619608 | 0.317447059 | 0.065972549 |
| 782 | /data6/医科院肿瘤医院正常和其他/2018-11-02/淋巴(食管)正常/794889-8-0;1.ndpi | 0 | 0.098 | 0.000364706 | 0 |
| 783 | /data5/data5/dongzhong/LymphGland/esophagus/2018-12-20/795608-18-0；6.ndpi | 0 | 0.46686275 | 0.428737255 | 0.052603922 |
| 784 | /data5/data5/dongzhong/LymphGland/esophagus/2018-12-20/795928-12-0；2.ndpi | 0 | 0.62878824 | 0.121043137 | 0.33667451 |
| 785 | /data6/医科院肿瘤医院正常和其他/2018-11-02/淋巴(食管)正常/795996-13-0;2.ndpi | 0 | 0.02343922 | 0.004658824 | 0.008866667 |
| 786 | /data5/data5/dongzhong/LymphGland/esophagus/2018-12-20/796186-19-0;5.ndpi | 0 | 0.48754902 | 0.10034902 | 0.050662745 |
| 787 | /data5/data5/dongzhong/LymphGland/esophagus/2018-12-20/796657-21-0;2.ndpi | 0 | 0.43654118 | 0.097513725 | 0.143996078 |
| 788 | /data5/data5/dongzhong/LymphGland/esophagus/2018-12-20/797267-2-0;2.ndpi | 0 | 0.41204706 | 0.278682353 | 0.163772549 |
| 789 | /data5/data5/dongzhong/LymphGland/esophagus/2018-12-20/797479-11-0;2.ndpi | 0 | 0.46882745 | 0.140458824 | 0.008831373 |
| 790 | /data5/data5/dongzhong/LymphGland/esophagus/2018-12-20/797701-15-0;5.ndpi | 0 | 0.55818431 | 0.369752941 | 0.04467451 |
| 791 | /data6/医科院肿瘤医院正常和其他/2018-11-09/淋巴（食管）正常/798305-12-0;5.ndpi | 0 | 0.1716902 | 0.001839216 | 0.004447059 |
| 792 | /data6/医科院肿瘤医院正常和其他/2018-11-09/淋巴（食管）正常/798347-6-0;1.ndpi | 0 | 0.79603922 | 0.337129412 | 0.000109804 |
| 793 | /data5/data5/dongzhong/LymphGland/esophagus/2018-12-20/798348-19-0；2.ndpi | 0 | 0.88883137 | 0.219945098 | 0.027984314 |
| 794 | /data5/data5/dongzhong/LymphGland/esophagus/2018-12-20/798363-19-0；2.ndpi | 0 | 0.29194118 | 0.151894118 | 0.080988235 |
| 795 | /data5/data5/dongzhong/LymphGland/esophagus/2018-12-20/798467-15-0；1.ndpi | 0 | 0.20123137 | 0.281658824 | 0.026976471 |
